# Progress in Cu‐Based Catalyst Design for Sustained Electrocatalytic CO_2_ to C_2+_ Conversion

**DOI:** 10.1002/advs.202416597

**Published:** 2025-02-27

**Authors:** Dan Li, Jinyuan Liu, Bin Wang, Chao Huang, Paul K. Chu

**Affiliations:** ^1^ Department of Physics Department of Materials Science and Engineering and Department of Biomedical Engineering City University of Hong Kong Kowloon Hong Kong China

**Keywords:** C_2+_ products, C─C coupling, charged Cu species, CO_2_ reduction, Cu‐based catalysts, reconstruction

## Abstract

The electrocatalytic conversion of CO_2_ into valuable multi‐carbon (C_2+_) products using Cu‐based catalysts has attracted significant attention. This review provides a comprehensive overview of recent advances in Cu‐based catalyst design to improve C_2+_ selectivity and operational stability. It begins with an analysis of the fundamental reaction pathways for C_2+_ formation, encompassing both established and emerging mechanisms, which offer critical insights for catalyst design. In situ techniques, essential for validating these pathways by real‐time observation of intermediates and material evolution, are also introduced. A key focus of this review is placed on how to enhance C_2+_ selectivity through intermediates manipulation, particularly emphasizing catalytic site construction to promote C─C coupling via increasing ^*^CO coverage and optimizing protonation. Additionally, the challenge of maintaining catalytic activity under reaction conditions is discussed, highlighting the reduction of active charged Cu species and materials reconstruction as major obstacles. To address these, the review describes recent strategies to preserve active sites and control materials evolution, including novel catalyst design and the utilization and mitigation of reconstruction. By presenting these developments and the challenges ahead, this review aims to guide future materials design for CO_2_ conversion.

## Graphical abstract

This review highlights recent advancements pertaining to the design of Cu‐based catalysts pertaining to the electrocatalytic reduction of CO_2_ into multi‐carbon (C_2+_) products, with a focus on how to overcome challenges related to product selectivity and long‐term stability. Key strategies include manipulating reaction intermediates to enhance C─C coupling and managing materials evolution to preserve active sites. In situ techniques and reaction mechanisms are also discussed to provide insights for future development of high‐performance catalysts.

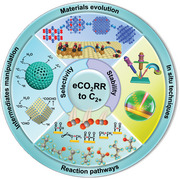

## 1 Introduction

**Figure 1:**
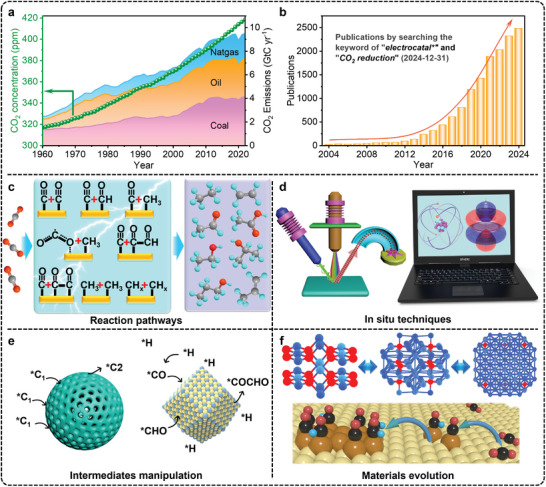
a) Global atmospheric CO_2_ concentration and annual fossil fuel emissions from 1960 to 2023 (Data from Global Monitoring Laboratory and Global Carbon Budget Office). b) Number of publications compiled by the Web of Science by using keywords “electrocatal*” and “CO_2_ reduction”. Schematics of c) possible eCO_2_RR pathways, d) in situ characterization techniques, e) intermediates manipulation, and f) materials evolution.

Rapid industrialization and societal advancement have led to excessive use of fossil fuels, resulting in significant CO_2_ emissions that contribute to environmental deterioration, such as the greenhouse effect and ocean acidification.^[^ ^1^, ^2^, ^3^ ^]^ Since the onset of the industrial era in 1750, atmospheric CO_2_ levels have risen from 278 to 421 ppm in 2023 (**Figure** **1**a). Despite global efforts like the Paris Agreement to curb carbon emissions, fossil fuels continue to dominate energy consumption. In response, China has outlined a clear strategy to achieve carbon peaking by 2030 and carbon neutrality by 2060, with plans to increase non‐fossil energy consumption to 25% by 2030 and over 80% by 2060. This highlights the nation's strong commitment to transitioning toward a green, low‐carbon economy. Expanding renewable energy sources, including wind, solar, and tidal energy, has become a critical priority, especially with the ongoing efforts to reduce electricity costs. In this context, the electrocatalytic reduction reaction (eCO_2_RR) presents a promising approach to converting CO_2_ into valuable chemicals and fuels using renewable electricity, offering a sustainable and scalable solution.^[^ ^4^, ^5^ ^]^ Moreover, eCO_2_RR operates under mild conditions, such as room temperature and atmospheric pressure, without generating polluting by‐products, making it viable for industrial‐scale applications.^[^ ^6^, ^7^ ^]^ However, this process faces significant challenges, particularly in improving product selectivity, catalyst stability, and energy efficiency. Research interest in addressing these challenges has surged, as evidenced by the exponential rise in publications on “electrocatalysis” and “CO_2_ reduction” in the Clarivate Web of Science database (Figure 1b).

The eCO_2_RR has been explored since the 19th century, with significant advancements occurring after the turn of the 21st century.^[^ ^8^, ^9^, ^10^ ^]^ Early developments in metal and alloy‐based systems enabled the efficient reduction of CO_2_ into hydrocarbons, sparking a rapid growth in research. During this initial phase, the focus was predominantly on the production of single‐carbon (C_1_) products, such as carbon monoxide (CO) and formic acid (HCOOH), which have achieved notable significance in selectivity and efficiency.^[^ ^11^, ^12^ ^]^ However, the production of multi‐carbon (C_2+_) products, such as ethylene (C_2_H_4_), ethanol (C_2_H_5_OH), and acetic acid (CH_3_COOH), is of greater interest due to their higher energy density and economic value.^[^ ^2^, ^13^, ^14^, ^15^ ^]^ For instance, C_2_H_4_ is a key petrochemical product, while C_2_H_5_OH and CH_3_COOH are widely used in industries such as pharmaceuticals, agriculture, and food production production.^[^ ^7^, ^16^, ^17^, ^18^, ^19^ ^]^ As a result, recent research has shifted toward enhancing the selectivity and efficiency of C_2+_ production. Notably, from the 2020s, significant progress has been made in understanding the underlying mechanisms and optimizing catalysts for C_2+_ product generation, positioning this area as a key focus for future advancements in CO_2_ conversion technologies.

The reduction of CO_2_ to C_2+_ products involves complex multi‐step proton‐coupled electron transfer (PCET) processes, requiring the adsorption and activation of CO_2_, efficient mass transfer of C_1_ intermediates, precise C─C coupling, and product release.^[^ ^8^, ^20^, ^21^ ^]^ These steps demand catalysts with highly functional active sites and a deeper understanding of the underlying mechanisms. In recent years, advances in computational modeling and theoretical studies have revolutionized catalyst design, moving away from the traditional trial‐and‐error approach. Researchers now leverage theoretical insights to create more efficient and targeted catalysts. Among the various challenges in catalyst design, one of the most significant is the stabilization and coupling of ^*^C_1_ intermediates on catalyst surfaces, as C─C coupling involves thermochemical steps that do not rely on electron or proton transfer. This presents a significant hurdle for traditional catalyst designs that use external electrical energy to induce surface polarization. Cu‐based catalysts have emerged as leading candidates because of their moderate ^*^C_1_ intermediate adsorption capabilities, providing a platform for effective C─C coupling to produce C_2+_ products.^[^ ^10^, ^14^, ^17^, ^22^, ^23^ ^]^ However, the sluggish C─C coupling kinetics necessitates precise catalyst design to incorporate various functional catalytic sites. The goal is to either enrich the local concentration of ^*^C_1_ intermediates, primarily ^*^CO, thereby increasing the likelihood of C─C coupling, or modify the coupling mode to a more favorable one. In fact, recent studies have identified alternative coupling modes, such as ^*^CO─^*^COH, ^*^CO─^*^CHO, ^*^CHO─^*^CHO, ^*^CO─^*^CH_x_, and so on, which exhibit lower energy barriers than the conventional ^*^CO–^*^CO coupling, depending on the catalyst's structure and composition.^[^ ^9^, ^13^, ^24^, ^25^, ^26^, ^27^, ^28^, ^29^ ^]^ Another critical challenge is maintaining the stability of the catalyst. For instance, positively charged Cu species (Cu^δ+^) are crucial to C─C coupling and C_2+_ product generation, but they tend to reduce under the strong reducing conditions of eCO_2_RR. Furthermore, in situ reconstruction during the reaction can alter the catalyst's structure and composition, impacting its long‐term activity. Understanding and managing these dynamic changes is vital for Cu‐based catalysts that maintain their performance over extended periods.

In recent years, several reviews have explored the factors influencing the selectivity of Cu‐based catalysts, such as composition, morphology, crystal structure, and surface characteristics, as well as topics like oxidation state modulation, electrolyte and electrolyzer design, and solid‐electrolyte interface regulation.^[^ ^1^, ^3^, ^6^, ^8^, ^10^, ^23^, ^30^, ^31^, ^32^, ^33^, ^34^, ^35^ ^]^ While these reviews have significantly advanced our understanding, challenges remain. Achieving continuous, high selectivity for C_2+_ products remains elusive, with ongoing debates about the dynamic intermediates driving product formation and the catalyst evolution affecting operational stability. Moreover, emerging tools such as machine learning, theoretical simulations, and in situ techniques are evolving rapidly, offering fresh insights into catalyst design and reaction behavior. One critical yet underexplored area is catalyst stability, which is essential for industrial applications.^[^ ^36^, ^37^ ^]^ Many promising Cu‐based catalysts degrade over time, thus compromising the long‐term viability. Ensuring sustained generation of C_2+_ products while maintaining selectivity over time remains a significant hurdle. Striking a balance between selectivity and stability is vital for advancing these catalysts toward industrial‐scale use, highlighting the timeliness and relevance of this review.

This review offers a unified framework for understanding C_2+_ selectivity by focusing on the manipulation of reaction intermediates, particularly through ^*^CO enrichment and intermediate protonation at specific catalytic sites to promote C─C coupling. In addition, it provides a comprehensive overview of material evolution and its impact on catalyst stability. By integrating insights from both the selectivity and stability perspectives, this review advances the development of Cu‐based catalysts for industrial applications and offers potential solutions for other electrochemical systems. It begins by discussing the potential reaction pathways for C_2+_ products (Figure 1c) and examining how in situ and quasi‐in situ characterization techniques contribute to understanding structural evolution and intermediate formation (Figure 1d). The review then explores catalyst design strategies aimed at manipulating intermediates to promote C─C coupling (Figure 1e), followed by approaches to manage in situ material evolution to protect active sites or control materials reconstruction for ensuring catalyst stability (Figure 1f). This comprehensive overview aims to provide guidance and insights to advance the development of Cu‐based catalysts for industrial applications while deepening the understanding of the underlying mechanisms driving CO_2_ conversion to C_2+_ products in both intermediates and materials aspects.

## 2 Pathways of CO_2_ Reduction to C_2+_ Products

CO_2_ is a highly stable molecule due to its centrosymmetric linear structure, with two identical C═O bonds, each measuring 116.3 pm in length and a bond energy of 750 kJ mol^−1^.^[^ ^38^ ^]^ This stability arises from carbon *sp* hybridization, forming two sigma (σ) bonds with the oxygen atoms.^[^ ^39^ ^]^ Additionally, the unhybridized p orbitals form off‐plane pi (π) bonds with the oxygen atoms, resulting in a low reactivity due to the electron cloud being concentrated around the oxygen atoms.^[^ ^40^ ^]^ Despite this, the polarity of the C═O bond leaves the central carbon atom electron‐deficient, making it reactive as an electrophile when interacting with nucleophiles or electron‐donating groups.^[^ ^41^ ^]^ In eCO_2_RR, which primarily occurs at the catalyst‐electrolyte interface or the three‐phase interface of CO_2_ gas, catalyst, and electrolyte at the cathode, CO_2_ reduction proceeds through several key steps.^[^ ^42^, ^43^, ^44^, ^45^ ^]^ Initially, CO_2_ adsorbs onto the catalyst, followed by activation and cleavage of the C═O bond. The first product, typically a C_1_ compound, is formed through protonation. The formation of C_2_ products requires stabilization of ^*^C_1_ intermediates, leading to C─C coupling and further protonation. For C_3_ products, additional C─C coupling and protonation of the ^*^C_2_ intermediates are needed.^[^ ^20^, ^34^, ^46^, ^47^ ^]^ However, both processes are highly complex, involving multiple electron transfer steps, numerous intermediates, and competition from the hydrogen evolution reaction (HER).^[^ ^22^, ^48^, ^49^ ^]^

### 2.1 Formation of C_2_ Products

The key step in converting C_1_ to C_2+_ products in eCO_2_RR is the C─C coupling process, which relies on the stability of various ^*^C_1_ intermediates.^[^ ^6^, ^7^, ^50^, ^51^, ^52^, ^53^, ^54^ ^]^ Among these intermediates, ^*^CO is commonly the most abundant due to its lower energy barrier for formation compared to others.^[^ ^55^, ^56^, ^57^, ^58^ ^]^ Intermediates like ^*^CHO, ^*^COH, and ^*^CH_x_ require further PCET reactions from ^*^CO, which are more energy‐intensive to produce.^[^ ^58^, ^59^, ^60^, ^61^ ^]^ However, unlike conventional reactions driven by electron or proton transfer, C─C coupling involves thermochemical steps, making it more challenging to control with standard techniques like catalyst surface polarization using external electrical energy. The formation of intermediates like ^*^CHO, ^*^COH, and ^*^CH_x_ expands the possible C─C coupling modes, such as ^*^CO─^*^CHO,^[^ ^62^, ^63^, ^64^, ^65^, ^66^, ^67^ ^] *^CO─^*^COH,^[^ ^68^, ^69^, ^70^, ^71^, ^72^, ^73^, ^74^, ^75^ ^] *^CHO─^*^CHO,^[^ ^76^ ^] *^COH─^*^COH,^[^ ^77^ ^]^ and ^*^CH_x_─^*^CO,^[^ ^78^, ^79^ ^]^ etc.^[^ ^80^, ^81^, ^82^, ^83^, ^84^ ^]^ This variety enhances the likelihood and diversity of C─C coupling, making the process more favorable in certain cases. Nonetheless, specific C─C coupling pathways depend heavily on the types and concentrations of these intermediates.

**Figure 2:**
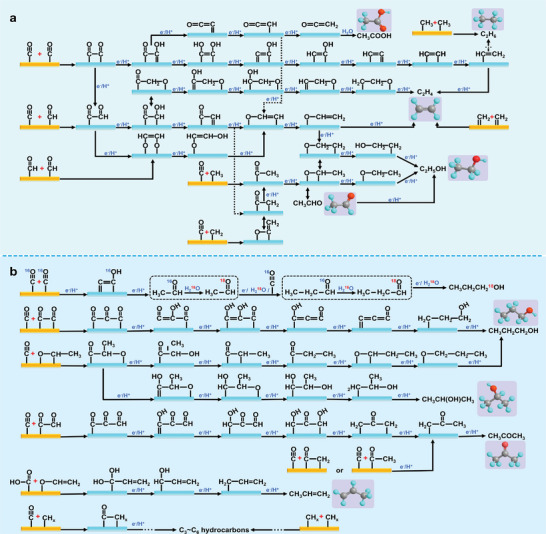
Pathways for the generation of a) C_2_ and b) C_3_ products from CO_2_ reduction.

Among the various C_2+_ products, C_2_H_4_ is the most common in eCO_2_RR.^[^ ^7^, ^85^, ^86^, ^87^, ^88^, ^89^ ^]^ **Figure** **2**a illustrates the potential pathways for C_2_H_4_ production via C─C coupling, including ^*^CO─^*^CO, ^*^CO─^*^COH, and ^*^CH_2_─^*^CH_2_ interactions.^[^ ^90^, ^91^, ^92^, ^93^, ^94^, ^95^ ^]^ The ^*^CO─^*^CHO intermediate can also form via a one‐step PCET reaction from ^*^CO─^*^CO. Finally, the C_2_H_4_ is produced through hydro‐dehydration of the coupled C atoms, with retention of the C═C double bond. In earlier studies, Hori et al., proposed that the ^*^CO intermediate initially undergoes protonation in two steps to form ^*^CH_2_, followed by the coupling of two ^*^CH_2_ units to produce C_2_H_4_.^[^ ^96^ ^]^ This was one of the initial mechanisms explored for C_2_H_4_ production in CO_2_ reduction. Another widely recognized mechanism is the dimerization of ^*^CO to form a ^*^COCO intermediate, which serves as the rate‐determining step in most cases.^[^ ^50^, ^97^, ^98^, ^99^ ^]^ In this mechanism, the C─C single bond in the ^*^COCO intermediate is converted into a C═C double bond via a McMurry coupling reaction, leading to the cleavage of the C─O bond and forming enediol or enediol ester, which is then further protonated to produce C_2_H_4_. Additionally, recent studies have also proposed alternative coupling mechanisms that involve different ^*^C_1_ intermediates, such as the ^*^CO─^*^CHO and ^*^CHO─^*^CHO coupling pathways, which are more favorable at certain sites.^[^ ^77^, ^100^, ^101^, ^102^, ^103^, ^104^, ^105^ ^]^

C_2_H_6_, another important C_2_ product, can be formed either through two PCET reactions from ^*^CH_2_═^*^CH_2_ or via the polymerization of two ^*^CH_3_ groups.^[^ ^106^, ^107^, ^108^ ^]^ However, the polymerization of ^*^CH_x_ intermediates to generate C_2_ products requires a high surface coverage of ^*^CH_x_.^[^ ^109^, ^110^ ^]^ Since ^*^CH_x_ is derived from ^*^CO through multiple PCET steps and CH_4_ formation is generally more favorable, achieving this high coverage presents significant challenges. Consequently, the polymerization of ^*^CH_x_ is a less common pathway for C_2_ production, and the generation of C_2_H_6_ remains a significant challenge.

The formation of C_2_H_5_OH occurs after the coupling of ^*^CO─^*^CO or ^*^CO─^*^CHO intermediates, followed by a series of PCET reactions that preserve the ─OH functional group attached to a single‐bonded carbon atom.^[^ ^111^, ^112^, ^113^, ^114^, ^115^, ^116^, ^117^, ^118^, ^119^, ^120^, ^121^ ^]^ In contrast, if the ─CHO group is retained instead of ─OH, the product formed is acetaldehyde.^[^ ^122^, ^123^, ^124^, ^125^, ^126^, ^127^, ^128^, ^129^ ^]^ For example, Sun et al., proposed a catalyst that enhances CO_2_ adsorption to promote ^*^CO─^*^CO coupling while preventing full hydrogenation of C_2_H_4_ intermediates, enabling better control over the reduction of ^*^C_2_ intermediates to acetaldehyde.^[^ ^122^ ^]^ Ager et al., used proton‐transfer‐reaction time‐of‐flight mass spectrometry to confirm that acetaldehyde can be produced via ^*^CO─^*^CH_2_ coupling followed by PCET reactions.^[^ ^124^ ^]^ However, they found that acetaldehyde is not the primary intermediate in C_2_H_5_OH or C_2_H_4_ formation. Instead, the reduction of propionaldehyde was identified as the main pathway for producing 1‐propanol.

Through a review of the literature, we have found that the formation pathways of C_2_ products are not singular. Many of these products share common ^*^C_2_ intermediates, leading to competition between the selectivity for different C_2_ products. For CH_3_COOH production, Jiao et al., have proposed a widely accepted mechanism.^[^ ^130^, ^131^ ^]^ After ^*^CO─^*^CO coupling, one carbon atom retains an oxygen atom while the other undergoes two PCET steps to form the key intermediate ketene (O═C═CH_2_). Ketene has weak adsorption on Cu‐based electrocatalysts and, after desorbing, reacts with nucleophilic OH⁻ ions to form acetate. The formation of acetate depends on the concentration of solvated ketene and hydroxide on the catalyst surface, both governed by mass transport dynamics.^[^ ^132^ ^]^ If both carbon atoms undergo PCET reactions after ^*^CO─^*^CO coupling, C_2_H_4_ or C_2_H_5_OH will be produced along alternative pathways.

### 2.2 Formation of C_3_ Products

Compared to the C_2_ products, the preparation of the C_3_ products via eCO_2_RR involves a more complex two‐step C─C coupling process (Figure 2b), significantly increasing the reaction complexity. Common C_3_ products include n‐propanol and acetone.^[^ ^81^, ^133^, ^134^, ^135^, ^136^, ^137^, ^138^, ^139^, ^140^, ^141^ ^]^ The coupling of ^*^CO with ^*^COCO or ^*^OCHCH_3_, followed by a multi‐step PCET reaction, leads to intermediates such as ^*^CH_2_CH_2_CH_2_OH or ^*^OCH_2_CH_2_CH_3_, ultimately resulting in n‐propanol. When the ─OH functional group is retained on the middle carbon atom, the product is isopropanol.^[^ ^142^ ^]^ However, the formation of isopropanol has been less frequently reported. The mechanism for n‐propanol and isopropanol formation has been largely derived from theoretical calculations, which focus on C─C coupling and PCET but overlook the source of oxygen in these C_3_ alcohol products. Jiao et al., have introduced flow electrolysis mass spectrometry by integrating gas diffusion electrode design and H_2_^18^O isotope labeling, which reveal that oxygen in the acetaldehyde intermediate originates from CO, while C_2_H_5_OH and n‐propanol mainly incorporate oxygen from the solvent.^[^ ^143^ ^]^ This finding aligns with Ager et al., earlier work, where C^16^O reduction in an H_2_^18^O electrolyte resulted in a high proportion of ^18^O‐containing C_2_H_5_OH and n‐propanol.^[^ ^144^ ^]^ For acetone production, coupling ^*^CO with ^*^COCOH or ^*^COCH_x_, followed by complete hydrogenation of both terminal carbons while retaining the central C═O bond is required.^[^ ^137^, ^145^ ^]^ In this process, protonation of the tertiary carbon's C═O group can yield isopropanol, a competitive reaction to acetone formation. Hydroprotonation of all oxygen‐containing functional groups on the ^*^C_3_ intermediate results in polycarbon hydrocarbon products such as propylene and propane.^[^ ^146^, ^147^ ^]^ However, due to the lower energy barrier of oxygen retention in multi‐step PCET processes, producing oxygenated C_3_ compounds is more feasible. Currently, C_3_‐C_6_ multi‐carbon hydrocarbon products can be synthesized through the coupling of ^*^COOH and ^*^OCHCH_2_ or through multi‐step ^*^CH_x_ coupling followed by PCET.^[^ ^82^, ^148^, ^149^, ^150^, ^151^, ^152^, ^153^ ^]^ The preparation of C_3_ products via eCO_2_RR involves numerous intermediates and isomerization steps. While some intermediates have been confirmed through in situ spectroscopy, most remain speculative, based on theoretical calculations. Thus, further investigation into the reaction mechanisms for C_3_ product formation is essential for guiding the design of more efficient catalysts and reactors.

Exploring the mechanisms underlying eCO_2_RR to C_2+_ products remains a key focus in current research, yet significant challenges persist. Most proposed mechanisms rely heavily on computational calculations complemented by in situ data. While experimental studies often succeed in detecting reaction intermediates, they frequently fall short in capturing their dynamic evolution, leaving crucial aspects of the reaction pathway unclear. To address this, future research must prioritize improving the time‐scale resolution for intermediate capture and identification, enabling a more detailed and accurate understanding of reaction mechanisms. In addition, the exploration of CO_2_‐to‐C_2+_ pathways is hindered by the inherently low efficiency of C─C bond formation and the complexity arising from various coupling modes. C─C coupling typically involves the interaction of multiple catalytic sites, adding variability and uncertainty to the reaction mechanism. This challenge is evident in both theoretical calculations and the experimental identification of key intermediates. Advancing this field requires precise atomic‐scale identification of intermediates and the development of accurate reaction models, which are essential for unraveling the intricate details of C─C coupling and optimizing catalyst design.

## 3 Advanced In Situ Monitoring Techniques

In the eCO_2_RR process, understanding how products form, how intermediates behave, and how catalysts perform are essential for optimizing catalytic efficiency and operational stability. The formation of C_2+_ products is notably more complex than that of C_1_ products, thus making the reaction interface significantly more intricate.^[^ ^17^ ^]^ To address this complexity, in situ monitoring techniques are increasingly combined with theoretical calculations to uncover reaction pathways and catalyst evolution that would otherwise remain obscured. With regard to product selectivity, different products are formed via distinct electron and proton transfer processes involving the coupling of various intermediates. These intermediates must be carefully analyzed to fully understand the underlying mechanisms. Key intermediates, such as ^*^COCH_x_ and ^*^OCCOH, reveal the C─C coupling essential for C_2+_ product formation, while intermediates like ^*^CO and ^*^CHO can indicate potential pathways for both C─C coupling and C_1_ product formation. To this end, advanced spectroscopy techniques such as Raman scattering and Fourier transform infrared (FTIR) spectroscopy are commonly employed to detect crucial intermediates like ^*^COOH, ^*^OCHO, ^*^CO, ^*^CHO, and ^*^OCCOH, offering valuable insights into these processes.^[^ ^154^, ^155^, ^156^, ^157^ ^]^ For instance, in situ spectroscopy can detect fleeting intermediates like ^*^CO dimers or ^*^COCHO, crucial for understanding C─C coupling mechanisms that drive C_2+_ product formation.^[^ ^75^, ^158^, ^159^, ^160^ ^]^ Beyond tracking intermediates, in situ techniques monitor catalyst evolution in real time under operational conditions, which is important in tracing the operational stability of the catalyst. Unlike ex situ methods, which offer only post‐reaction snapshots, in situ techniques observe dynamic changes in the catalyst's surface structure and oxidation states during the reaction.^[^ ^161^ ^]^ Tools such as in situ microscopy and in situ X‐ray absorption spectroscopy (XAS) reveal how the catalyst evolves, providing insights into structural and chemical transformations that occur throughout the process. This section reviews the application and challenges of in situ characterization techniques in exploring eCO_2_RR, emphasizing their role in revealing reaction pathways and catalyst evolution. Key methods include in situ microscopy, in situ X‐ray diffraction (XRD), in situ X‐ray spectroscopy, in situ FTIR and Raman scattering spectroscopy, and isotope tracing. These techniques provide critical data on catalyst morphology, composition, and the intermediates that drive reactions.

### 3.1 In Situ Microscopy

Liquid‐phase transmission electron microscopy (TEM) enables real‐time observation of nanostructure changes at the single‐particle level, providing insights into the dynamic behavior of materials in a liquid environment. By incorporating thin‐film electrodes on microfluidic chips, electrochemical experiments can be conducted within the TEM, known as electrochemical TEM (EC‐TEM).^[^ ^162^, ^163^, ^164^ ^]^ This method is instrumental in addressing key challenges, such as catalyst stability under reaction conditions. Unlike ex situ TEM such as identical location TEM, which captures post‐reaction structural changes, EC‐TEM allows real‐time monitoring of morphological evolution and element distribution, offering critical insights into surface transformation mechanisms.^[^ ^165^, ^166^, ^167^, ^168^ ^]^

**Figure 3:**
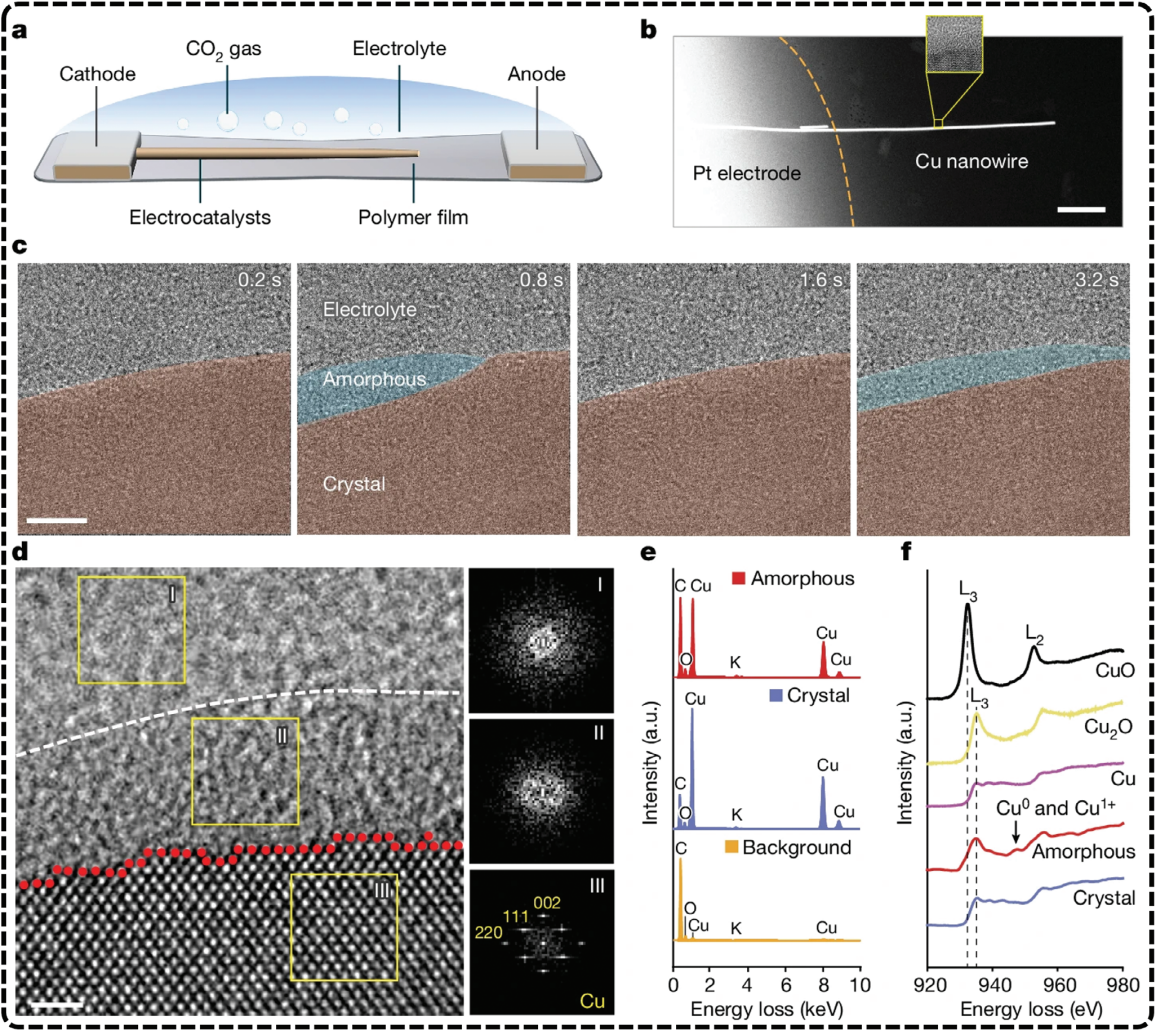
Experimental setup and characterization of the fluctuating liquid‐like amorphous interphase. Reproduced with permission.^[^ ^169^ ^]^ Copyright 2024, Springer Nature. a) Schematic of a perspective view of the sample area. b) HAADF image of a Cu nanowire suspended on the Pt electrode. The inset highlights the ESLI. c) A TEM image sequence shows the emergence and fluctuations of the amorphous interphase. d) HRTEM image and corresponding fast Fourier transform patterns (I, II, III) show distinguishing structure and contrast features of the amorphous interphase, crystal Cu, and electrolyte. e) EDS spectra of amorphous interphase, crystal Cu, and electrolyte. f) Cu L_2,3_ edge EELS spectra of different phases. Scale bars, 500 nm (b), 5 nm (c), 1 nm (d). a.u., arbitrary units.

**Figure 4:**
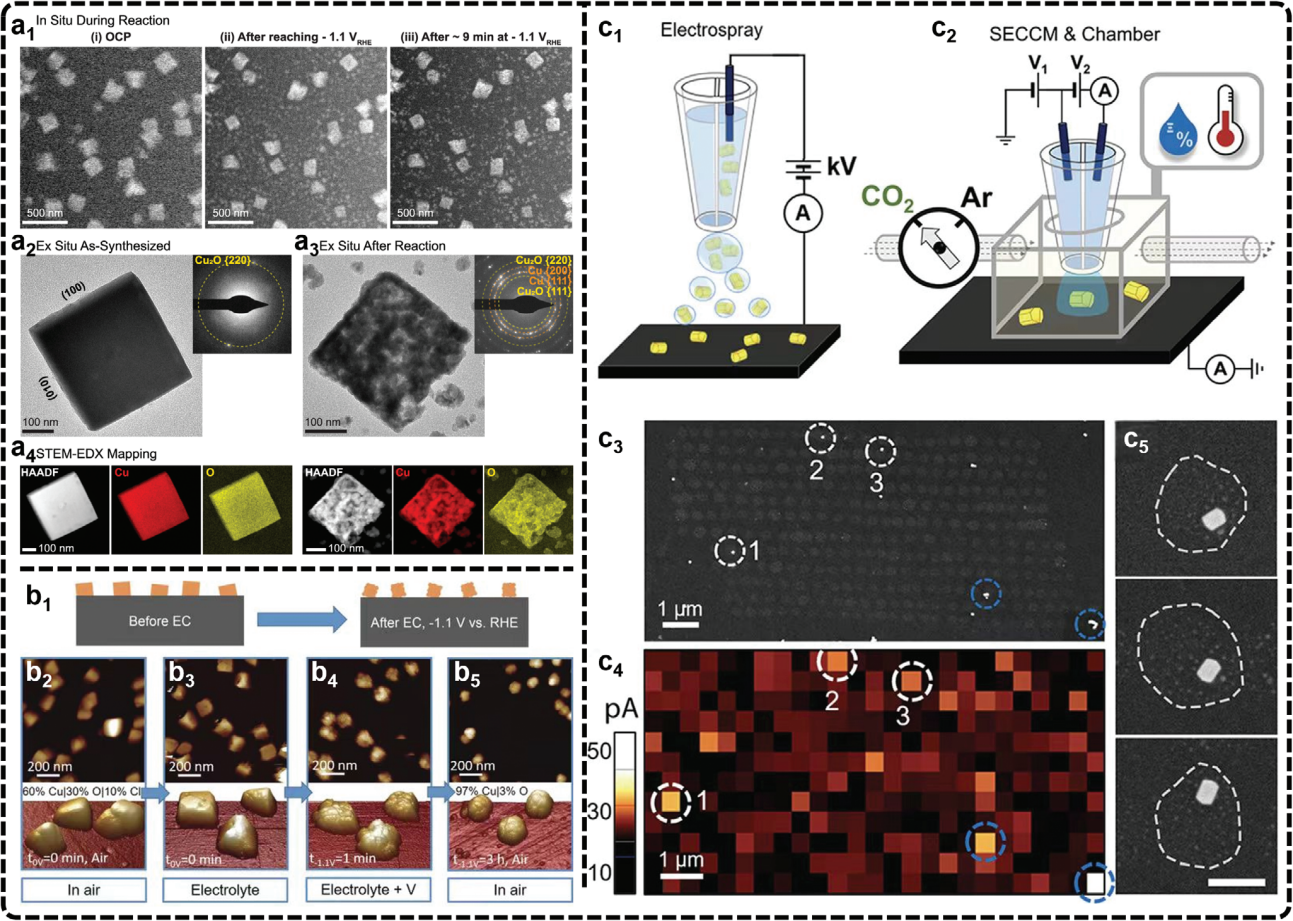
a) Morphology of Cu_2_O cubes and their evolution during eCO_2_RR. Adapted under terms of the CC‐BY license.^[^ ^171^ ^]^ Copyright 2021, Grosse et al. a_1_) Sequence of images illustrating the morphological changes observed in ≈170 nm cubes after i) introducing CO_2_ saturated 0.1 m KHCO_3_ under OCP, ii) while applying a reductive potential of −1.1 V_RHE_ and iii) after ≈9 min at −1.1 V_RHE_, where a large bubble formed. The image sequences were all acquired with an electron flux of 1.7 e^−^ Å^−2^ s^−1^. Comparison of Cu_2_O cubes a_2_) before and a_3_) after reaction using ex situ TEM imaging and electron diffraction (upper right inserts). a_4_) STEM‐EDX maps of the same cubes showing the Cu and O signals. b) Morphological evolution of Cu cube catalysts by AFM. Adapted with permission.^[^ ^180^ ^]^ Copyright 2018, John Wiley and Sons. b_1_) Schematic representation of the morphological evolution of Cu cube catalysts. b_2_–b_5_) AFM images of Cu cubes electrodeposited on highly oriented pyrolytic graphite acquired in the air b_2_), and operando EC‐AFM measurements in a CO_2_‐saturated 0.1 m KHCO_3_ aqueous solution at OCP b_3_), at −1.1 V versus RHE in the same electrolyte for 1 min b_4_), and after 3 h under the same conditions as in b_4_) and subsequent air exposure b_5_). c) SECM results of Au eCO_2_RR catalysts. Adapted with permission.^[^ ^188^ ^]^ Copyright 2022, American Chemical Society. Schematic depiction of c_1_) the electrospray setup for Au nanocrystal deposition and c_2_) environment‐controlled SECCM setup. c_3_) SEM image of Au TDP deposited on GC substrate and c_4_) corresponding SECM map at −0.9 V versus RHE under an Ar atmosphere. The scan rate of SECM LSV was 1.0 V s^−1^. Both isolated Au TDP (white dashed circles) and clusters of TDP (blue dashed circles) are highlighted. c_5_) High‐magnification SEM images of individual Au TDP and electrolyte droplet footprints (white dashed contours) showing fully encapsulated Au nanocrystals during SECM measurements. Scale bar: 200 nm.

Recently, Zheng et al., introduced an advanced polymer electrochemical liquid cell for TEM, allowing real‐time monitoring of atomic dynamics at electrified solid‐liquid interfaces (ESLI) during Cu‐catalyzed eCO_2_RR.^[^ ^169^ ^]^ Their findings reveal a fluctuating, liquid‐like amorphous interphase that undergoes reversible transitions between crystalline and amorphous states (**Figure** **3**). This interphase flows along the Cu surface, mediating surface restructuring and mass loss. By combining real‐time imaging with theoretical calculations, the study uncovered an amorphization‐driven restructuring mechanism caused by charge‐activated surface reactions with the electrolyte, providing deep insight into atomic dynamics in solid‐liquid interfaces. This progress offers a valuable tool for exploring dynamic processes that are key to improving catalyst stability and performance. Another notable application of EC‐TEM is described in the works of Cuenya et al. They studied cubic copper oxide particles during eCO_2_RR and demonstrated the morphological evolution of these particles during synthesis from copper sulfate solutions together with the subsequent shape changes in the CO_2_‐saturated KHCO_3_ solution under reduction potentials.^[^ ^170^ ^]^ In a related study, Cuenya's group explored the real‐time restructuring of Cu oxide cubes during eCO_2_RR, observing fragmentation, re‐deposition of new nanoparticles, catalyst detachment, and aggregation as a function of potential (**Figure** **4**a).^[^ ^171^ ^]^ These studies reveal how nanoparticle re‐deposition and detachment influence catalyst reactivity, with the increased metal surface area from re‐deposited nanoparticles enhancing both C_2+_ selectivity and stability. In addition, Yang et al., recently utilized in situ electrochemical scanning transmission electron microscopy (EC‐STEM) and 4D STEM to track the structural evolution of Cu nanocatalysts during eCO_2_RR.^[^ ^172^ ^]^ Their study reveals that a 7 nm Cu nanoparticle ensemble transforms into metallic nanograins during electrolysis and is subsequently oxidized to single‐crystal Cu_2_O nanocubes post‐reaction. In combination with in situ XAS, they confirmed that metallic Cu nanograins, rich in grain boundaries, acted as undercoordinated active sites for C─C coupling. The study also demonstrates that smaller Cu nanoparticles, with higher fractions of active nanograins, exhibited significantly enhanced C_2+_ selectivity compared to larger particles. Despite some recent advances, currently, the use of EC‐TEM in eCO_2_RR research is still limited due to the demanding equipment requirements and operational complexity. Key challenges include the need for ultra‐high vacuum, designing functional in situ TEM cells, and the risk of electron beam‐induced damage to catalysts, all of which can affect measurement accuracy under real electrocatalytic conditions.^[^ ^173^, ^174^, ^175^ ^]^ Despite these hurdles, EC‐TEM remains essential for studying catalyst structural evolution in real time.

Scanning probe microscopy encompasses a range of techniques used to explore surface topography at the nanoscale, including atomic force microscopy (AFM), scanning tunneling microscopy (STM), and scanning near‐field optical microscopy, enabling nanoscale surface topography analysis through physical interactions between a sharp probe and the sample surface.^[^ ^176^ ^]^ Initially designed for vacuum use, STM has been adapted for ambient and liquid conditions, but AFM overcomes the STM limitation of requiring conductive or semiconducting surfaces.^[^ ^177^ ^]^ In eCO_2_RR research, electrochemical AFM (EC‐AFM) has been pivotal in monitoring catalyst dynamics under reaction conditions.^[^ ^178^, ^179^ ^]^

For example, one study observes significant alterations to Cu cubes on carbon electrodes during the reaction (Figure 4b), including coarsening (Figure 4b_3_), loss of (100) facets (Figure 4b_4_), depletion of Cu atoms from edges and corners (Figure 4b_5_), and the reduction of CuO_x_ species (Figure 4b_2,_b_5_).^[^ ^180^ ^]^ Similarly, another study using in situ EC‐AFM reveals nanoscale surface transformations of Cu (100) during eCO_2_RR in KHCO_3_ solution, with the morphology evolving from granular to smooth mound‐pit surfaces, depending on the applied potential. This study also highlights that undercoordinated copper sites increase with decreasing potential, illustrating how morphology, defect density, and potential interrelate.^[^ ^181^ ^]^ Additionally, Kely et al., applied in situ EC‐AFM to study a Cu‐Au catalyst for eCO_2_RR, identifying resistive CuO_x_ islands, passive adlayers, and variations in interfacial molecular ordering influenced by the electrolyte composition and demonstrating how local electrochemical environments affect interfacial charge transfer and catalysis.^[^ ^182^ ^]^ Nevertheless, the limited use of real‐time STM and AFM in eCO_2_RR studies stems from several difficulties, such as imaging instability during reactions, environmental noise, and probe sensitivity under harsh electrochemical conditions. To overcome these hurdles, improving the design of electrochemical cells to stabilize imaging environments is crucial. Enhancing probing materials and developing adaptive feedback systems can increase measurement precision, and integrating STM and AFM with complementary techniques like spectroscopy can offer a more comprehensive understanding of structural changes.

Scanning electrochemical microscopy (SECM) is another versatile technique that employs an ultramicroelectrode or nanoelectrode as a tip/probe to monitor in situ electrochemical reactions at various interfaces, such as liquid–liquid, solid‐liquid, and liquid‐gas.^[^ ^183^, ^184^ ^]^ The SECM tip/probe is precisely controlled by a motorized positioning system to approach or scan over the sample of interest, allowing for a detailed investigation of electrochemical processes.^[^ ^185^ ^]^

Koper et al., have conducted a series of studies to investigate eCO_2_RR using SECM. One study explored the role of metal cations in CO_2_ reduction in a 1 mm H_2_SO_4_ solution, revealing that metal cations are crucial for eCO_2_RR on Au, Ag, and Cu electrodes, as the reaction does not occur in their absence.^[^ ^186^ ^]^ The findings showed that partially desolvated cations stabilize the ^*^CO_2_^−^ intermediate through short‐range electrostatic interactions, redefining the eCO_2_RR mechanism. In another study, SECM was used to measure local pH variations during eCO_2_RR, providing insights into how the electrolyte's bulk pH and composition influence reaction activity and selectivity.^[^ ^187^ ^]^ Their results demonstrated that CO_2_ leads to the formation of a buffering HCO_3_^−^ layer, maintaining a stable pH at the interface compared to argon conditions. Simulations further accounted for diffusion dynamics, revealing local pH values were more acidic, providing valuable insights into local reaction conditions during eCO_2_RR. Additionally, Ye et al., developed a nano‐electrochemical cell on nanocrystal layers, enabling precise control of humidity, temperature, and gas environment.^[^ ^188^ ^]^ This cell models the gas‐electrolyte‐catalyst three‐phase boundary found in eCO_2_RR gas diffusion electrodes (Figure 4c_1_,c_2_). Using SECM, they isolated and studied the electrocatalytic behavior of individual Au nanocrystalline facets, as shown in Figure 4c_3_–c_5_. Their findings reveal that stepped Au (110) facets demonstrated higher activity and selectivity for eCO_2_RR compared to the (310) or (111) facets, particularly at low overpotentials where electrode kinetics dominate. This precise measurement of catalytic reactions through SECM provides critical insights into catalyst design and performance optimization. In addition, SECM, with its ultrahigh spatial resolution, can be effectively integrated with other analytical techniques for enhanced mechanism analysis. For instance, Bron et al., demonstrated the investigation of the structure‐activity relationship by coupling SECM with in situ Raman spectroscopy in eCO_2_RR.^[^ ^189^ ^]^ While in situ Raman microscopy monitored the structural changes of Cu_2_O microcrystals at low overpotentials, SECM in SG/TC mode detected the reaction products, revealing the potential‐dependent evolution of products and structural modifications. Similarly, SECM has been paired with atomic absorption spectroscopy to assess the performance of Cu electrodes in CO_2_‐saturated monoethanolamine solutions.^[^ ^190^ ^]^ This combination enables the detection of dynamic Cu_2+_ concentration changes during eCO_2_RR, while SECM imaging provides spatial distribution data for Cu^2+^ and pH near the electrode surface, contributing to a more thorough understanding of the reaction mechanism. Similarly, SECM faces challenges such as maintaining precise tip control over rough or dynamic surfaces and resolving fast reaction dynamics due to diffusion‐based signal collection. Integrating SECM with other techniques like Raman spectroscopy or atomic absorption spectroscopy requires balancing the spatial and temporal resolution, which can complicate data interpretation. Moving forward, advancements in nanoelectrode fabrication and adaptive control systems are essential to enhancing measurement precision. Coupling SECM with emerging in situ techniques and leveraging machine learning for data analysis can provide deeper insights into eCO_2_RR mechanisms and improve catalyst design under practical conditions.

### 3.2 In Situ X‐Ray Diffraction

**Figure 5:**
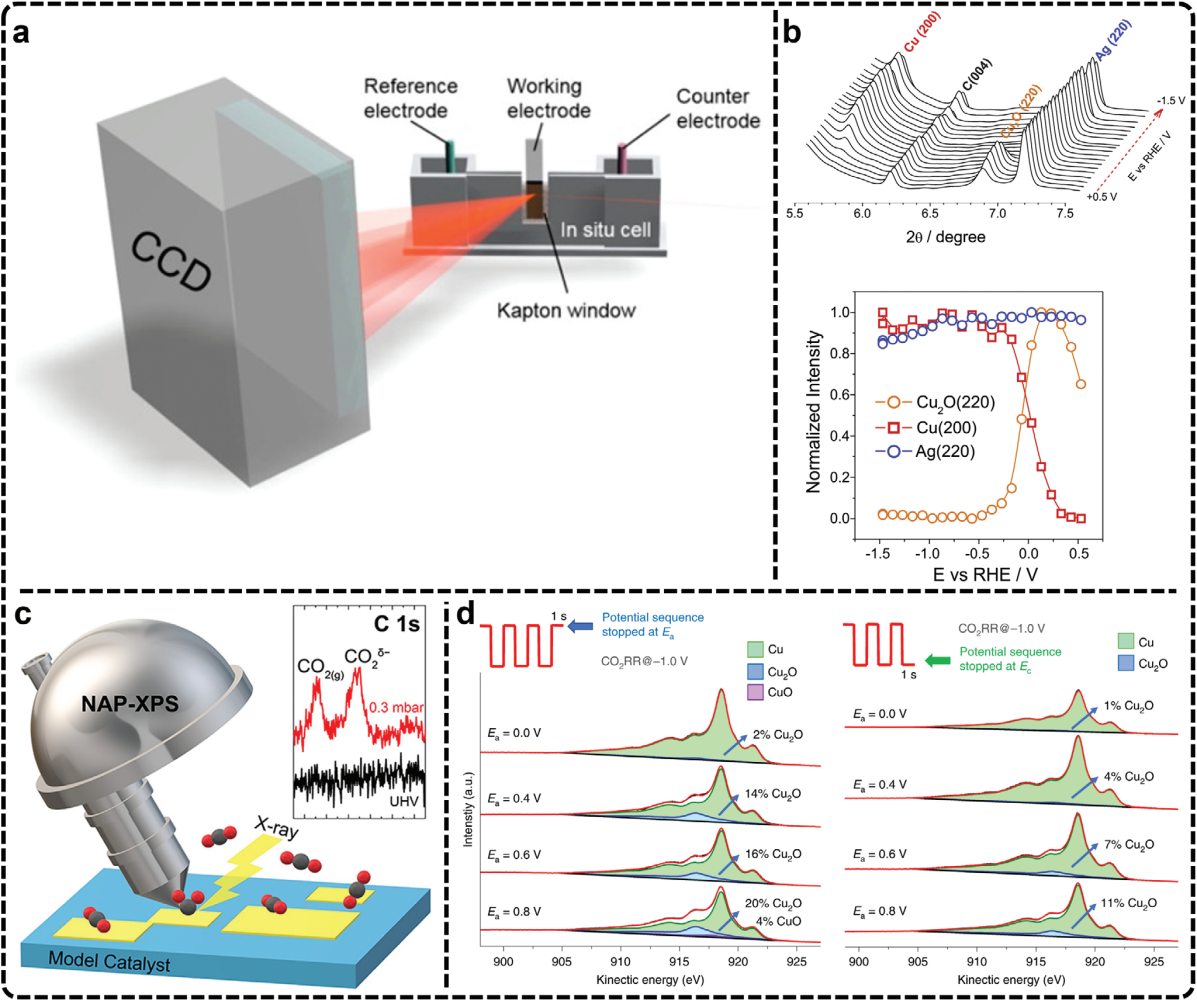
a) Schematic representation of an in situ grazing‐angle X‐ray scattering/diffraction apparatus applied to a liquid electrochemical cell. Adapted with permission.^[^ ^191^ ^]^ Copyright 2020, American Chemical Society. b) Operando XRD experiment demonstrating the electro‐reduction of Cu_2_O in the annealed Ag_15_Cu_85_ foam as a function of the applied electrode potential: the potential dependent XRD spectra and integrated and normalized intensities derived from spectra shown in panel (orange: integrated intensity of the Cu_2_O (220) peak; red: integrated intensity of the Cu (200) peak; blue: integrated intensity of the Ag (220) peak). Reproduced with permission.^[^ ^195^ ^]^ Copyright 2020, Elsevier. c) Schematic diagram of in situ XPS. Reproduced with permission.^[^ ^201^ ^]^ Copyright 2022, American Chemical Society. d) Quasi‐in situ XPS spectra of Cu^(100)^ electrode with the different pulse protocols. Adapted with permission.^[^ ^203^ ^]^ Copyright 2020, Springer Nature.

Although optical techniques excel at probing catalyst surfaces, their limitations in depth and surface penetration can be addressed by X‐ray techniques. In situ XRD leverages Bragg diffraction to obtain real‐time structural information about crystalline materials. A typical in situ XRD cell, shown schematically in **Figure** **5**a, enables monitoring of catalyst crystal phases and phase transitions, providing insights into bulk information of the catalyst.^[^ ^191^, ^192^, ^193^, ^194^ ^]^ Traditionally, in situ XRD has been extensively applied in battery research to investigate phase transitions under different voltage conditions. However, in the field of eCO2RR, where catalytic reactions primarily occur at the surface and often involve molecular or atomic catalysts without significant phase transition processes, the application of in situ XRD is relatively limited. Only a few research teams have utilized in situ XRD to analyze the phase transition patterns during catalysis. The study usually focuses on the dynamic oxidation and reduction behavior of Cu‐based oxides during eCO_2_RR.

Broekmann et al., utilized in situ XRD to examine the evolution of an AgCu foam catalyst during the eCO_2_RR to C_2_H_5_OH.^[^ ^195^ ^]^ Structural changes were observed under cathodic potentials relevant to eCO_2_RR, particularly involving the electro‐reduction of cupric and cuprous oxides (Figure 5b). The reduction of CuO to metallic Cu was found to proceed via crystalline Cu_2_O as an intermediate, with this transformation occurring before eCO_2_RR initiation. While the Ag components remained stable within the potential range relevant to Cu oxide reduction, though some Ag leaching was noted at more negative potentials. Additionally, compared to conventional XRD, grazing incidence X‐ray diffraction (GIXRD) is often more suitable for electrocatalysis studies as it provides insights into the near‐surface structure of catalysts. For instance, Chorkendorff et al., employed in situ GIXRD to demonstrate that the near surface of Cu_2_O on a polycrystalline Cu electrode was fully converted into metallic Cu under electrocatalytic CO reduction conditions, highlighting the potential of this technique for surface‐level structural analysis.^[^ ^196^ ^]^ Despite the advantages, in situ XRD faces notable challenges in eCO_2_RR. Its reliance on crystallinity limits application to molecular or atomically dispersed catalysts, which are common in this field. Additionally, the limited spatial resolution restricts the detection of local active sites and structural heterogeneity. Addressing these challenges requires the integration of in situ XRD with complementary techniques to achieve a more comprehensive understanding of catalytic processes. Further advancements, such as the development of synchrotron‐based XRD, methodologies for studying amorphous materials, and the standardization of experimental setups, hold promise in enhancing its utility and expanding its application to eCO_2_RR research.

### 3.3 In Situ X‐Ray Spectroscopy

X‐ray spectroscopy techniques are particularly useful for assessing catalyst stability and identifying the electronic state of active sites during reactions.^[^ ^197^, ^198^, ^199^, ^200^ ^]^ Key X‐ray techniques used for in situ characterization include X‐ray photoelectron spectroscopy (XPS) and XAS. XPS is widely employed to analyze changes in elemental composition and electronic structures by measuring the binding energy and chemical shifts of atomic electrons. However, XPS often requires ultra‐high vacuum conditions, which complicates its application. Recent advances in environmental pressure XPS have enabled real‐time monitoring of catalyst surface evolution under more practical conditions (Figure 5c), although challenges remain, such as mass transport limitations and potential radiation‐induced changes in electrolyte composition.^[^ ^175^, ^201^, ^202^ ^]^ Currently, most XPS in electrocatalysis rely on quasi‐in situ methods, which involve quickly transferring samples to the XPS instrument in a controlled environment post‐reaction.^[^ ^203^, ^204^, ^205^, ^206^ ^]^ This technique offers a snapshot closer to the real reaction state compared to traditional ex situ XPS.

In one study, quasi‐in situ XPS was combined with pulse electrocatalysis to examine the surface composition of an electropolished Cu^(100)^ electrode during eCO_2_RR (Figure 5d).^[^ ^203^ ^]^ A pulse sequence was applied with anodic potentials (E_a_) varied from 0 to 0.8 V and a constant cathodic potential (E_c_) of −1.0 V, to control the surface composition and assess the stability of different Cu^δ+^ species under reaction conditions. Cu LMM Auger electron spectra after alternating potential sequences revealed that only 4% of CuO formed at E_a_   =  0.8 V after a 1 s pulse, while Cu_2_O species (7–11%) persisted during the cathodic pulse, indicating the incomplete reduction of oxides during eCO_2_RR. In another quasi‐in situ XPS study, researchers found that oxygen‐containing species on the Cu catalyst surface were fully reduced during the reaction, yet selectivity toward C_2+_ products was maintained.^[^ ^78^, ^207^ ^]^ Although the findings from various studies differ and debate persists regarding the role of residual oxygen in promoting C─C coupling, they offer valuable insights into surface oxide behavior during eCO_2_RR. In short, quasi‐in situ XPS presents a valuable opportunity for understanding catalyst surface dynamics in eCO_2_RR, although it falls short of capturing real‐time surface changes. Currently, the primary challenge remains the ultra‐high vacuum conditions traditionally required for XPS, which limit its application under realistic reaction environments. Future efforts should focus on further improving environmental pressure XPS to address mass transport limitations and reduce artifacts.

**Figure 6:**
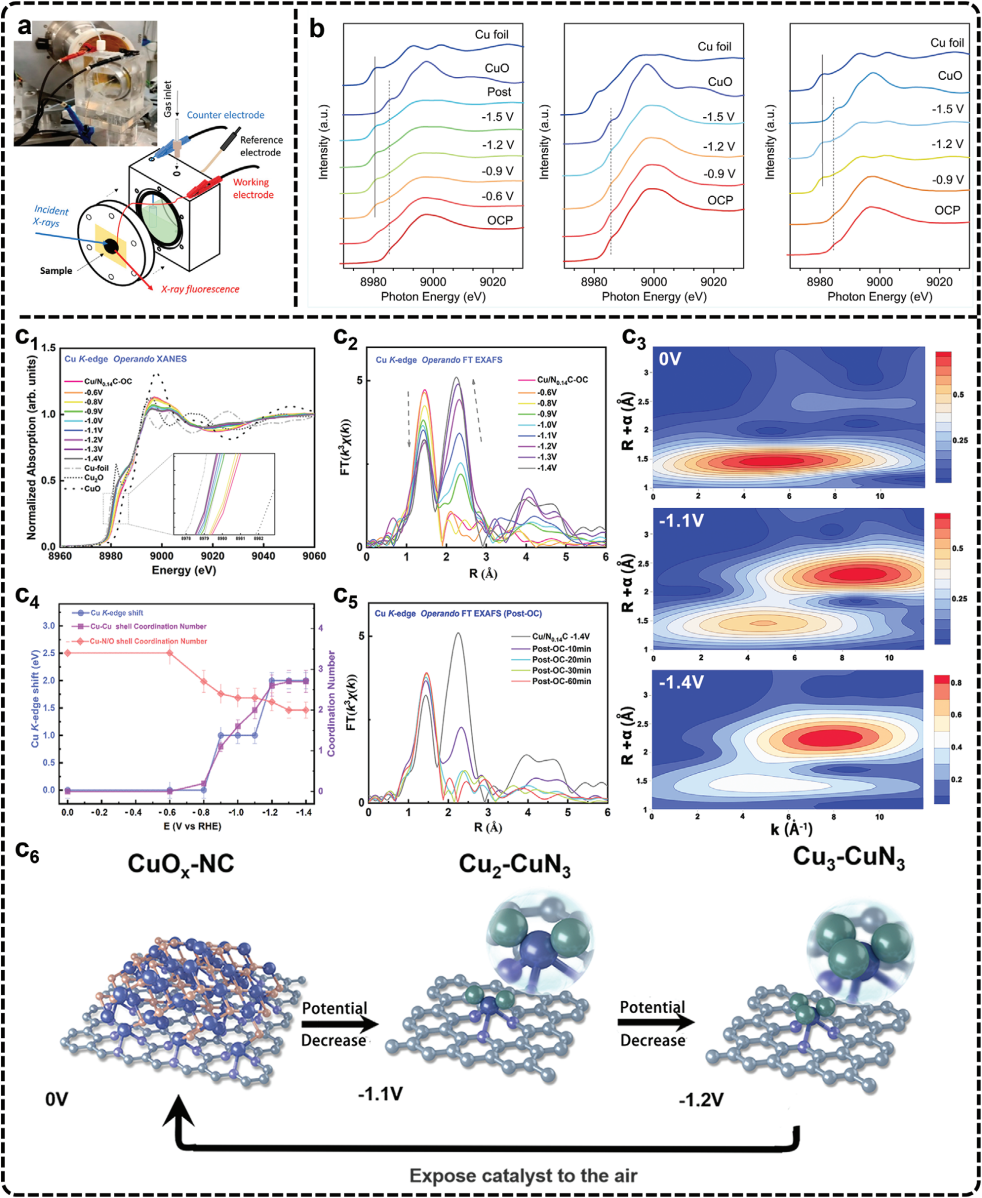
a) Large volume electrochemical cell for XAS measurements. Adapted under terms of the CC‐BY license.^[^ ^213^ ^]^ Copyright 2020, Timoshenko et al. b) In situ XAS under eCO_2_RR conditions of Cu K‐edge XANES spectra of Hex‐CuO, Hex‐Cu_2_O, and Oct‐Cu, respectively (from left to right). Adapted under terms of the CC‐BY license.^[^ ^216^ ^]^ Copyright 2022, Yang et al. c) In situ XAS characterization of Cu/N_0.14_C. Adapted under terms of the CC‐BY license.^[^ ^217^ ^]^ Copyright 2022, Su et al. c_1_) Cu K‐edge operando XANES of Cu/N_0.14_C from OC to −1.4 V versus RHE in 0.1 m KHCO_3_. c_2_) Operando FT‐EXAFS of Cu/N_0.14_C. c_3_) Wavelet transforms for the k^3^‐weighted Cu K‐edge EXAFS signals at OC, −1.1 V, and −1.4 V versus RHE during the eCO_2_RR. c_4_) The relationship of Cu/N_0.14_C absorption edge, CN of first shell, CN of second shell, and the potential. c_5_) Operando FT‐EXAFS of Cu/N_0.14_C after the applied potential was switched off and other conditions remained unchanged. c_6_) Proposed scheme for the reversible formation of the catalytically active Cu_n_–CuN_3_ cluster based on operando XAS analysis (rufous, O; gray, C; purple, N; blue, Cu bond to both N and Cu; green, Cu just bond to Cu).

In situ XAS, including techniques like X‐ray absorption near‐edge structure (XANES) and extended X‐ray absorption fine structure (EXAFS), is a critical tool for understanding the coordination environment and chemical states of catalytic materials during reactions.^[^ ^202^, ^208^, ^209^ ^]^ These methods provide insights into the dynamic evolution of active sites, tracking changes in vacancy types, local coordination, and electronic states under operating conditions.^[^ ^200^, ^210^, ^211^ ^]^ Hence, XAS has become indispensable for exploring structure‐function relationships in electrocatalysis.^[^ ^199^, ^212^ ^]^ In situ XAS utility in eCO_2_RR has been thoroughly reviewed, demonstrating its versatility in probing various catalytic systems.^[^ ^193^, ^213^ ^]^ For example, Yang et al., reviewed the utility of in situ XAS in studying single‐atom doped systems and metal complexes during eCO_2_RR, showing how shifts in coordination geometry and molecular structure impact product selectivity.^[^ ^193^ ^]^ To accommodate different applications for eCO_2_RR, several experimental cell designs have been developed to optimize in situ XAS measurements, each balancing trade‐offs related to the reaction environment.^[^ ^199^, ^214^, ^215^ ^]^ A common setup involves single‐compartment cells, where the sample is placed on the front panel of the cell facing both the electrolyte and X‐ray window (**Figure** **6**a).

In XAS, XANES is particularly useful for determining the chemical state of the catalyst, while EXAFS provides detailed information about the coordination structure of neighboring atoms. To track the oxidation state, Peng et al., used in situ XANES to investigate bicentric Cu complexes with varying spatial and coordination geometries (Hex‐2Cu‐O, Hex‐2Cu‐2O, and Oct‐2Cu), revealing Cu^2+^ reduction to Cu^0^ across all samples (Figure 6b).^[^ ^216^ ^]^ The Hex‐2Cu‐O complex, exhibiting the largest change in oxidation state, highlighted how complexes with greater intramolecular tension and asymmetry displayed reduced electrochemical stability but more active Cu centers. These Cu clusters, combined with partially reduced ligands, were particularly effective in converting CO_2_ to C_2_H_5_OH. Furthermore, Zheng et al., used in situ XANES to identify catalytic centers with double sulfur vacancies on CuS and showed that these sites enhanced ^*^CO binding and stabilized ^*^CO and ^*^COCO dimers, which were critical for improving C_2_ product formation.^[^ ^135^ ^]^ In another typical study, in situ XAS is used to track the dynamic structural evolution of CuO clusters supported on N‐doped carbon nanosheets during eCO_2_RR.^[^ ^217^ ^]^ The authors observed a potential‐dependent transformation from CuO to Cu_2_‐CuN_3_ clusters, which served as active sites for CO_2_ reduction. The study revealed critical shifts in the Cu valence state (Figure 6c_1_) and coordination geometry (Figure 6c_2_,c_3_), with a clear decrease in Cu─O/N coordination and the emergence of Cu─Cu bonds as the potential decreased (Figure 6c_4_). These structural changes were closely tied to the formation of C_2+_ products, such as C_2_H_5_OH. Notably, when the applied potential was removed, the Cu─Cu bonds disappeared, and Cu─O/N bonds reformed, confirming the reversible nature of these transformations (Figure 6c_5_,c_6_). Furthermore, Yang et al., employed high‐energy resolution fluorescence detection XAS to monitor the full lifecycle of Cu nanocatalysts, revealing that after 1 h of eCO_2_RR, the catalysts transformed into low‐coordinated metallic Cu nanoparticles, free from oxides and ligands.^[^ ^218^ ^]^ These nanoparticles were identified as the true active sites driving the eCO_2_RR process. These studies underscore the importance of in situ XAS in revealing the atomic‐scale transformations that govern selectivity and activity in eCO_2_RR, offering valuable insights into electrocatalytic processes. Despite the importance, there are challenges in achieving the spatial and temporal resolution required to capture rapid changes at active sites. Moreover, experimental setups must strike a balance between maintaining reaction environments and ensuring data acquisition. Advancing XAS capabilities will require the development of higher‐resolution detection methods to identify subtle shifts in electronic and coordination states. Expanding in situ XAS capabilities to accommodate complex catalytic systems will further enhance its impact on electrocatalysis.

### 3.4 In Situ Fourier Transform Infrared Spectroscopy

**Figure 7:**
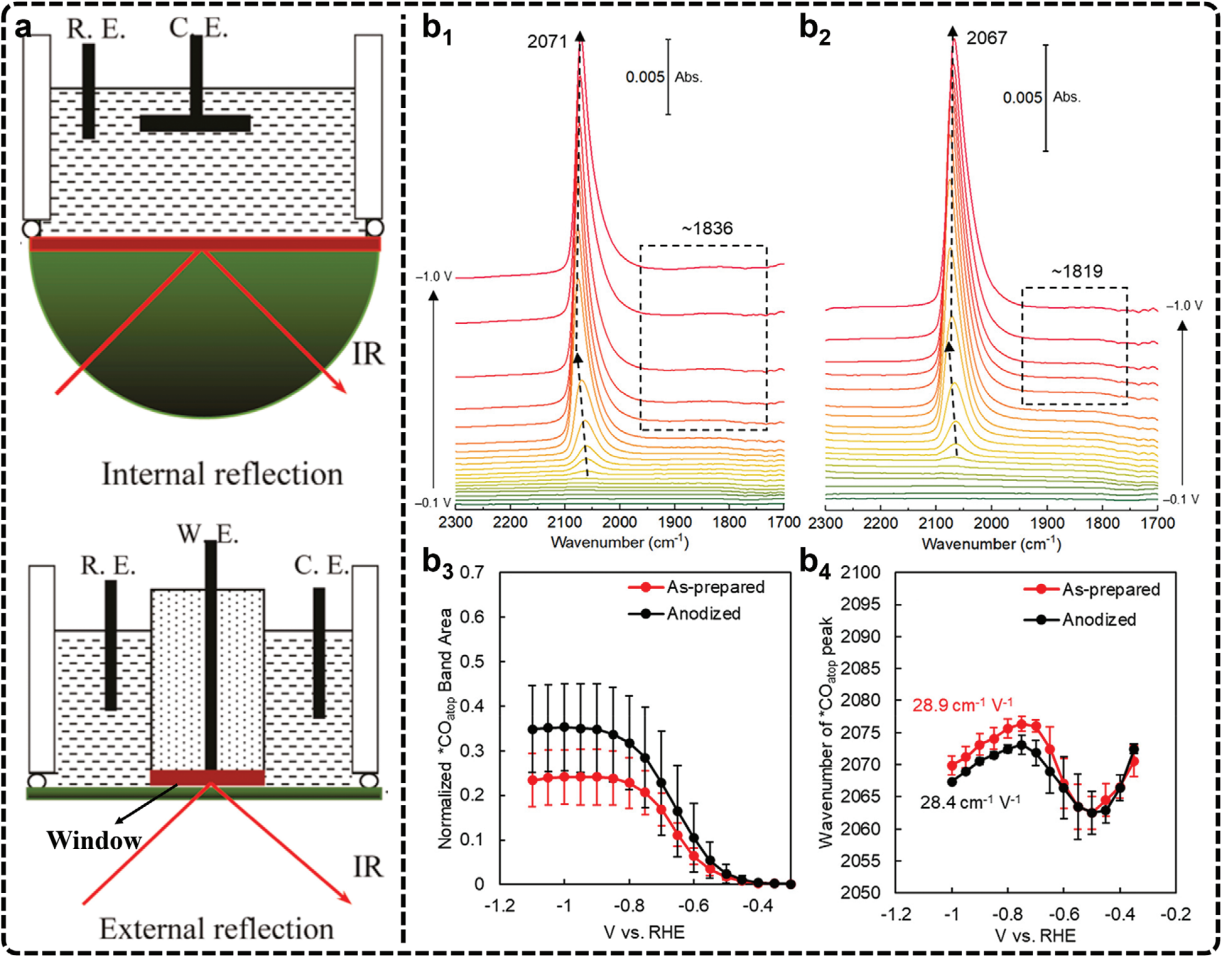
a) Schematic of ATR electrolysis cells. Adapted with permission.^[^ ^222^ ^]^ Copyright 2016, Elsevier. b) In situ SEIRAS spectra (1700–2300 cm^−1^ region) of the b_1_) as‐prepared copper thin film and b_2_) anodized copper thin film in a CO_2_‐saturated, 0.1 m KHCO_3_ electrolyte in H_2_O. The spectrum at 0 V was used as the background. b_3_) Normalized area of the ^*^CO band as a function of the cathodic potential (normalized vs the largest ν(O─H) band of ^*^H_2_O). b_4_) Vibrational frequency of the ^*^CO_atop_ band as a function of the cathodic potential. The ^*^CO band generally redshifts as stronger cathodic potentials are applied due to the vibrational Stark effect. As the *CO coverage sharply increases from −0.5 to −0.75 V, the dipole interactions between ^*^CO adsorbates induce a blueshift in the ^*^CO band position. The error bars reflect the standard deviations of three independently prepared Cu thin films. Adapted with permission.^[^ ^75^ ^]^ Copyright 2024, American Chemical Society.

In situ FTIR is one of the valuable tools to study eCO_2_RR because it combines electrochemical measurements with real‐time monitoring of catalytic reactions at gas‐liquid‐solid interfaces. This technique provides insights into the identification of intermediates, transient states, and product properties involved in electrode reactions.^[^ ^31^, ^219^ ^]^ It also reveals the orientation and bonding of adsorbed species on electrode surfaces, offering a molecular‐level understanding of electro‐driven mechanisms.^[^ ^220^ ^]^ However, eCO_2_RR typically occurs in aqueous electrolytes, and the presence of H_2_O can complicate the detection of intermediates. Water absorbs infrared light, especially at its bending (≈1640 cm^−1^) and stretching vibration peaks (≈3500 cm^−1^), masking the weaker absorption signals from reaction intermediates.^[^ ^221^ ^]^ To address this, many studies use attenuated total reflectance (ATR) electrolysis cells (**Figure** **7**a), which rely on the evanescent wave generated at the electrode surface to detect infrared signals of surface species.^[^ ^222^, ^223^ ^]^

ATR‐FTIR is particularly effective in monitoring adsorbates, especially species with C─O bonds, which have high infrared absorption cross‐sections.^[^ ^224^, ^225^, ^226^, ^227^ ^]^ Consequently, in the case of Cu‐based catalysts used for generating C_2+_ products, in situ FTIR has been reported in the literature to unravel potential reaction mechanisms. For example, in situ FTIR has consistently identified ^*^CO as a common and crucial intermediate in C─C coupling studies. Recently, Cheng et al., used in situ FTIR to study mesoporous CuO (m‐CuO) and cylindrical CuO (c‐CuO) during eCO_2_RR.^[^ ^57^ ^]^ The FTIR analysis revealed that m‐CuO promotes bridged adsorption of ^*^CO intermediates, leading to the formation of C_2_H_5_OH, while c‐CuO favors top adsorption, resulting in C_2_H_4_ production. This demonstrates how in situ FTIR can reveal the adsorption configurations of intermediates, effectively bridging the relationship between intermediates and product selectivity. In another example, a high‐performance CuO catalyst on N‐doped carbon nanosheets achieved a Faradaic efficiency (FE) of 73% for C_2+_ products, with C_2_H_5_OH selectivity reaching 51%.^[^ ^217^ ^]^ In situ FTIR revealed that Cu_2_‐CuN_3_ clusters display charge‐asymmetric sites, which intensify through ^*^CH_3_ adsorption, promoting the formation of C_2_H_5_OH.

Despite the significance, conventional FTIR spectroscopy often struggles with weak signals, leading to the loss of key intermediates and difficulty distinguishing hydrogen‐containing species. To address this, surface‐enhanced infrared absorption spectroscopy (SEIRAS) leverages the “surface enhancement effect” to increase signal intensity by up to two orders of magnitude. The enhancement arises from the plasmonic field near the metal surface, which restricts the effective signal range to within 10 nm of the surface, making SEIRAS particularly effective in detecting reaction intermediates during catalysis.^[^ ^228^, ^229^, ^230^ ^]^ Recently, Shao et al., utilized in situ SEIRAS to study the impact of Cu anodization on the adsorption of eCO_2_RR intermediates using metallic and mildly anodized copper thin films.^[^ ^75^ ^]^ Their results demonstrate that the pre‐oxidation of Cu significantly enhances CO_2_ reduction activity by improving CO_2_ activation, increasing ^*^CO uptake, and promoting C─C coupling. Specifically, for the ^*^CO adsorption region (Figure 7b_1_,b_2_), the in situ SEIRAS spectra of both films show a band at 2063–2077 cm^−1^, attributed to ^*^CO_atop_ stretching on copper sites, along with a weaker ^*^CO_bridge_ peak at 1819–1836 cm^−1^. The ^*^CO_atop_ band increases more rapidly in the anodized Cu film. When normalized to the O─H stretching band of adsorbed water, the integrated ^*^CO_atop_ peak areas show increased ^*^CO uptake in the anodized film (Figure 7b_3_). Additionally, a slight blueshift of 1.9 cm^−1^ suggests marginally weaker ^*^CO binding in the anodized film compared to the as‐prepared one (Figure 7b_4_). The results demonstrate that the pre‐oxidation of Cu significantly enhances CO_2_ reduction activity by improving CO_2_ activation, increasing ^*^CO uptake, and promoting C─C coupling. In another example, Zang et al., utilizing in situ ATR‐SEIRAS, clearly outlined the eCO_2_RR pathways on Cu4(MMI)4 and Cu8(MMI)4(tBuS)4 clusters (MMI = 2‐mercapto‐1‐methylimidazole) catalysts.^[^ ^231^ ^]^ Their study highlights how the coordination environment of Cu sites plays a critical role in determining the product distribution between C_2_ and C_1_ species.

Despite its advantages, in situ FTIR faces several limitations. One key challenge is its sensitivity, which requires enhancement, particularly when dealing with electrocatalysts that exhibit low reflectivity. Additionally, improving the time resolution is crucial to detecting short‐lived intermediates that play significant roles in the reaction dynamics. Emerging technologies, such as the use of infrared free‐electron lasers, have the potential to expand detection capabilities into the far‐infrared range. This can allow detailed studies of molecular adsorption bonds and surface lattice species, offering deeper insights into catalytic processes. Furthermore, integrating in situ FTIR with other advanced characterization techniques can provide a more holistic understanding of surface structure‐function relationships and the complex interfacial mechanisms in electrocatalysis, leading to better catalyst design and optimization.

### 3.5 In Situ Raman Scattering Spectroscopy

**Figure 8:**
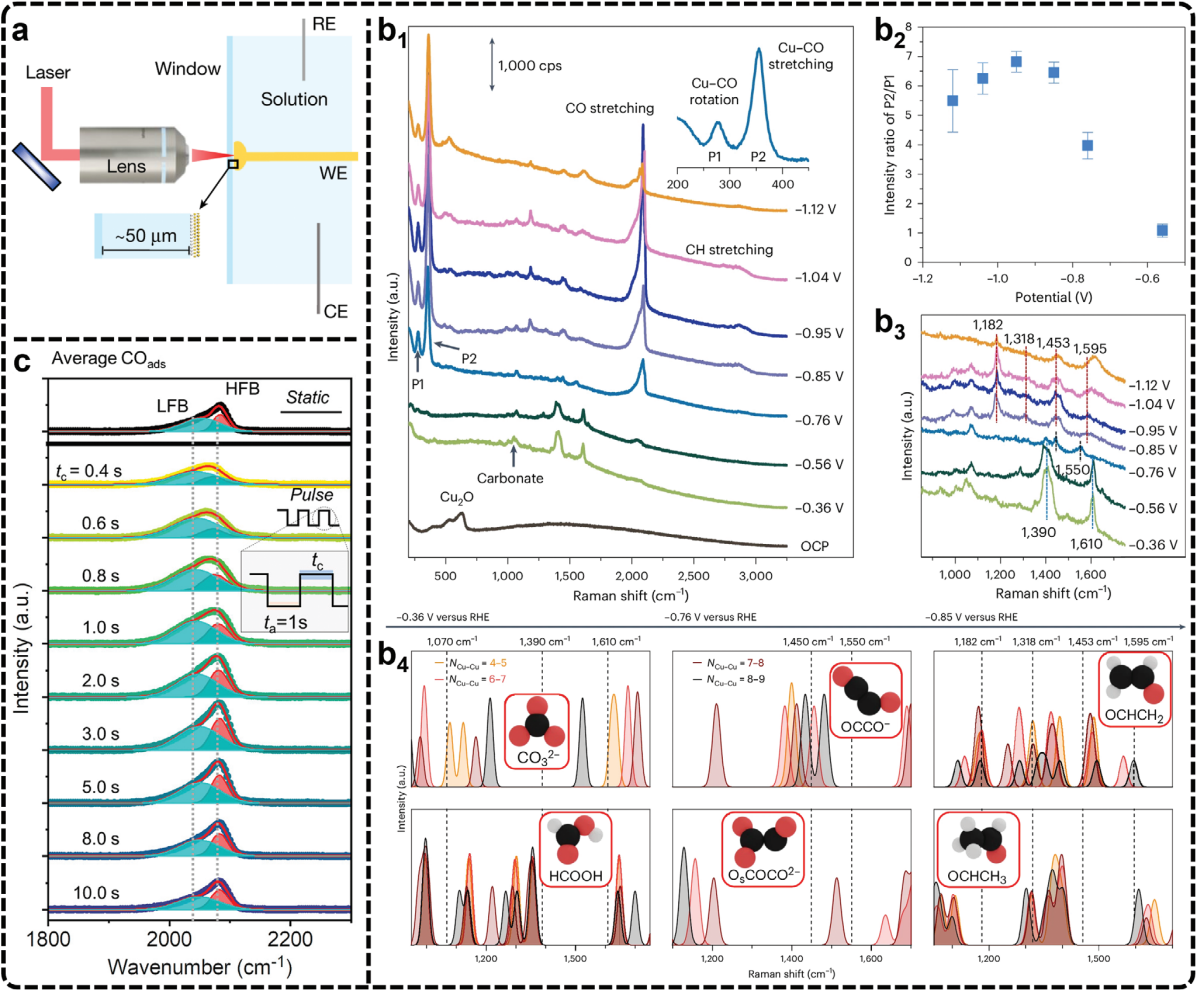
a) Schematic of the Raman experimental setup. CE, counter electrode; RE, reference electrode; WE, working electrode. Adapted with permission.^[^ ^237^ ^]^ Copyright 2021, Springer Nature. b) In situ Raman spectra and simulation. Adapted under terms of the CC‐BY license.^[^ ^240^ ^]^ Copyright 2024, Zhan et al. b_1_) Raman spectra of an electrochemically treated Cu foil acquired during eCO_2_RR for potentials ranging from theOCP to ≈−1.1 V_RHE_ in a CO_2_‐saturated 0.1 m NaClO_4_ electrolyte. cps, counts per second. b_2_) Potential‐dependent intensity ratio of P2 (Cu─CO stretching, ≈360 cm^−1^) to P1 (restricted rotation of adsorbed CO, ≈280 cm^−1^). b_3_) Zoom‐in Raman spectra in the region of 900–1700 cm^−1^ from ≈‐0.4 V_RHE_ to −1.1 V_RHE_, with relevant peaks highlighted via dashed lines. The error bars in b_2_ correspond to the s.d. of three independent measurements. Data are given as average ± s.d. b_4_) DFT vibrational frequencies for ^*^CO_3_^2−^, ^*^COCO^−^, OCHCH_2_ (top, left to right), ^*^HCOOH, ^*^O_s_COCO^2−^ and ^*^OCHCH_3_ (bottom, left to right) on adsorption sites with different coordination numbers, increasing from orange to black. Experimental signals at different applied potentials are indicated on top and highlighted in the panels by vertical dashed lines. Raman spectra were achieved by applying smearing of 10 cm^−1^ on each DFT frequency and overlapping the resulting peaks. Inset: different eCO_2_RR intermediates, with H atoms given in white, O atoms in red, and C atoms in black. c) Steady‐state in situ Raman spectra of the ^*^CO region on the Cu surface during potentiostatic and pulsed eCO_2_RR. Adapted with permission.^[^ ^246^ ^]^ Copyright 2023, American Chemical Society.

Raman scattering, complementary to infrared spectroscopy, detects changes in the vibrational or rotational states of a sample through inelastic light scattering caused by incident monochromatic light. In situ Raman scattering is also effective in identifying key intermediates, analyzing their configurations, determining reaction pathways, and assessing the impact of reaction conditions during electrocatalysis.^[^ ^232^, ^233^, ^234^, ^235^, ^236^ ^]^ Unlike infrared spectroscopy, Raman suffers less from significant interferences caused by water due to the small Raman scattering cross‐section, allowing for clearer detection of catalyst surface interactions, especially on oxidized catalysts. In typical Raman cells, a quartz window is placed above the catalyst surface to protect the lens and prevent laser damage (**Figure** **8**a).^[^ ^237^ ^]^ In eCO_2_RR, researchers have widely used electrochemical in situ Raman scattering to monitor real‐time reaction materials and intermediates evolution across varying potentials, making significant progress, as summarized by Tian et al.,^[^ ^234^ ^]^ For example, in situ Raman spectroscopy has been pivotal in identifying intermediates of ^*^COCO and ^*^CH_2_CHO on Cu (hkl) surfaces.^[^ ^238^ ^]^ Combining these spectroscopic observations with theoretical calculations has revealed that Cu (111) favors the formation of C_1_ products through ^*^COOH and ^*^CO formation, while Cu (110) promotes C_2_ products via pathways involving ^*^COCO and ^*^CH_2_CHO intermediates. These findings represent a major advancement in understanding eCO_2_RR mechanisms on various Cu facets. In a septate study, under eCO_2_RR‐relevant potentials, the Cu, Zn, and Cu‐Zn mixed catalysts are all found to experience rapid surface reduction within minutes as metal‐oxygen bonds gradually disappear.^[^ ^239^ ^]^ This quick reduction process reveals critical information about how oxide‐derived catalysts evolve under operational conditions, linking catalyst evolution to reactivity.

A more sensitive technique, known as surface‐enhanced Raman scattering (SERS), can significantly amplify the Raman signal using rough Au or Ag nanoparticles dispersed on the catalyst surface.^[^ ^237^ ^]^ Li et al., have utilized in situ SERS to track the configuration and dynamic behavior of water molecules at the interface between Pd single‐crystal electrodes and solution.^[^ ^237^ ^]^ Their findings provide valuable insights into the behavior of water molecules connected by hydrogen bonds versus those electrostatically interacting with cations at negatively charged electrodes. In eCO_2_RR, in situ SERS has also been widely utilized for monitoring catalyst evolution and identifying reaction intermediates. For example, in a dual‐active sites catalyst with atomic Cu sites and Cu nanoparticles supported on an N‐doped carbon matrix, in situ SERS revealed peaks related to the restricted rotation of adsorbed CO, Cu─CO stretching, and C─O stretching of atop ^*^CO.^[^ ^76^ ^]^ The findings suggest that a balanced ratio of atomic Cu and Cu nanoparticles facilitates CO_2_ conversion to C_2_ products. In contrast, the absence of significant CO peaks in single atomic Cu sites indicated poor CO_2_ reduction activity, emphasizing the importance of Cu nanoparticles in promoting the CO intermediate process for CO_2_‐to‐C_2_ conversion. Recently, Cuenya et al., have combined in situ SERS with density functional theory (DFT) calculations to elucidate the reaction mechanism of eCO_2_RR to C_2+_ products on Cu electrocatalysts.^[^ ^240^ ^]^ Their work reveals the reduction of the CuO_x_ phase to metallic Cu and tracks the evolution of key intermediates, including ^*^CO, ^*^HCOO^−^/^*^HCOOH, alongside changes in C─H, Cu─O, and Cu─C stretching bonds (Figure 8b_1_–b_3_). DFT calculations confirm the assignment of vibrational modes detected experimentally (Figure 8b_4_), providing insights into the postulated pathways leading to C_2_H_4_ and C_2_H_5_OH formation at specific active sites.

In some cases, capturing rapidly changing intermediates and surface species during eCO_2_RR can be challenging with typical modern Raman scattering spectroscopy, which often requires over 5 s to acquire high‐quality spectra. Time‐resolved Raman spectroscopy offers a more powerful alternative, enabling the study of Cu surfaces and reaction intermediates with sub‐second resolution.^[^ ^241^, ^242^ ^]^ This capability allows for the detection of fast‐evolving species at the catalyst interface during eCO_2_RR. For example, Weckhuysen et al., utilized time‐resolved surface‐enhanced Raman spectroscopy (TR‐SERS) to monitor intermediate dynamics and Cu surface evolution during eCO_2_RR.^[^ ^241^ ^]^ They observed that anodic treatment, followed by surface oxide reduction, led to rapid surface roughening and restructuring of the Cu surface within 7 s, resulting in a stable surface with increased local alkalinity. TR‐SERS also revealed that a dynamic ^*^CO intermediate was associated with C_2_H_4_ production, while a static ^*^CO species was linked to CO formation, illustrating how surface conditions influence product selectivity. Moreover, pulsed electrochemistry enhances selectivity for C_2+_ products compared to steady‐state electrocatalysis in eCO_2_RR by continuously evolving the catalyst.^[^ ^243^, ^244^, ^245^ ^]^ However, this approach introduces complexities related to the underlying mechanisms due to rapidly shifting potentials. Integrating in situ time‐resolved Raman spectroscopy with pulsed electrochemical methods shows significant potential in overcoming these challenges. Recently, in situ time‐resolved Raman spectroscopy with a resolution of 0.25 s has provided a real‐time observation of intermediates during each pulse cycle in pulsed eCO_2_RR, providing critical insights into the processes driving enhanced product selectivity.^[^ ^246^ ^]^ Under alternating anodic and cathodic potentials, the periodic oxidation and reduction of Cu lead to dynamic transitions between Cu_x_O and Cu_x_O/Cu mixtures on the catalyst surface (Figure 8c). These surface changes, mediated by the surface‐adsorbed ^*^CO‐controlled mechanism, can adjust the C_2+_/C_1_ product ratio during eCO_2_RR.

Overall, in situ Raman scattering is valuable for studying reaction intermediates and surface dynamics in eCO_2_RR, but challenges persist. The long spectrum acquisition time limits the ability to track rapid changes, while the use of nanoparticles like Au or Ag in SERS can affect the catalyst's natural activity. Moreover, distinguishing between similar C‐based intermediates is difficult due to overlapping spectral fingerprints, requiring advanced resolution techniques. Future efforts should prioritize improving temporal resolution and enhancing signal‐to‐noise ratios to track and identify intermediates more effectively under reaction conditions. Integrating Raman spectroscopy with complementary techniques like DFT calculations can further clarify reaction mechanisms, as DFT can predict Raman fingerprints and aid in intermediate identification. Furthermore, the combination of time‐resolved Raman spectroscopy with pulsed electrochemical methods holds great promise in understanding catalyst behavior and product selectivity under dynamic conditions.

### 3.6 Isotope Tracing

Isotope tracing is a crucial analytical technique widely used in electrocatalysis to investigate reaction mechanisms and active sites at a molecular level. By incorporating isotopically labeled atoms, such as ^13^C, ^18^O, or deuterium (D), researchers can track the movement of atoms during a catalytic reaction. This provides a clearer understanding of where atoms originate, how they interact with the catalyst surface, and how they evolve into products. In eCO_2_RR, isotope tracing is particularly useful for confirming the source of carbon or oxygen in the products, which can lead to improved electrolyzer and catalyst designs for higher selectivity and efficiency.^[^ ^247^, ^248^ ^]^ Furthermore, this technique can uncover active sites on catalysts, identify key intermediates, and clarify the reaction pathways, especially when combined with in situ spectroscopic techniques.^[^ ^75^, ^248^, ^249^, ^250^ ^]^ For example, Zhou et al., by utilizing ^13^CO_2_, H_2_^18^O, and D_2_O in in situ ATR‐SEIRAS, have accurately identified an uncommon adsorbed CO_2_ species at 1446 cm^−1^.^[^ ^227^ ^]^ They have further postulated that smaller cations like Li⁺ in electrolytes exhibit a higher capacity to stabilize adsorbed CO_2_ than larger cations and provided new information on how ion species influence the reaction mechanisms.

Ager et al., led several important studies using isotope tracing to uncover mechanisms in eCO_2_RR.^[^ ^144^, ^251^, ^252^ ^]^ In one investigation, they investigated the stability of residual oxides in oxide‐derived Cu (OD‐Cu) catalysts during eCO_2_RR using ^18^O isotope labeling.^[^ ^251^ ^]^ After synthesizing ^18^O‐enriched OD‐Cu catalysts and performing eCO_2_RR, ex situ SIMS revealed that less than 1% of the original ^18^O remained. This suggests that the residual oxides are unstable during eCO_2_RR. The study concludes that the high selectivity for C_2_ and C_3_ products is likely due to the high density of grain boundaries rather than stable oxides. ^13^C isotope labeling is another widely used technique to confirm the carbon source of products in eCO_2_RR.^[^ ^253^, ^254^, ^255^, ^256^ ^]^ One study using this technique, along with various analytical methods, found that the CO_2_ involved in eCO_2_RR primarily originates from the equilibrium between aqueous CO_2_ and bicarbonate in the electrolyte, rather than from CO_2_ gas diffusion.^[^ ^253^ ^]^ Electro‐kinetic analysis reveals a first‐order dependence on bicarbonate, suggesting that it enhances eCO_2_RR by increasing the effective CO_2_ concentration through rapid equilibrium exchange. Supporting this conclusion, another study using ATR‐SEIRAS and ^13^C isotopic labeling shows that on a Cu thin film, bicarbonate anions mediate CO_2_ transfer to the catalyst surface instead of direct CO_2_ adsorption.^[^ ^257^ ^]^ Several eCO_2_RR intermediates are identified, and the analysis reveals that surface‐bound ^*^CO originates from bicarbonate equilibrium, emphasizing the crucial role bicarbonate anions play in eCO_2_RR on Cu surfaces.

Isotope effects can also distinguish active sites by analyzing differences in isotopic product composition. For example, by reducing mixtures of ^13^CO and ^12^CO_2_, isotope labeling has shown that OD‐Cu catalysts have distinct active sites for producing specific products.^[^ ^252^ ^]^ One set of sites generates C_2_H_4_, another produces C_2_H_5_OH or CH_3_COOH, and a third forms 1‐propanol. The difference in ^*^COOH formation rates at each site results in unique isotopic product compositions. In contrast, polycrystalline Cu and Cu (100)/Cu (111) surfaces produce a mixture of C─C coupled products, lacking site‐specific selectivity. This suggests that tailoring specific active sites in OD‐Cu could improve product selectivity. In a recent study, Xu et al., used isotopic tracing with a mixture of ^13^CO and ^12^CO_2_ to investigate the carbon sources in CO_2_ reduction on dendritic Cu.^[^ ^250^ ^]^ By probing the surface coverages of adsorbed ^*12^CO and ^*13^CO through in situ SEIRAS and analyzing the isotopic distribution of products, they found significant deviations from the expected binomial distribution. This led to the identification of two distinct catalytic sites on the dendritic Cu surface: Cu_CO2_, which is more active in the initial CO_2_‐to‐CO conversion, and Cu_CO_, which favors further reduction of CO to C_2+_ products. Their two‐site model demonstrated that CO adsorbed on Cu_CO_ is at least six times more efficient in forming C_2+_ products than CO on Cu_CO2_. Additional experiments on Cu (111) and Cu (100) surfaces revealed that Cu_CO2_ sites are enriched on Cu (111), while Cu_CO_ likely involves defect sites on the Cu surface. These examples underscore how isotope tracing deepens our understanding of active sites and reaction mechanisms, providing a valuable tool for catalyst design.

In short, isotope tracing is a powerful technique to monitor the reaction mechanisms and active sites. Current challenges lie in the high cost of isotope‐labeled chemicals and the difficulty of distinguishing isotopic effects at closely related active sites, as these require highly sensitive techniques often limited by existing instrumentation. Overcoming these obstacles necessitates the development of advanced analytical tools and complementary methods to enhance both resolution and reliability. Combining isotope tracing with computational approaches, such as DFT, can bridge experimental findings with molecular‐level insights. Furthermore, leveraging isotope tracing to design catalysts with tailored active sites holds significant promise to improve the product selectivity and efficiency of eCO_2_RR.

## 4 Improving Selectivity: Intermediates Manipulation

To improve the selectivity of C_2+_ products in eCO_2_RR, the catalyst plays a critical role, as it is directly involved in the formation, stabilization, and conversion of key intermediates during the reaction. Among the metal catalysts investigated for catalyzing eCO_2_RR, Cu stands out as the primary metal, being capable of efficiently generating C_2+_ products such as C_2_H_4_, C_2_H_5_OH, and n‐propanol.^[^ ^96^ ^]^ While non‐Cu‐based catalysts have been explored, their selectivity and current density often fall short of expectations, underscoring the unique efficacy of Cu in this process, as summarized by Liu et al.^[^ ^3^ ^]^ This leads to the question of why Cu is the preferred catalyst for eCO_2_RR to C_2+_ product. The answer lies in the intricate interplay between the catalyst and the reaction intermediates. The activity and selectivity of a catalyst hinge on the strength of interaction with these intermediates. In the case of eCO_2_RR, Cu achieves an optimal balance of binding strengths for key intermediates, such as ^*^COOH, ^*^CO, and ^*^CHO species. These interactions are sufficiently strong to stabilize the intermediates on the catalyst surface yet not overly strong to impede further reaction steps and release, such as C─C coupling and protonation.^[^ ^258^ ^]^ Therefore, Cu‐based catalysts have become the focus of research efforts aimed at optimizing eCO_2_RR for C_2+_ product generation.

However, despite the effectiveness of Cu, a fundamental challenge persists in the slow reaction kinetics, particularly concerning C─C coupling, a crucial step for C_2+_ product formation.^[^ ^259^, ^260^, ^261^, ^262^, ^263^ ^]^ Theoretically, C─C coupling involves thermochemical steps that are independent of electron or proton transfer. Unlike electrocatalysis, thermal catalysis does not rely on catalyst surface polarization using external electrical energy, which poses a significant obstacle for conventional electrocatalytic approaches. To address this, researchers have focused on designing catalysts with active sites that lower the intrinsic energy barriers for C─C coupling by manipulating ^*^C_1_ intermediates. This involves enhancing the formation, stability, and reactivity of key intermediates while modulating their adsorption configurations to promote favorable reaction pathways. Accelerating C─C coupling is widely regarded as a result of effective intermediate regulation.^[^ ^79^, ^264^, ^265^, ^266^, ^267^, ^268^ ^]^ While computational predictions have provided valuable insights into the energetics of C─C coupling, experimental efforts are increasingly focused on exploring the interactions between intermediates and active sites.^[^ ^269^, ^270^, ^271^ ^]^ Recent advancements in in situ spectroscopy have furthered this understanding by allowing direct observation of reaction intermediates under operating conditions. The new knowledge has, in turn, guided catalyst design and spurred progress in this field. Specifically, promising strategies for intermediate manipulation involve enhancing the local concentration of active intermediates and regulating the types of ^*^C_1_ intermediates in the reaction. By modifying the composition and structure of the catalyst, researchers can increase the availability and reactivity of these intermediates to drive the C─C coupling process. For example, various catalyst designs have been shown to promote diverse C─C coupling modes (**Table** **1**), such as ^*^CO─^*^CO,^[^ ^272^, ^273^, ^274^, ^275^, ^276^, ^277^, ^278^, ^279^, ^280^, ^281^ ^] *^CO─^*^CHO,^[^ ^62^, ^63^, ^64^, ^65^, ^66^, ^67^, ^117^ ^] *^CO─^*^COH,^[^ ^68^, ^69^, ^70^, ^71^, ^72^, ^73^, ^74^, ^75^, ^116^ ^]^^*^CHO─^*^CHO,^[^ ^76^ ^] *^COH─^*^COH,^[^ ^77^ ^]^ and ^*^CH_x_─^*^CO,^[^ ^78^, ^79^ ^]^ etc.^[^ ^80^, ^81^, ^82^, ^83^, ^84^ ^]^ These modes offer significant potential for improving C_2+_ product selectivity.

**Table 1:** Summary of the properties of recently reported Cu‐based electrocatalysts for CO_2_ reduction into C_2+_ products.

Catalysts	Key Intermediates	Main Product	FE	*j* [mA cm^−2^]	Stability [h]	Cell Type	Electrolytes	Refs.
Cu + Cu_2_O + CuO	^*^CO─^*^CO	C_2_H_4_	48%	34	24	H	0.1 m KHCO_3_	[477]
Cu@AlO_x_ NCs	^*^CO─^*^CO	C_2_H4	22%	2.2	24	H	0.1 m KHCO_3_	[521]
Cu_2_O@SiO_2_─NH_2_	^*^CO─^*^CO	C_2_H_4_ C_2_H_5_OH	40.20% 29.00%	292	14	Flow	1 m KCl	[499]
Cu‐N_2_S_2_	^*^CO─^*^CO	C_2_H_4_ C_2_H_5_OH n‐C_3_H_7_OH	61.20% 19.75% 6.10%	12.4 4.01 1.24	7	H	0.1 m KHCO_3_	[278]
cAA‐CuNW	^*^CO─^*^CO	C_2_H_4_	60.70%	539	8	Flow	1 m KOH	[554]
Cd‐Cu	^*^CO─^*^CO	acetate	75%	150	/	MEA	3 m KOH	[555]
Ni SAC+Cu	^*^CO─^*^CO	C_2_H_4_	62%	370	14	Flow	1 m KHCO_3_	[371]
PTFE‐treated Cu	^*^CO─^*^CO	C_2_H_4_	67.30%	36.66	11	H	0.1 m CsI	[273]
Cu_2_O/CuO	^*^CO─^*^CO	C_2_H_4_	48.60%	400	30	Flow	1 m KHCO3	[155]
hydroxyl‐functionalized Cu_3_O	^*^CO─^*^CO	C_2_H_4_	50.50%	151	100	MEA	0.1 m KHCO_3_	[556]
multi‐shell Cu	^*^CO─^*^CO	C_2_H_4_	60%	900	10	Flow	1 m KOH	[464]
Cu(OH)_2_/BCS‐S‐derived Cu	^*^CO─^*^CO	C_2_H_4_	62.50%	22	7	H	1 m KCI	[557]
Ni/Cu‐NP@CMK	^*^CO─^*^CO	C_2_H_4_	72.30% 63%	406.1 200	15 30	Flow MEA	1 m KHCO_3_	[372]
Cu_0.9_Zn_0.1_	^*^CO─^*^CO	C_2_H_4_	73 ± 2%	150	100	Flow	acid (pH = 4)	[342]
MgAl‐LDH/Cu	^*^CO─^*^CO	C_2_H_4_	55.10%	300	7	Flow	1 m KHCO_3_	[417]
Cu_2_O/SiO_2_	^*^CO─^*^CO	C_2_H_4_ C_2_H_5_OH	48% 34%	24.5 17.5	6	Flow	0.1 m KHCO_3_	[424]
Ce‐Cu_2_O	^*^CO─^*^CO	C_2_H_4_	35%	36.14	7	Flow	0.5 m KHCO_3_	[277]
Pd‐Cu_2_O	^*^CO─^*^CO	C_2_H_4_	63.80%	24	35	H	0.5 m KHCO_3_	[558]
Cu–CeO_2_ nanorod	^*^CO─^*^CO	C_2_H_4_	43.20%	206.8	350	Flow	1 m KOH	[515]
Cu(I) and benzimidazole units	^*^CO─^*^CO	CH_3_COOH	61%	400	190	Flow	3 m KOH	[500]
Cu/PTFE	^*^CO─^*^CO	C_2_H_4_	49%	220	60	MEA	0.1 m KHCO_3_	[559]
Cu‐Pa	^*^CO─^*^CO	CH_3_COO^−^	70±5%	425	500	MEA	1 m KOH	[298]
Ag‐Cu(JNS‐100)	^*^CO─^*^CO	C_2_H_4_	54%	15.14	10	MEA	0.1 m KHCO_3_	[352]
Cu‐ZrO_2_	^*^CO─^*^CO	C_2_H_4_ C_2_H_5_OH	42% 36%	14 22.5	66	H	0.1 m KHCO_3_	[275]
CuO/Al_2_CuO_4_	^*^CO─^*^CO	C_2_H_4_	82.40%	20	100	H	0.1 m KHCO_3_	[364]
CuO/Ni SAs	^*^CO─^*^CO	C_2_H_4_	54.10%	660.5	3	Flow	1 m KOH	[369]
Cu/Ni‐NAC	^*^CO─^*^CO	C_2_H_4_	66%	100	10	Flow	1 m KOH	[370]
SrCuO_2_	^*^CO─^*^CO	C_2_H_4_	37.00%	200		PEEK	1 m KOH	[502]
Cu_2_O‐BN	^*^CO─^*^CO	C_2_H_4_	15%	13	14	H	0.5 m KHCO_3_	[512]
Cu‐Ag alloy	^*^CO_atop_─^*^CO_bridge_	C_2_H_4_	69.6 ± 1.3%	127.7 ± 30.3	40	MEA	0.1 m KHCO_3_	[344]
	^*^CO_atop_─^*^CO_atop_	C_2_H_5_OH	40.4 ± 2.4%	51.4 ± 0.1	20	MEA		
TS‐Cu polyhedron	^*^CO─^*^CHO	C_2_H_4_	72%	800	100	Flow	0.1 m KOH	[102]
bimetallic Cu_52_Ag_48_	^*^CO─^*^CHO	C_2_H_4_	69.20%	450	15	MEA	1 m KOH	[63]
Pd‐Cu_2_O	^*^CO─^*^CHO	C_2_H_4_	46.00%	33	/	H	0.1 m KCl + 0.1 m KHCO_3_	[62]
p‐Cu@m‐SiO_2_	^*^CO─^*^CHO	C_2_H_4_	56%	212	10	Flow	1 m KOH	[425]
CuOCu‐N_4_	^*^CO─^*^CHO	C_2_H_5_OH	56.30%	720 ± 34	0.25	Flow	1 m KOH	[320]
CuBr–4PP	^*^CO─^*^CHO	C_3_H_7_OH	10%	/	/	H	0.5 m KHCO_3_	[82]
mesoporous CuO nanofibers	^*^CO─^*^CHO	C_2_H_4_	49%	150	5	Flow	0.1 m KOH	[560]
Cavity Cu_2_O	^*^CO─^*^CHO	C_2_H_4_	41%	605 ± 14	12	Flow	1 m KOH	[561]
Cu_3_(PO_4_)_2_	^*^CO─^*^CHO	C_2_H_4_	69.70%	23	18	H	0.1 m KHCO_3_	[481]
AgCu single‐atom	^*^CO─^*^CHO	C_2_H_4_	48%	≈720	12	Flow	1 m KOH	[347]
N‐doped CuO	^*^CO─^*^CHO	C_2_H_4_	56%	34.6	22	H	1 m KHCO_3_	[319]
Cu(OH)BTA	^*^CO─^*^CHO	C_2_H_4_	57%	500	67	MEA	1 m KOH	[64]
Cu/P_2_O_7_	^*^CO─^*^CHO	C_2_H_4_	39.80%	350	30	MEA	0.1 m KOH	[308]
Cu_3_(HBtz)_3_(Btz)Cl_2_	^*^CO─^*^CHO	C_2_H_4_ C_2_H_5_OH	44% 21%	7.9	20	H	0.1 m KHCO_3_	[436]
Cu_3_N‐Ag NCs	^*^CO─^*^CHO	C_2_H_4_	35.00%	26.7	/	Flow	1 m KOH	[104]
Cu_2_O@Cu−TCPP(Co)	^*^CO─^*^COH	C_2_H_4_	54 ± 2%	500	20	Flow	1 m KCl	[71]
L‐Cu_x_O‐HC	^*^CO─^*^COH	C_2_H_4_	55%	≈600	12	Flow	1 m KOH	[562]
TA‐Cu	^*^CO─^*^COH	C_2_H_4_	63.60%	497.4	10	Flow	2 m KOH	[430]
Cu‐MOF	^*^CO─^*^COH	C_2_H_4_	65.90%	300	/	Flow	1 m KOH	[388]
Cu_9_Zn_1_/Cu_0.8_Zn_0.2_Al_2_O_4_	^*^CO─^*^COH	C_2_H_4_ C_2_H_5_OH	36% 36%	400	10	Flow	2 m KOH	[418]
TWN‐Cu_13.35_‐600‐SACs	^*^CO─^*^COH	C_2_H_5_OH	81.90%	35.6	26	H	0.5 m CsHCO_3_	[274]
CuAl‐LDH	^*^CO─^*^COH	C_2_H_4_	50%	22	11	H	0.1 m KHCO_3_	[419]
AEI‐OD‐Cu	^*^CO─^*^COH	C_2_H_4_	65%	800	9	Flow	1 m KOH	[563]
CuOD‐Cu	^*^COH─^*^COH	n‐C_3_H_7_OH	≈17.9%	50	150	H	0.1 m KHCO_3_	[134]
Cu_1_Ni‐BDP	^*^COH─^*^COH	C_2_H_4_	52.70%	530	25	Flow	0.1 m KOH	[77]
PcNi‐DMTP	^*^COH─^*^COH	CH_3_COOH	51.20%	410	200	MEA	1 m KOH	[564]
CuAg_5%_N_20h_	^*^CO─CH_3_CHO	C_3_H_7_OH	39%	150	9	Flow	1 m KOH	[565]
Cu‐Hist	^*^CO─^*^CH_2_O	C_2_H_4_	32%	5	48	H	1 m KHCO_3_	[65]
CuSn‐HAB	^*^CO─^*^OCH_2_	C_2_H_5_OH	56%	68	35	Flow	1 m KOH	[80]
C_18_S–CuNP	^*^COOH─^*^CH_2_	CH_3_COOH	70%	100	100	GDE	0.1 m KHCO_3_	[84]
Cu‐NN	^*^CH_2_CH	C_2_H_4_	83 ± 2%	212	120	MEA	0.5 m KHCO_3_	[94]
Cu/Fe_3_O_4_‐LDH	^*^CH_2_CHO	C_2_H_5_OH	50.90%	1.5	10	H	0.5 m KHCO_3_	[421]
Au_0.02_Cu_0.98_	^*^CHCH_3_	n‐C_3_H_7_OH	18.2 ± 0.3%	16.6	6	Flow	1 m KOH	[81]
Cu_2_‐Mg	^*^CHCHO	C_2_H_5_OH	76.2 ± 4.8%	−600	/	Flow	1 m KOH	[359]
Cu_2_O‐NS	^*^CHCOH	C_2_H_5_OH C_2_H_4_	31% 40%	−100	10	Flow	0.1 m KOH	[312]
CoCu single‐atom alloy	^*^CHCOH	C_2_H_4_	15.56%	282	/	Flow	1 m KHCO_3_	[362]
CuAl_2_O_4_/CuO	^*^CHCOH	C_2_H_5_OH	41.00%	200	150	Flow	1 m KOH	[112]
Cu_2_O/Ag NCs	^*^CHCOH	C_2_H_5_OH	40.80%	326.4	6	MEA	1 m KOH	[333]
Inz‐Cu_3_	^*^CHOHCH_2_ ^*^CHOHCH_3_	C_2_H_4_ C_2_H_5_OH	35.27% 31.52%	320	/	Flow	Acid electrolyte	[566]
Cu/BaO_x_	^*^HCCHOH	C_2_H_5_OH	60%	400	20	Flow	1 m KOH	[427]
Cu_3_(BTC)_2_ (MOFs)	^*^HOCCH	C_2_H_5_OH	46%	166	50	Flow	1 m KOH	[70]
AgI‐CuO	^*^OCHCH_2_	C_2_H_4_	45%	48	6	H	0.5 m KHCO_3_	[490]
B–Cu_2_O	^*^HCOOH	C_2_H_4_	26.13%	24	8	H	0.5 m KHCO_3_	[511]
Gd_1_/CuO_x_	O^*^CCO	C_2_H_4_	40%	443	40	Flow	2 m KOH	[361]

This section reviews optimization strategies for Cu‐based catalysts in eCO_2_RR, focusing on approaches to regulate the surface density of ^*^CO intermediates and local proton donors to promote C─C coupling. Initial enrichment of ^*^CO and subsequent protonation of intermediates are critical, as they determine the reaction pathway and directly influence C─C coupling efficiency. Through an analysis of recent developments, we aim to illuminate the key factors driving Cu‐based catalysts in eCO_2_RR, laying the groundwork for future advancements in this field.

### 4.1 Increasing Surface ^*^CO Coverage

The formation of C_2+_ products in eCO_2_RR involves complex multi‐step electron/proton coupling processes. Among them, ^*^CO adsorption is an initial and essential step dictating the eCO_2_RR process. Not only does ^*^CO serve as a direct participant in well‐established coupling pathways such as ^*^CO─^*^CO, ^*^CO─^*^CHO, and ^*^CO─^*^COH to produce C_2+_ products, but it also acts as a precursor to other crucial intermediates like ^*^CHO, ^*^COH, and ^*^CH_x_ formed after subsequent protonation steps. As a result, researchers widely acknowledge that increasing the coverage of ^*^CO is a key strategy to enhance the selectivity of common C_2+_ products like alkenes and alcohols.^[^ ^105^, ^107^, ^125^, ^161^, ^260^, ^282^, ^283^, ^284^, ^285^, ^286^, ^287^ ^]^ Moreover, higher ^*^CO coverage has been shown to reduce the energy barrier of ^*^CO dimerization and suppress H_2_ formation.^[^ ^288^, ^289^ ^]^ Indeed, studies have revealed a significant correlation between the partial current density of C_2_H_4_ formation and the square of ^*^CO coverage,^[^ ^290^ ^]^ as well as a volcano‐shaped relationship between C_2_H_4_ selectivity and the ratio of atop‐bound ^*^CO to bridge‐bound ^*^CO.^[^ ^291^ ^]^ Additionally, a kinetic linear scaling relationship has been identified between intermediate adsorption and activation energy.^[^ ^292^ ^]^ These findings reveal the importance of surface ^*^CO distribution and the strength of its interaction with the catalyst. Theoretically, changes in ^*^CO coverage arise from alterations in ^*^CO binding strength. Stronger ^*^CO adsorption stabilizes intermediates and increases ^*^CO coverage, while overly strong binding risks catalyst poisoning. Conversely, weak binding can lead to premature desorption, consequently reducing coverage and inhibiting C─C coupling. Achieving an optimal balance in ^*^CO binding strength is, therefore, essential to improving ^*^CO coverage and C_2+_ product selectivity without compromising the catalytic activity. Experimental techniques, such as in situ FTIR and Raman spectroscopy, are commonly employed to monitor ^*^CO coverage, while computational tools like DFT calculations offer insights into how catalyst modifications influence ^*^CO adsorption strength. These complementary approaches have significantly advanced our understanding of how to optimize catalytic surfaces for enhanced performance. Over the past decade, various strategies for catalyst modification have been developed to enhance ^*^CO coverage.^[^ ^136^, ^283^, ^293^, ^294^, ^295^, ^296^, ^297^, ^298^, ^299^, ^300^, ^301^, ^302^, ^303^ ^]^ However, challenges persist, and research on mechanism elucidation and catalyst design continues to evolve. In this subsection, we review three effective and recently reported Cu‐based catalyst design strategies: coordination environment regulation, hetero metal incorporation, and hetero component modification. These approaches have been widely explored to increase surface ^*^CO coverage, thereby promoting C─C coupling.

#### 4.1.1 Coordination Environment Regulation

The coordination environment is a critical factor in determining reaction pathways and product distribution, as it alters the geometric and electronic structure of the metal catalytic center. This is especially significant for Cu‐based catalysts. By strategically designing the coordination environment, researchers can improve the adsorption of ^*^CO intermediates to enhance the ^*^CO coverage. Recent studies have focused on creating low‐coordinated Cu sites in both metallic Cu and Cu‐based oxide catalysts, as well as exploring non‐metal atom‐coordinated Cu materials to further optimize performance.

**Figure 9:**
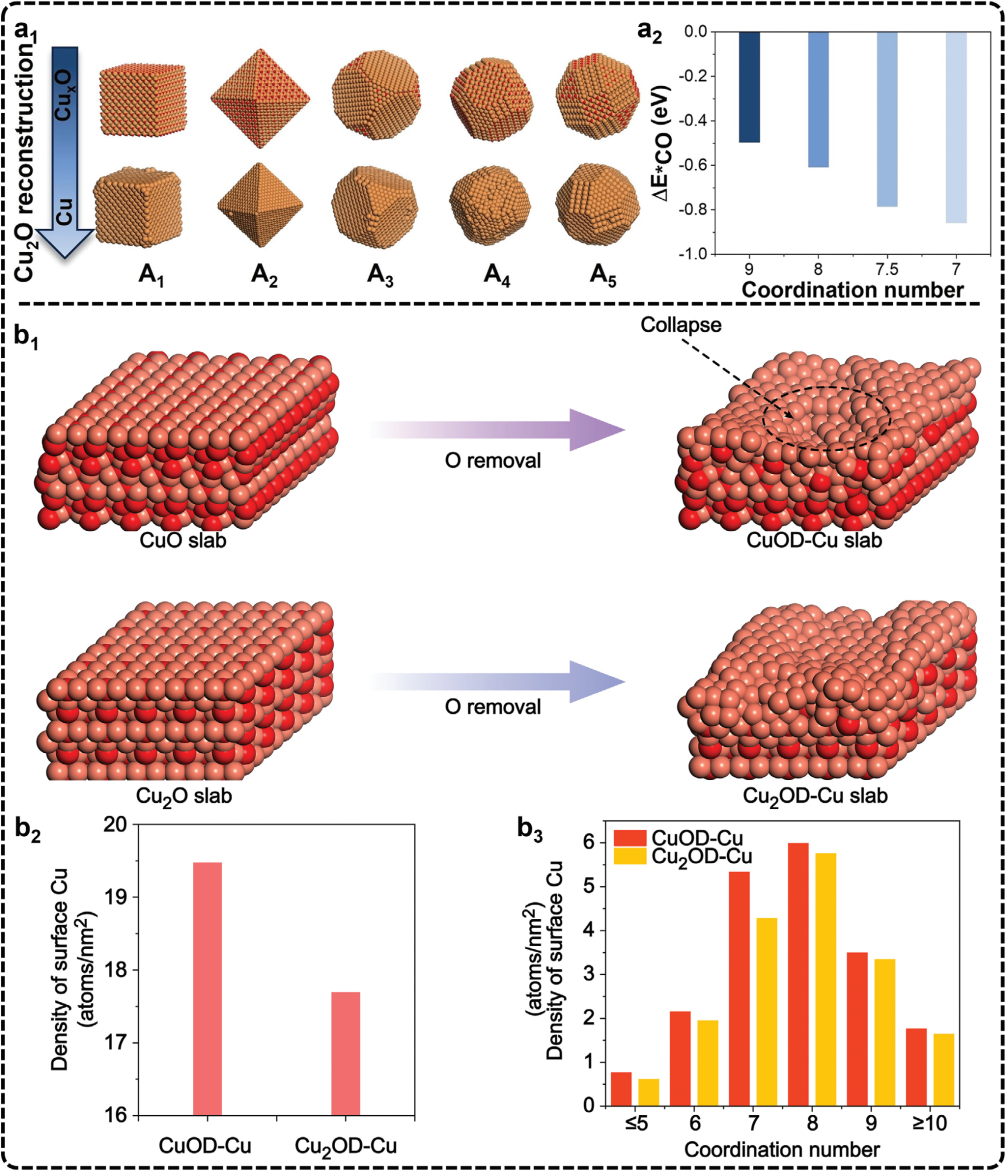
a) Cu_x_O reconstruction toward metallic Cu via oxygen removal through MD simulation a_1_) and ^*^CO adsorption changes with the coordination number (CN) of the Cu sites a_2_). Adapted with permission.^[^ ^102^ ^]^ Copyright 2024, John Wiley and Sons. b) Neural network potential‐based molecular dynamics simulation of the reconstruction of OD‐Cu, including b_1_) simulated initial and final configurations for oxygen removal reconstruction from CuO to CuOD‐Cu and from Cu_2_O to Cu_2_OD‐Cu, b_2_) statistics for surface Cu atoms and corresponding surface density on CuOD‐Cu and Cu_2_OD‐Cu, and b_3_) coordination number dependent surface Cu density on CuOD‐Cu and Cu_2_OD‐Cu. Adapted under terms of the CC‐BY‐NC license with permission from the American Association for the Advancement of Science.^[^ ^134^ ^]^ Copyright 2023, Long et al.

Low‐coordinated Cu sites in Cu‐based catalysts play pivotal roles in promoting ^*^CO adsorption, which in turn promotes C─C coupling to produce C_2+_ products.^[^ ^68^, ^78^, ^141^, ^304^, ^305^, ^306^, ^307^, ^308^ ^]^ These sites are often located at the surface or edges of the material, where they are less connected to surrounding metal atoms. This increased exposure allows these Cu atoms to interact more readily with external species like CO_2_, as they have more accessible electron orbitals to engage with the electron cloud of CO_2_. In addition, the reduced coordination alters the electronic structure of Cu, increasing the local density of states at the surface and enhancing the number of d‐orbital electrons available for interaction with ^*^CO intermediates. As a result, these sites exhibit stronger adsorption energy and more efficient charge transfer, facilitating the ^*^CO intermediates coverage. Studies have shown that polyhedral Cu catalysts with abundant low‐coordinated sites are more active in generating C_2+_ products, though the underlying mechanisms were initially unclear.^[^ ^309^, ^310^, ^311^ ^]^ Recent work combining advanced experimental characterizations and DFT calculations has provided insights into how these sites enhance ^*^CO adsorption to promote C─C coupling. For example, Liang et al., have computationally investigated ^*^CO adsorption energies on Cu sites with varying coordination numbers, demonstrating that low‐coordinated Cu sites exhibit stronger ^*^CO adsorption.^[^ ^68^ ^]^ They synthesized fragmented Cu with abundant low‐coordinated sites, which significantly improved FE toward C_2+_ products due to the improved ^*^CO coverage. Recently, the influence of coordination number on ^*^CO adsorption has also been extended to Cu‐based oxide and their derivatives. Xia et al., have synthesized a series of Cu_2_O polyhedra with different crystal facets and found that catalysts with more low‐coordinated Cu sites exhibited higher reactivity for C_2_H_4_ production.^[^ ^102^ ^]^ During eCO_2_RR, Cu_2_O polyhedrons transformed into metallic Cu, with a decrease in the coordination number corresponding to the number of facets (**Figure** **9**a_1_). The electrochemical assessment indicates that the catalyst rich in low‐coordinated Cu is more effective in generating C_2_H_4_, establishing a link between coordination number and product selectivity. Theoretical calculations further suggest that a lower coordination number increases ^*^CO adsorption energy to promote ^*^CO─CO coupling (Figure 9a_2_), emphasizing the importance of constructing low‐coordinated Cu sites. Similarly, ultrathin 2D Cu_2_O nanosheet catalysts with coordination defects and oxygen vacancies have demonstrated optimized ^*^CO adsorption and coverage, leading to significantly enhanced C_2+_ product selectivity, as revealed by in situ spectroscopy and theoretical calculations.^[^ ^312^ ^]^ Furthermore, a recent study by Sargent et al., reveals a hybrid Cu catalyst with a planar‐Cu and a coordinatively unsaturated dual‐Cu site. The materials provide distinct active sites that enrich ^*^CO coverage and facilitate CO‐to‐C_2+_ conversion under acidic conditions, thereby emphasizing the potential of unsaturated dual‐Cu sites for applications in acidic environments.^[^ ^115^ ^]^

Beyond ingenious morphology design, in situ reconstruction of Cu‐based oxide catalysts has been shown to expose low‐coordinated sites. Tang et al., found that CuO‐derived Cu intrinsically possesses a higher density of undercoordinated sites and a larger surface Cu atomic density than Cu_2_O‐derived Cu (Figure 9b).^[^ ^134^ ^]^ Further investigation indicated that these reconstructed structures show great potential for increasing ^*^CO coverage, rationalizing the improved C_2+_ selectivity. Zhang et al., employed an in situ electrochemical reconstruction strategy to create low‐coordinated Cu sites at the Cu/Cu_2_O interface of a Cu/Cu_2_O catalyst.^[^ ^141^ ^]^ These low‐coordinated sites improve ^*^CO coverage and enhance the adsorption of ^*^CH_2_CO and ^*^CH_2_CHO, promoting the formation of C_2_H_5_OH. In addition to Cu oxides, N‐doped CuO_x_ undergoes in situ reconstruction during eCO_2_RR, forming lattice‐strain‐stabilized N‐doped Cu with abundant defect sites. These low‐coordination sites enhance ^*^CO coverage, facilitating the generation of n‐propanol. Together, these studies confirm that low‐coordinated Cu sites, whether on metallic Cu or Cu oxides, significantly enhance ^*^CO adsorption. This establishes a crucial link between site construction and the manipulation of reaction intermediates, highlighting the importance of catalyst design in improving reaction outcomes.

**Figure 10:**
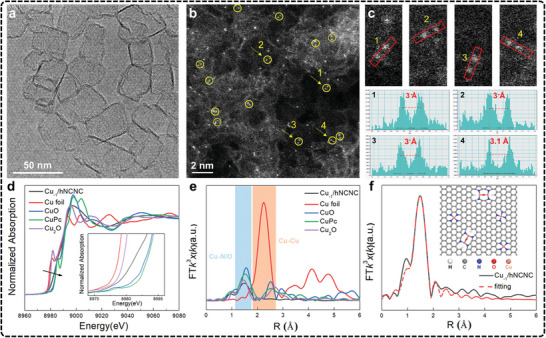
Morphology and structure characterizations of Cu_‐1_/hNCNC. Reproduced with permission.^[^ ^320^ ^]^ Copyright 2024, American Chemical Society. a) TEM image. b,c) HAADF‐STEM image showing twinned Cu atoms (circled) and isolated Cu atoms b) and corresponding enlargement of the sites 1–4 and intensity profiles c). d) Cu K‐edge XANES spectra of Cu_‐1_/hNCNC and the references. e) FT‐EXAFS spectra of Cu_‐1_/hNCNC and the references. f) FT‐EXAFS fitting curve of the Cu K‐edge in R‐space for Cu_‐1_/hNCNC and the corresponding structure diagram.

In addition to low‐coordinated Cu atoms in metallic and oxide catalysts, researchers have increasingly explored the incorporation of non‐metal elements to optimize the coordination environment and improve ^*^CO coverage. Non‐metal atoms or molecules, such as C and N, have shown effectiveness in functionalizing Cu sites by altering the local environment and influencing the interaction between active sites and key intermediates.^[^ ^160^, ^274^, ^278^, ^313^, ^314^, ^315^, ^316^, ^317^, ^318^, ^319^ ^]^ For instance, Hu et al., have designed a Cu‐based catalyst (Cu_–1_/hNCNC) featuring oxygen‐bridged Cu binuclear sites (CuOCu‐N_4_) alongside conventional Cu 1N_4_ mononuclear sites on hierarchical nitrogen‐doped carbon nanocages.^[^ ^320^ ^]^ HAADF‐STEM images (**Figure** **10**a–c) showed both twinned Cu atoms (circled) and isolated Cu atoms, indicating different coordination environments. XAS analysis (Figure 10d–f) further reveals the complex electronic state and low coordination number of Cu sites (3.3 ± 0.1), confirming the dual‐site structure. Electrochemical evaluation and DFT calculations demonstrate that the Cu 1N_4_ mononuclear sites generate CO, which is subsequently utilized by the adjacent CuOCu‐N_4_ sites, thereby increasing the local ^*^CO concentration and improving ^*^CO─^*^CHO coupling efficiency. This approach illustrates the value of creating varied Cu coordination environments to enable tandem catalysis. Similarly, the incorporation of other non‐metal heteroatoms, such as N, P, S, and O, to modify the Cu coordination environment has been studied by Qiao et al., who systematically explored surface ^*^CO coverage and material evolution in these Cu‐based compounds.^[^ ^321^ ^]^ Their findings underscore the critical role of nitrogen‐engineered Cu (N─Cu) catalysts in promoting ^*^CO adsorption on both bridge and atop Cu sites, enhancing C─C coupling and increasing C_2+_ product formation.

Cu‐based complexes, where Cu atoms are coordinated with well‐defined head and tail functional groups of organic linkers, may offer unexpected active sites that enhance ^*^CO adsorption. Recently, Li et al., developed stable coordination polymer catalysts by coordinating Cu with six phenyl‐1H‐1,2,3‐triazole derivatives, resulting in homogenized single‐site Cu active sites.^[^ ^318^ ^]^ The electronic properties of Cu were found to be highly tunable by adjusting the highest occupied molecular orbital (HOMO) energy of the ligands. Using CO diffuse FTIR, in situ Raman spectroscopy, and DFT calculations, they discovered a positive correlation between the binding strength of the ^*^CO intermediate and the HOMO energy of the ligands. This approach allowed them to fine‐tune the C─C coupling efficiency by adjusting ^*^CO coverage.

Cu‐based MOFs are another alternative promising catalyst due to their unique Cu sites linked by various ligands, creating a complex coordination environment that enables diverse ^*^CO adsorption configurations.^[^ ^80^, ^255^, ^322^, ^323^, ^324^ ^]^ MOFs can be structured in a wide variety of forms, with different linkages that lead to varied electronic states and multifunctional catalytic sites. Even within a single MOFs, Cu centers can be bridged by different ligands, resulting in multiple active sites during electrocatalysis. On the mass transfer front, the highly ordered porous networks of MOFs may improve gas transport and electrolyte permeability. Recent research has focused on identifying the roles of these distinct well‐defined Cu sites in enhancing ^*^CO coverage. For example, Chen et al., have reported Cu‐based MOFs featuring two types of Cu coordination sites, CuO_4_ and CuPc, which promoted C_2_H_4_ production in eCO_2_RR.^[^ ^322^ ^]^ Their studies demonstrated that ^*^CO produced and enriched on CuO_4_ sites can migrate and dimerize with ^*^CO intermediates adsorbed on neighboring CuPc sites, thereby promoting C─C coupling. Another typical example involves B‐imidazolate framework nanosheets with dual‐atom Cu_2_ sites bridged by Cl^−^ to effectively produce C_2_H_4_.^[^ ^255^ ^]^ These neighboring Cu monomers efficiently reduce reaction barriers in a tandem fashion by enriching charge on the Cu centers, leading to optimized interactions between CO_2_ and Cu sites. Despite these recent advances, challenges remain in the effective use of MOFs for CO_2_ conversion to C_2+_ products. A key difficulty lies in screening high‐performing Cu‐based MOFs, and the FE reported in the aforementioned studies is still inadequate for scalable applications.

Overall, while the impact of low‐coordinated Cu sites on ^*^CO coverage is well‐established, there is still a need for more precise atomic‐scale investigations to fully understand these dynamics. Future research must address the challenges posed by the evolution of catalysts under the strong cathodic conditions of eCO_2_RR, as well as the complexities in accurately quantifying unsaturated coordination sites. These factors complicate the identification and design of optimal catalytic sites. Incorporating heteroatoms like C and N and designing Cu‐based MOFs also show promise in creating unique coordination environments to improve ^*^CO coverage and boost C─C coupling, though further efforts are required to overcome existing limitations.

#### 4.1.2 Hetero Metal Incorporation

The incorporation of heterometal elements into Cu‐based catalysts to create neighboring dual sites has emerged as a promising strategy to regulate ^*^CO adsorption and promote C─C coupling during eCO_2_RR. Metals such as Zn,^[^ ^239^, ^325^, ^326^ ^]^ Ag,^[^ ^327^, ^328^, ^329^, ^330^, ^331^, ^332^, ^333^, ^334^, ^335^, ^336^, ^337^, ^338^ ^]^ and Au^[^ ^339^, ^340^, ^341^ ^]^ have shown particularly effective due to their ability to capture and activate CO_2_ molecules, facilitating ^*^CO coverage on adjacent Cu sites. However, the introduction of these heterometals also influences the behavior of Cu sites, with nearby Cu atoms exhibiting distinct properties and reactivity compared to those further away. This complexity has led to ongoing debates regarding the exact mechanism through which heterometals influence ^*^CO coverage, particularly around the tandem and functionalization roles. Recent advancements in in situ technologies and computational methods have facilitated further exploration of how various heterometals affect ^*^CO coverage and C_2+_ product generation. Researchers are not only improving product selectivity through rational catalyst design but are also focusing on the underlying mechanisms, providing fresh insights for future exploration.

**Figure 11:**
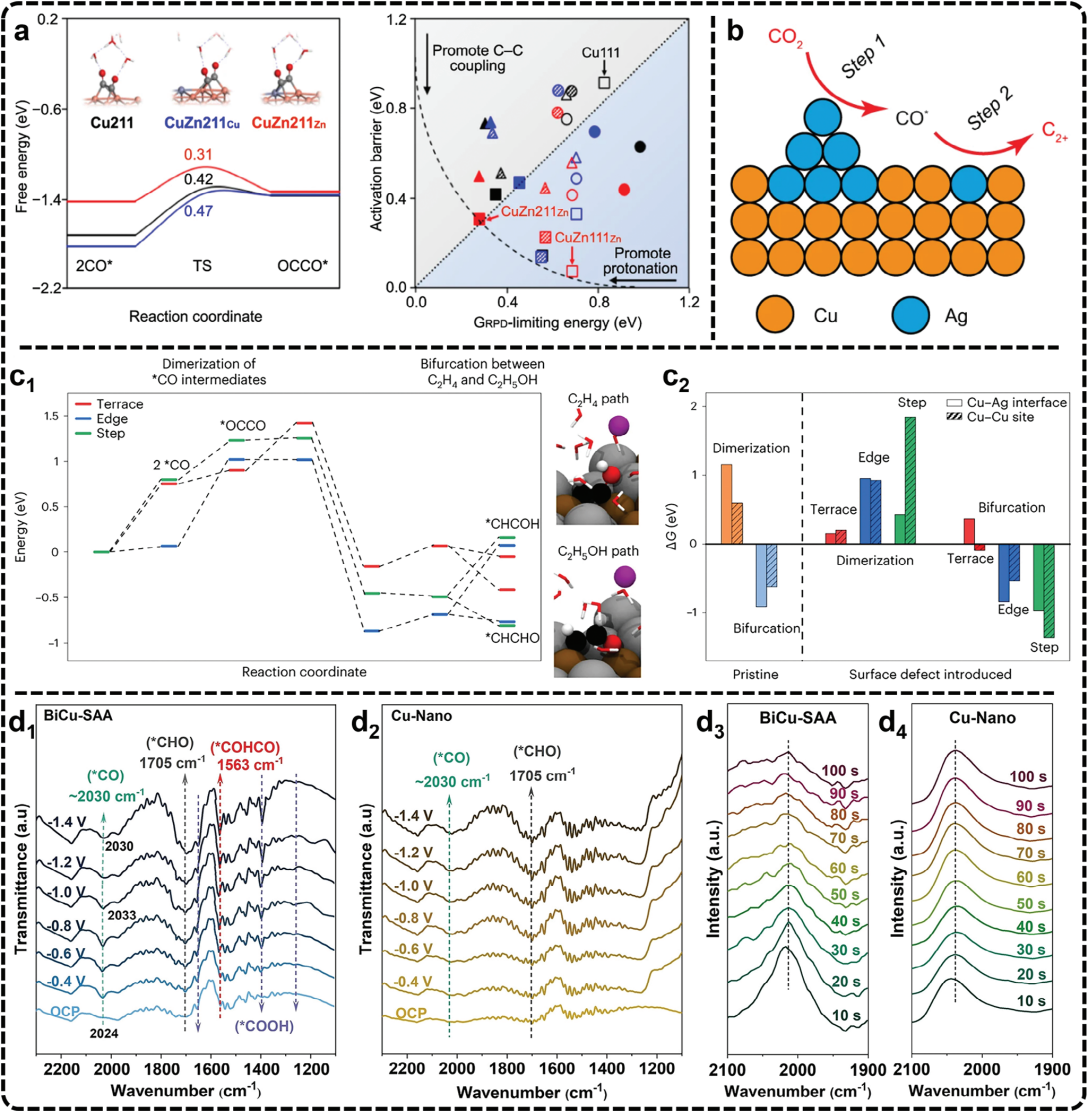
a) Activation energies for ^*^CO─^*^CO coupling on Cu and CuZn (211) and activation energies (black for Cu, blue for CuZn_Cu_, and red for CuZn_Zn_) plotted against the corresponding limiting potentials of the relevant paths (^*^CO─^*^CHO, ^*^CO─^*^COH, and ^*^CO─^*^CO). Adapted under terms of the CC‐BY license.^[^ ^342^ ^]^ Copyright 2023, Zhang et al. b) Schematic of the cascade catalysis mechanism over AgCu SANP. Adapted under terms of the CC‐BY license.^[^ ^347^ ^]^ Copyright 2023, Du et al. c_1_) Energy barrier diagram of CO_2_ electrolysis to C_2_H_4_ and C_2_H_5_OH on the terrace and surface defects of the Cu─Ag interface in supersaturated Ag─Cu catalyst and c_2_) reaction energy difference for dimerization and C_2_H_5_OH/C_2_H_4_ bifurcation. The energy barriers are compared between pristine and surface‐defect‐introduced Ag─Cu supersaturated surfaces at different active sites. Inset: models illustrating dimerization and bifurcation on the edge and terrace sites. Brown, silver, and purple spheres represent copper atoms, silver atoms, and K^+^ ions. Adapted with permission.^[^ ^344^ ^]^ Copyright 2023, Springer Nature. d) Detection of reaction intermediates of eCO_2_RR on both BiCu‐SAA and Cu‐Nano catalysts in different ways, including d_1_,d_2_) operando SR‐FTIR spectra at different potentials and d_3_,d_4_) time‐dependent in situ SEIRAS spectrum obtained at −1.10 V versus RHE. The results indicate increased ^*^CO on BiCu‐SAA. Adapted with permission.^[^ ^356^ ^]^ Copyright 2023, John Wiley and Sons.

Zn incorporation is a widely studied method to alloy with Cu or substitute lattice Cu in Cu‐based compounds due to their similar atomic properties. Recently, Zhong et al., have synthesized a series of Zn‐incorporated Cu catalysts to systemically compare ^*^CO coverage and investigate their influence on C_2+_ products selectively.^[^ ^342^ ^]^ Contrary to earlier views that Zn sites primarily generate CO for nearby Cu sites,^[^ ^239^, ^326^ ^]^ they found that Zn incorporation creates both Zn and Cu active sites. This dual‐site configuration enhances asymmetric ^*^CO binding and increases surface ^*^CO coverage, accelerating C─C coupling. As demonstrated in **Figure** **11**a, a comparison of activation barriers for ^*^CO─^*^CO coupling at various sites, along with the activation energies for different coupling pathways relative to their corresponding limiting potentials, reveals that CuZn_Zn_ sites are the most active. These sites exhibit lower limiting potentials, indicating their superior catalytic activity for C─C coupling during eCO_2_RR. Similarly, the introduction of Ag into Cu‐based catalysts has been recently shown to functionalize Cu sites effectively.^[^ ^86^, ^343^, ^344^ ^]^ Joo et al., have reported a metal alloy where Cu clusters are spatially dispersed in a crystalline silver lattice to promote eCO_2_RR toward C_2_H_5_OH with high selectivity.^[^ ^344^ ^]^ The synergistic interaction between Cu─Cu sites and Cu─Ag interfaces enhances and diversifies ^*^CO binding at terrace and defect sites, promoting ^*^CO─^*^CO coupling. As illustrated in Figure 11c, the dimerization barrier for ^*^CO─^*^CO coupling decreases at these sites, while the stabilized C_2_H_5_OH intermediates at the step sites guide the reaction pathway toward C_2_H_5_OH production. This observation aligns with Qiao et al., perspective, which suggests that Ag incorporation not only functionalizes Cu sites but also steers ^*^CO adsorption configurations toward more favorable catalytic pathways.^[^ ^333^ ^]^ These findings challenge traditional views of tandem catalysis mechanisms and highlight the complex role of these heterometals in Cu‐based catalysts.

Even with these new findings, the tandem mechanism remains central to other recent studies.^[^ ^104^, ^140^, ^343^, ^345^, ^346^ ^]^ Zhang et al., have investigated the role of Ag sites in a Cu‐based catalyst, suggesting that weaker ^*^CO binding strength on Ag sites can lead to preferential adsorption of CO on certain Cu sites, thereby increasing local ^*^CO coverage and improving the C_2_H_4_ selectivity by several times through ^*^CO─^*^CHO coupling.^[^ ^104^ ^]^ Another study introduced Ag@Cu_2_O cascade nanoreactors with adjustable shell thicknesses.^[^ ^346^ ^]^ In this design, CO generated at the Ag core spills over and gets trapped in the Cu_2_O shell. An optimal Cu_2_O shell thickness allows for better diffusion of CO_2_ and effective confinement of CO, which increases CO concentration and enhances the C─C coupling. These works are consistent with reaction mechanisms reported for CuAg catalysts, such as those composing Ag single atoms and Ag nanoparticles (AgCu SANP), where Ag nanoparticles primarily produce CO, while Cu sites near Ag single atoms facilitate subsequent ^*^CO─^*^CHO coupling (Figure 11b).^[^ ^347^ ^]^ In essence, these perspectives resonate with traditional views of tandem catalysts involving Ag or Au incorporated into Cu‐based systems.^[^ ^327^, ^331^, ^334^, ^335^, ^336^, ^339^, ^340^, ^341^, ^348^, ^349^, ^350^, ^351^, ^352^, ^353^ ^]^ The superior CO formation ability of Ag and Au provides a substantial amount of CO for Cu sites, helping to overcome the slow CO_2_ activation step (CO_2_‐to‐CO) on Cu sites. Consequently, the CO_2_ reduction to C_2+_ products likely follows a sequential conversion pathway. A notable example is Jaramillo et al., pioneering work with Au nanoparticles on polycrystalline Cu foil, where CO_2_ reduction on Au generates high local CO concentrations that are further reduced to alcohols like C_2_H_5_OH and n‐propanol on the Cu sites.^[^ ^339^ ^]^ Additionally, several studies have noted that the CO formation reaction (CO_2_ + H_2_O + 2e^−^ → CO + 2OH^−^) can lead to a localized increase in pH. As a result, heterometal‐incorporated tandem catalysts designed to enhance ^*^CO coverage may also raise the local pH around Cu sites. This pH modulation is significant from the materials perspective, as it helps stabilize essential charged Cu species, which are believed to be critical for promoting C─C coupling, as discussed in the following section.^[^ ^71^, ^339^, ^348^, ^354^, ^355^ ^]^

In addition to Zn, Ag, and Au incorporation, the functionalization and synergistic effects of other metals in Cu‐based catalysts offer intriguing avenues for optimizing ^*^CO coverage and enhancing catalytic performance. Unlike tandem catalyst designs, these approaches focus on altering the electronic structure of Cu sites or introducing multiple active sites to influence ^*^CO adsorption and coverage. For instance, the incorporation of Bi into single‐atom decorated Cu alloy catalysts (BiCu‐SAA) has been shown to positively charge metallic Cu, lowering the energy required for CO_2_ adsorption and activation while increasing ^*^CO coverage and production of C_2+_ products.^[^ ^356^ ^]^ Evidence from operando FTIR with Synchrotron radiation (SR‐FTIR) demonstrates higher intensities of ^*^CO species at 2030 cm^−1^ and ^*^CHO species at 1705 cm^−1^ on BiCu‐SAA compared to Cu catalysts, indicating increased ^*^CO coverage (Figure 11d_1_,d_2_). The observation of O─H deformation, C─O stretching, and C═O stretching further supports CO_2_ activation to ^*^CO. Additionally, in situ SEIRAS shows a rapid decline of ^*^CO within seconds on BiCu‐SAA (Figure 11d_3_), suggesting that C─C coupling is accelerated by the consumption of ^*^CO species. Likewise, active machine learning models have identified Al‐Cu alloys and Mg‐modified Cu catalysts as materials providing multiple sites with near‐optimal CO binding, enhancing C─C coupling.^[^ ^357^, ^358^ ^]^ Experimentally, the effectiveness of Mg incorporation has been demonstrated by increased ^*^CO coverage observed in Cu_2_Mg intermetallic compounds.^[^ ^359^ ^]^

**Figure 12:**
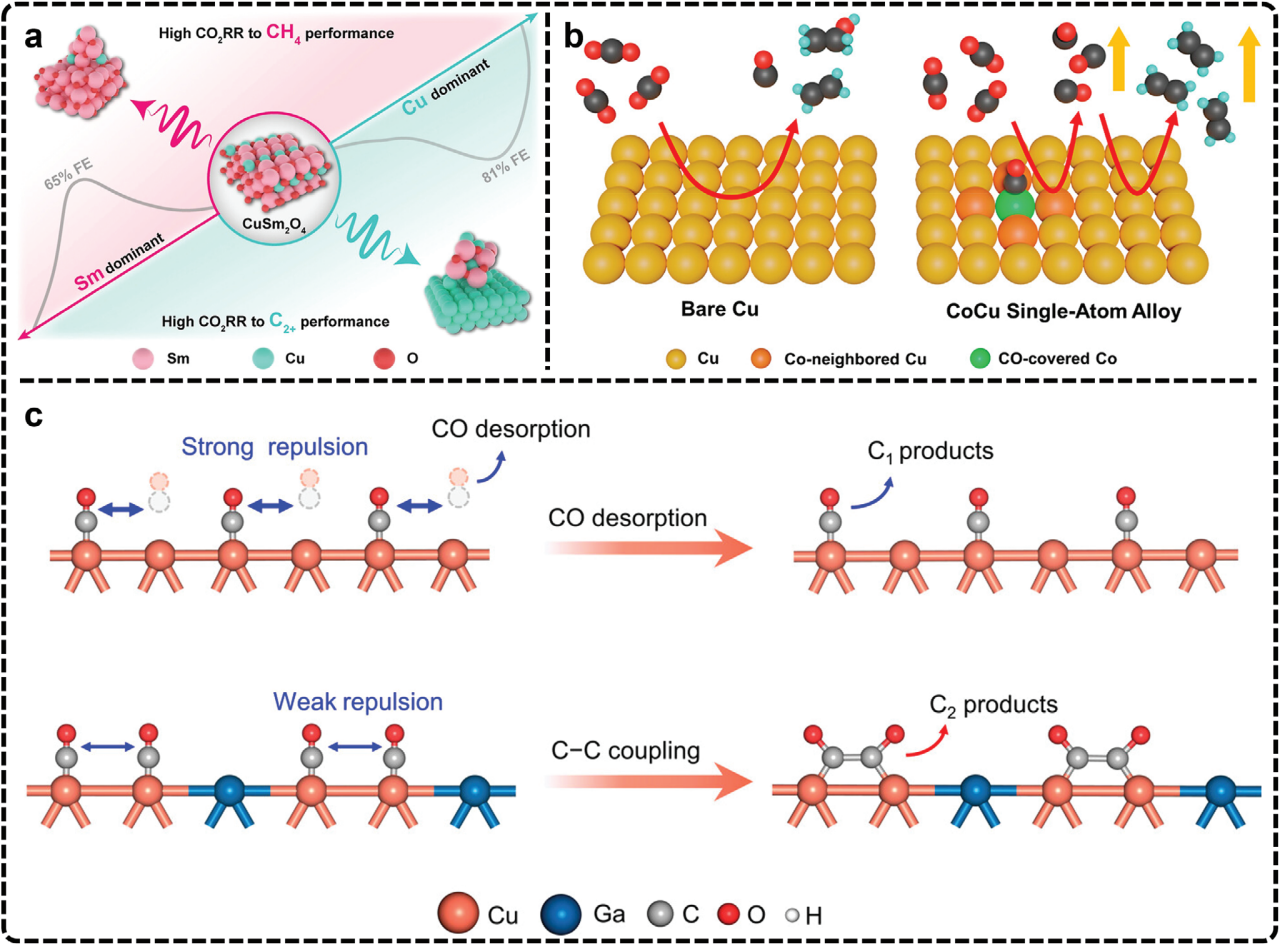
Schematic Illustration of regulating the eCO_2_RR pathway for C_2+_ and CH_4_ production by CuSm‐O_x_. Reproduced with permission.^[^ ^360^ ^]^ Copyright 2023, American Chemical Society. b) Schematic of the tandem electroreduction CO_2_ for CoCu SAA. Reproduced with permission.^[^ ^362^ ^]^ Copyright 2023, American Chemical Society. c) Schematic showing the ^*^CO repulsion on Cu and Cu_9_Ga_4_ intermetallic compound. On conventional Cu (100) sites, the high density of Cu atoms results in strong ^*^CO repulsion, thus reducing the surface θ^*^_CO_ and subsequent C─C coupling. On the Cu_9_Ga_4_ intermetallic compound, Ga atoms bind ^*^CO weakly with an extended Cu─Cu distance to reduce the ^*^CO repulsion. Therefore, a high surface θ^*^_CO_ can be achieved for efficient C─C coupling and C_2_ formation. Reproduced with permission.^[^ ^363^ ^]^ Copyright 2023, American Chemical Society.

On the other hand, incorporating unconventional metals into Cu‐based catalysts often addresses alternative challenges, such as regulating intermediate adsorption and optimizing mass transfer. Rare‐earth metals, for instance, have been discovered to diversify ^*^CO adsorption on Cu‐based catalysts through electronic structure modification, switching eCO_2_RR products from CH_4_ to C_2+_ by adjusting the rare‐earth metal content.^[^ ^360^, ^361^ ^]^ Specifically, a small amount of Sm incorporation enhances ^*^CO binding on stable Cu^2+^ sites in CuSm_2_O_4_ and Cu‐phase coexisted catalysts, leading to a C_2+_ product FE of up to 81% (**Figure** **12**a). However, when Sm‐based phases dominate, CH_4_ becomes the main product. Beyond metal content, the applied potential also plays a role in product selectivity by influencing ^*^CO coverage. Sb‐modified Cu catalysts have been shown to produce either CO or C_2+_ products depending on the potential.^[^ ^97^ ^]^ At lower potentials, the retention of surface oxygen facilitates CO production by promoting ^*^COOH formation and reducing ^*^CO adsorption. At higher potentials, Sb enhances ^*^CO adsorption, favoring C─C coupling and increasing C_2+_ selectivity. These examples illustrate how incorporating hetero metals can fine‐tune intermediate behavior, allowing for greater control over product outcomes in eCO_2_RR.

Furthermore, the role of local ^*^CO enrichment and its potential repulsion effect in promoting C─C coupling remains a debated topic. Oh et al., challenged Co incorporation into Cu catalysts (CuCo‐SAA) to improve C_2+_ product selectivity, even though Co typically leads to CO poisoning and catalyst deactivation.^[^ ^362^ ^]^ Their study reveals that trace Co amounts accelerate CO formation rates by leveraging CO‐poisoned Co atomic sites, which in turn enhance ^*^CO intermediates coverage on adjacent Cu sites, thereby offering more opportunities for C─C coupling to produce key intermediates ^*^CHCOH (Figure 12b). However, Zhang et al., cautioned that increased ^*^CO coverage on Cu sites could lead to repulsion between adsorbates, which impaired mass transfer efficiency.^[^ ^363^ ^]^ To mitigate this, they proposed atomically ordered Cu_9_Ga_4_ intermetallic compounds, which featured interspaced active and inert ^*^CO binding sites (Figure 12c). This structure aims to optimize ^*^CO coverage by elongating Cu─Cu distances to enhance the catalytic efficiency. These opposing viewpoints highlight the complexity of designing catalysts that effectively regulate ^*^CO coverage without compromising other aspects of catalytic performance.

Overall, heterometal incorporation has emerged as a powerful and feasible strategy for enhancing C─C coupling efficiency in eCO_2_RR. Metals like Zn, Ag, and Au have demonstrated their ability to modulate ^*^CO adsorption and coverage, improving selectivity toward C_2+_ products. These metals not only affect Cu site reactivity but also introduce synergies that can either promote or inhibit specific catalytic pathways. Recent advances in in situ technologies and computational methods have provided deeper and extra insights into these mechanisms, offering new directions for improving selectivity. Looking ahead, continued exploration of uncommon metal incorporation will be essential for regulating intermediates adsorption and optimizing mass transfer. Developing catalysts that balance ^*^CO coverage with efficient mass transfer remains a challenge. Nevertheless, this line of research holds the potential to unlock new eCO_2_RR pathways, driving further advancements in the production of C_2+_ products.

#### 4.1.3 Heterogeneous Component Modification

In addition to incorporating heterometals, modifying Cu‐based catalysts with heterogeneous components such as metal complexes and organic molecules has proven effective in regulating ^*^CO coverage to enhance C─C coupling. These heterogeneous components serve various functional roles in facilitating the reaction process. Metal oxides, known for their stability, are frequently used to decorate Cu‐based catalysts. However, only a few studies have explored the impact of metal oxide incorporation on ^*^CO adsorption and coverage. This is partly due to metal oxides lacking sufficient active sites to activate CO_2_ under reductive conditions. Typically, improvements in C─C coupling efficiency are attributed to changes in the oxidation state of Cu, influenced by interactions with metal oxides. For instance, ZrO_2_ nanoparticles on Cu foil and CuO nanoparticles on Al_2_CuO_4_ nanosheets demonstrate how metal oxide nanoparticles can interact with Cu‐based materials to form specific interfaces.^[^ ^275^, ^364^ ^]^ These interactions facilitate electron transfer between the metal oxides and Cu sites, altering the electronic structure of Cu and creating favorable conditions for ^*^CO adsorption, thus promoting the subsequent ^*^CO─^*^CO coupling process. This often involves maintaining positively charged Cu sites, as will be discussed in the following section. Additionally, nitrogen‐doped carbon (N‐C) and carbon nanotubes as support structures have also been shown to benefit ^*^CO coverage.^[^ ^365^, ^366^, ^367^ ^]^ These studies, supported by DFT calculations, demonstrate that the C‐support interface favors promoting ^*^CO adsorption, reinforcing the understanding of mechanisms behind improved selectivity for C_2+_ products.

**Figure 13:**
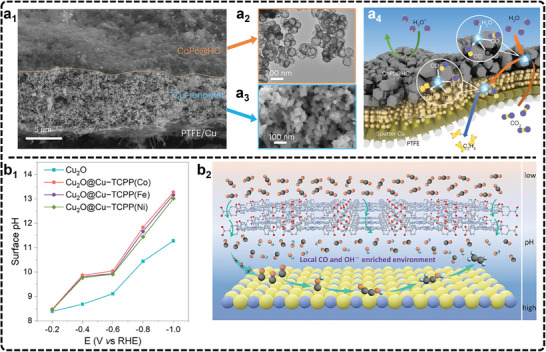
a) Electron microscope images and schematic diagram. Adapted with permission.^[^ ^355^ ^]^ Copyright 2023, Springer Nature. a_1_) Cross‐sectional SEM image of CoPc@HC/Cu tandem electrode (PTFE, polytetrafluoroethylene). a_2_) TEM image of upper CoPc@HC catalyst layer; a_3_) SEM image of lower Cu catalyst layer with the Cu–ionomer interface. a_4_) Schematic of the spatially decoupled strategy via tandem catalysis, showing the electron (e^−^) transfer and mass transport in acidic eCO_2_RR. b_1_) Plots of the calibrated surface pH against the applied potential based on the Raman results and b_2_) schematic illustration of the Cu−TCPP(M) (M═Co, Fe) overlayer on Cu_2_O to boost local CO and OH^−^ concentrations at the interface. Adapted with permission.^[^ ^71^ ^]^ Copyright 2024, John Wiley and Sons.

Similar to the heterometal, recent research has highlighted the promise of metal complexes with high CO production capabilities in modifying Cu‐based catalysts. These materials can provide effective CO generation sites for Cu, thereby facilitating tandem mechanisms in the catalytic process.^[^ ^27^, ^33^ ^]^ For instance, the Ni─N─C structure with high porosity has been utilized to produce CO for Cu sites in Cu‐based catalysts.^[^ ^288^, ^368^ ^]^ Researchers have observed significantly increased CO signals and attribute this to the enhanced mass transfer provided by the high porosity of these materials, which benefits promoting the subsequent C─C coupling. Similarly, several reports demonstrate that incorporating Ni single atoms into Cu catalysts can enhance C_2+_ product selectivity. This is achieved by leveraging the superior CO formation ability of Ni single atoms to improve ^*^CO enrichment on Cu sites, aligning with the typical tandem catalysis mechanism.^[^ ^219^, ^369^, ^370^, ^371^, ^372^, ^373^, ^374^, ^375^ ^]^ Notably, the wide CO generation potential of Ni sites highlights that integrating Ni single atoms can help bridge potential mismatches between CO production and ^*^CO─^*^CO coupling in Cu‐based tandem catalysts.^[^ ^370^, ^371^ ^]^ This is crucial, as aligning CO generation with C─C coupling efficiency is essential for optimizing product yields in tandem catalysis. Another notable example is cobalt phthalocyanine (CoPc), an excellent CO producer in eCO_2_RR.^[^ ^376^, ^377^, ^378^ ^]^ When coupled with Cu‐based catalysts, CoPc shows local enrichment of ^*^CO on Cu sites, favoring C─C coupling and C_2+_ product formation in acidic media.^[^ ^355^, ^379^ ^]^ Sargent et al., have shown a three‐layer electrode configuration (**Figure** **13**a_1_) in which CO_2_ gas first passes through a gas diffusion layer and an ionomer‐coated Cu nanoparticle layer (Figure 13a_3_) and finally reaches an atomically dispersed CoPc catalyst layer on hollow carbon support (CoPc@HC) (Figure 13a_2_). In this structure, CO_2_ is converted to CO, which then diffuses back to the Cu nanoparticle layer for C─C coupling to produce C_2_H_4_ and other C_2+_ products (Figure 13a_4_). Porphyrin‐based metal complexes have also been shown to catalyze the conversion of CO_2_ to CO, increasing CO concentration near the Cu surface.^[^ ^264^ ^]^ To effectively harness the CO generated from metalloporphyrin, a recent study encapsulated Cu_2_O nanocubes into metalloporphyrin frameworks, achieving improved C_2+_ product yields.^[^ ^71^ ^]^ This approach not only ensures efficient CO mass transfer but also enhances the local pH around Cu sites, as indicated by Raman spectroscopy (Figure 13b_1_). This pH modulation is thought to lower the energy barrier for C─C coupling, further boosting catalytic performance (Figure 13b_2_). A similar design is accomplished by incorporating atomically isolated Cu(II) porphyrin and Cu(II) bipyridine sites on a carbon nanotube scaffold with dispersed Cu_2_O nanoparticles, where the Cu(II) porphyrin organic framework increases local ^*^CO coverage, Cu_2_O enables C─C coupling, and the scaffold enhances the electron conductivity and mass transfer stability.^[^ ^367^ ^]^

The modification of Cu catalysts with organic molecules is another significant approach to improving ^*^CO coverage. This strategy typically involves interaction between the molecules and intermediates without altering the morphology or electronic properties of the Cu substrate. For instance, amines,^[^ ^65^, ^380^ ^]^ and polymers such as polyacrylamide,^[^ ^381^ ^]^ polyaniline,^[^ ^382^ ^]^ polypyrrole^[^ ^383^ ^]^ modified Cu catalysts have been shown to enable to enhance ^*^CO adsorption on Cu sites near the molecule, thereby increasing local ^*^CO coverage and boosting C─C coupling efficiency. This effect is achieved through mechanisms such as charge donation, chemical stabilization via hydrogen bonds, or electrophilic attraction of organic groups around the ^*^CO intermediates. Furthermore, Yan et al., recently introduced a surface‐confinement strategy on Cu_2_O nanoparticles by long alkyl chain grafting to create a spatially confined environment, impeding ^*^CO intermediates detachment and promoting C─C coupling in the eCO_2_RR, highlighting the interaction between molecules and intermediates.^[^ ^384^ ^]^

Some organic molecules, like N‐aryl‐substituted tetrahydro‐bipyridine, 4‐dimethylaminopyridine, and arylpyridinium dimers, can also influence ^*^CO coverage by altering the adsorption mode.^[^ ^291^, ^385^, ^386^, ^387^ ^]^ Sargent et al., have indicated that organic molecules can stabilize ^*^CO on the Cu surface by creating different ^*^CO adsorption configurations through weak interactions.^[^ ^291^ ^]^ They found that the ratio of atop to bridge ^*^CO configurations is highly correlated with reaction selectivity, highlighting the potential of organic molecules in regulating product distribution. Additionally, electrophilicity is another critical factor that impacts product selectivity in eCO_2_RR, as the intermediate coverage and C─C coupling are closely correlated with the surface charge on the catalyst.^[^ ^65^ ^]^ Recent work reveals that modifying Cu with highly electrophilic molecules, such as trans‐1,2‐bis(4‐pyridyl)ethylene, can stabilize ^*^CO and promote ^*^CO─*COH coupling.^[^ ^388^ ^]^ Conversely, using molecules with lower electrophilicity, like 1,2‐bis(4‐pyridyl)ethane, leads to the release of hydrogenated ^*^CO, resulting in CH_4_ production. This example provides a pioneering approach for designing molecule‐modified Cu‐based catalysts to better regulate product distribution.

Overall, the development of Cu‐based catalysts modified with various heterogeneous components has deepened our understanding and expanded the strategies for enhancing C─C coupling during eCO_2_RR. These innovative catalysts offer valuable insights into how synergistic interactions between catalytic sites and intermediates can influence the electrochemical reduction pathway and energy barriers. Despite these advancements, eCO_2_RR for producing C_2+_ products still faces challenges related to efficiency and stability. For example, some organic molecules risk reduction and dissolution under reaction conditions. By integrating the functional roles of the strategies discussed, future catalyst designs can become more targeted and effective. These advancements also hold the potential to reduce reliance on trial‐and‐error experimentation, leading to the development of more efficient and selective Cu‐based catalysts for eCO_2_RR.

### 4.2 Optimizing Intermediate Protonation

The formation of C_2+_ products typically involves ^*^CO intermediates that undergo protonation either before or after C─C coupling. According to the Brønsted–Evans–Polanyi relations, weaker ^*^CO adsorption combined with higher coverage is crucial for effective C─C coupling.^[^ ^389^, ^390^, ^391^ ^]^ However, the adsorption scaling relation suggests that both protonation and C─C coupling rely on the stabilizing key intermediates such as ^*^CO, ^*^CHO, ^*^COH, and ^*^CH_x_, and post‐coupling species like ^*^OCCOH, and ^*^OCCHO.^[^ ^75^, ^125^, ^392^, ^393^, ^394^ ^]^ Achieving the optimal balance between intermediate adsorption/release and protonation is therefore critical. Since protonation is a PCET process, the presence of a certain amount of adsorbed hydrogen (^*^H) around the active sites is essential for reacting with carbon‐containing intermediates (e.g., ^*^CO, ^*^CO─^*^CO, ^*^OCCOH).^[^ ^60^, ^395^, ^396^, ^397^, ^398^ ^]^ However, excessive accumulation of ^*^H may endow undesirable side reactions, such as the HER or consecutive protonation of ^*^C_1_ intermediates, resulting in by‐products like CH_4_ or HCOOH.^[^ ^231^, ^262^, ^263^, ^301^, ^399^, ^400^, ^401^, ^402^, ^403^, ^404^, ^405^, ^406^ ^]^ For instance, Ir sites with exceptional water dissociation capability can promote continuous proton feeding to ^*^CO intermediates on Cu sites, leading to the formation of CH_4_ in Ir single atoms doped Cu catalysts.^[^ ^402^ ^]^ Additionally, Cu particles with different atomic configurations can influence ^*^CHO transformation, with single atoms favoring hydrogenation with ^*^H to produce CH_4_ and clusters promoting ^*^CHO coupling with ^*^CO to generate C_2_H_4_.^[^ ^60^ ^]^ Therefore, catalyst design requires careful regulation of ^*^H generation, distribution, and consumption to stabilize protonated intermediates, thus steering the reaction pathways toward C_2+_ products. This subsection reviews recent useful strategies for controlling ^*^H on Cu‐based catalysts, including the heteroatom functionalization, oxides and hydroxides introduction, and organic complex linking, aiming to illuminate their impact on the interaction between active sites, ^*^H‐containing intermediates, and C_2+_ product selectivity.

#### 4.2.1 Heteroatom Functionalization

**Figure 14:**
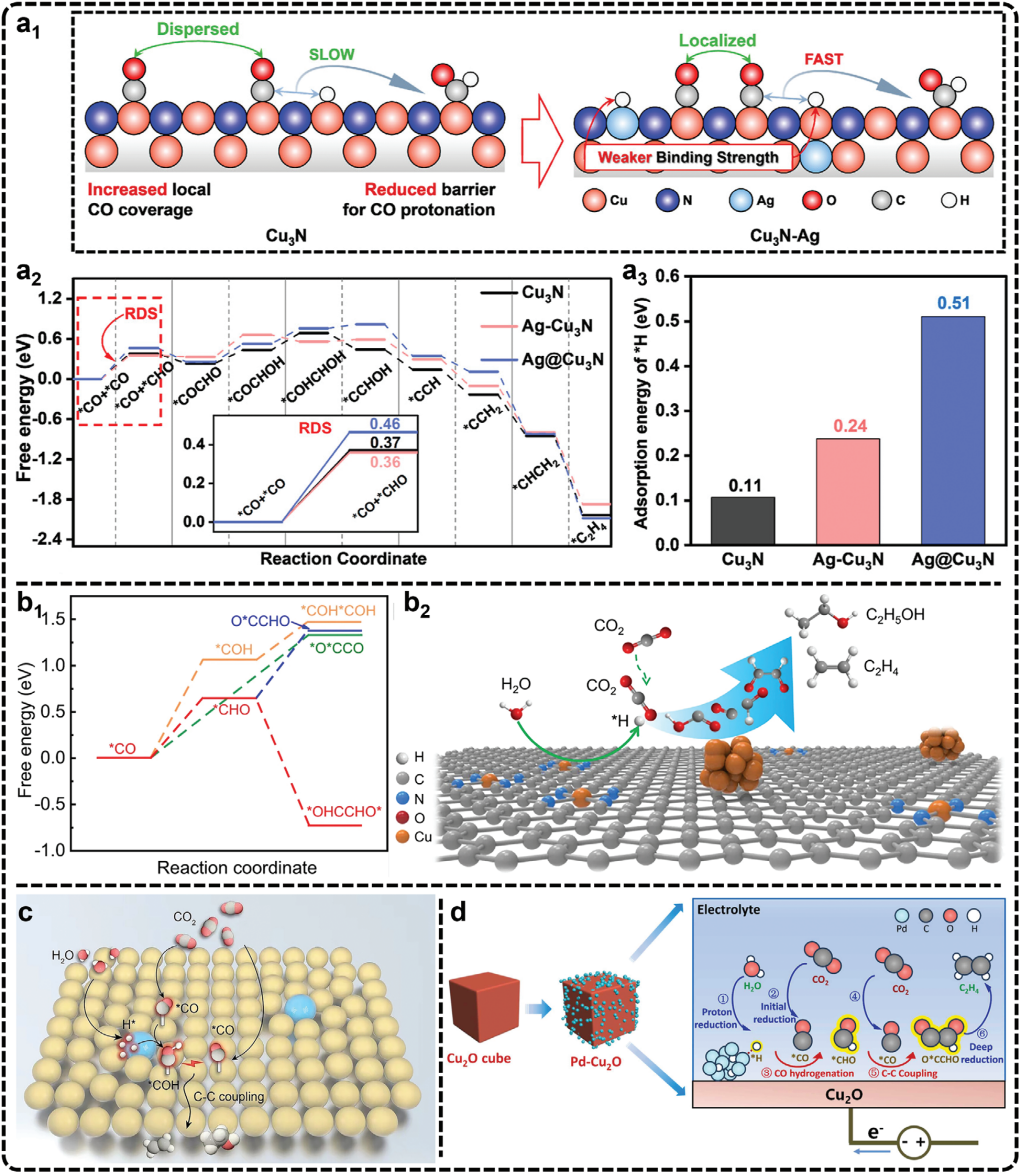
a) Theoretical calculation of Ag incorporation into the Cu_3_N catalyst, including the a_1_) schematic of the introduction of heterometal Ag into Cu_3_N with weaker binding strength and the reaction intermediates of eCO_2_RR can increase local CO coverage and facilitate ^*^CO protonation to ^*^CHO, a_2_) the free‐energy diagram of the optimal CO‐to‐C_2_H_4_ reaction pathway on different substrates (inset showing a detailed free‐energy diagram of the RDS), and a_3_) adsorption energy of ^*^H on different substrates. Adapted with permission.^[^ ^104^ ^]^ Copyright 2024, American Chemical Society. b_1_) Free energy diagram for the eCO_2_RR to describe the possible C─C coupling step from ^*^CO over Cu (111) and b_2_) proposed reaction mechanism for eCO_2_RR to C_2_ products on the M‐Cu_1_/Cu_NP_, showing the ^*^CHO generated from Cu‐N_4_ sites dimerization to form ^*^OHCCHO^*^ is exergonic.^[^ ^76^ ^]^ Adapted under terms of the CC‐BY license.^[^ ^76^ ^]^ Copyright 2023, Feng et al. c) Schematic of three distinct catalytic sites to promote electroreduction CO_2_ to C_2+_ products on a Pd atomically dispersed in Cu catalyst. Reproduced with permission.^[^ ^408^ ^]^ Copyright 2024, John Wiley and Sons. d) Schematic of the structure and eCO_2_RR mechanism of Pd‐Cu_2_O. The Cu sites, in conjunction with the Pd‐H sites, activate ^*^CO at the Pd‐Cu interface, forming stable ^*^CHO intermediates. They facilitate the coupling of ^*^CHO and ^*^CO intermediates, ultimately resulting in a nearly five fold increase in the C_2_H_4_/H_2_ production rate. Reproduced with permission.^[^ ^62^ ^]^ Copyright 2024, Elsevier.

Due to the competitive HER during eCO_2_RR, many previous studies have overlooked the role of appropriate ^*^H adsorption in facilitating protonation on Cu sites to form ^*^CH_x_O_y_ intermediates for further coupling.^[^ ^335^ ^]^ Typically, Cu sites exhibit moderate ^*^CO and weak ^*^H adsorption, enabling them to catalyze eCO_2_RR to C_2+_ products but with inherently limited activity and selectivity. Introducing heteroatoms to functionalize Cu sites or construct functional sites and interfaces is a promising approach to optimize ^*^H activation, often involving both metals and a few nonmetals. For instance, while the impact of Ag on ^*^CO coverage for Cu sites has been well‐studied, the protonation process has been underexplored. However, recent studies have shown that Ag can promote the formation of hydrogenated ^*^CHO intermediates through abundant Ag/Cu interfaces in Ag/Cu bimetallic catalysts.^[^ ^63^, ^116^ ^]^ This challenges the conventional belief that Ag/Cu interfaces are inert to ^*^H adsorption, which traditionally serves to suppress H_2_ generation, and highlights the importance of protonation alongside tandem effects to enhance C_2_H_4_ production. Further, recent research into Ag‐doped Cu_3_N catalysts has revealed that Ag atoms optimize ^*^H binding strength at nearby Cu sites, favoring ^*^CO protonation to form ^*^CHO and lowering the energy barrier for ^*^CO─^*^CHO coupling compared to ^*^CO─^*^CO coupling.^[^ ^104^ ^]^ This effect is driven by ^*^H migration, where weakly adsorbed ^*^H on Ag sites transfers to adjacent ^*^CO on Cu sites, as visualized in **Figure** **14**a_1_. Furthermore, DFT calculations further support these findings by illustrating reduced energy consumption for C─C coupling and ^*^H adsorption pathways (Figure 14a_2_,a_3_). These findings highlight the shortcomings of earlier studies on Cu‐Ag catalysts, which mainly concentrated on inhibiting ^*^H adsorption and H_2_ evolution, without acknowledging the critical role that controlled ^*^H adsorption plays in facilitating C_2+_ product formation through effective protonation and C─C coupling.^[^ ^6^, ^27^, ^265^, ^407^ ^]^ However, beyond Ag, other CO‐producing metals like Zn and Au have yet to be thoroughly explored for their influence on the protonation of intermediates, representing an area of interest for future investigation.

While weak ^*^H adsorption on Ag sites is beneficial in certain cases, a contrasting perspective argues for stronger ^*^H adsorption to increase ^*^H coverage around intermediates, enhancing the likelihood of protonation. Introducing heteroatoms plays a critical role by creating functional sites that generate active ^*^H, optimizing intermediate protonation. This is particularly important in alkaline and neutral electrolytes, where protons are derived from water dissociation, as single Cu sites alone are often insufficient for efficient water cleavage. Recent studies have demonstrated that introducing metals with intrinsic water dissociation ability can enhance protonation on Cu catalysts. For example, single‐atom Ga‐anchored F‐doped Cu_2_O catalysts exhibit dual active sites, where Ga enhances water dissociation, increasing ^*^H concentration around Cu sites.^[^ ^66^ ^]^ This promotes ^*^CO protonation to ^*^CHO, favoring a lower energy pathway for ^*^CO─^*^CHO coupling and improving catalytic efficiency, resulting in increased FE for C_2+_ products. Further investigations by Han's group explored functional sites for water dissociation. They found that incorporating N into Cu‐based catalysts significantly enhances H_2_O dissociation, generating active ^*^H. In one example, a Cu complex catalyst composed of four N‐coordinated Cu single atoms and Cu nanoparticles (M‐Cu_1_/Cu_NP_) achieved a C_2+_ products FE of 75.4%.^[^ ^76^ ^]^ This effect is driven by ^*^H migration from N‐coordinated Cu sites to Cu nanoparticles, promoting ^*^CHO intermediate enrichment and dimerization, as supported by free energy calculations (Figure 14b). Following this design approach, the group recently developed a three‐functional‐active‐sites Cu catalyst with Pd atomically dispersed in it.^[^ ^408^ ^]^ In this configuration, Pd atoms promote H_2_O dissociation, while Cu atoms, far from and close to Pd atoms, activate CO_2_ and convert the generated ^*^CHO intermediates, respectively (Figure 14c). In fact, Pd is a well‐known heteroatom used to modify Cu‐based catalysts for regulating local ^*^H coverage due to its excellent proton capture abilities, as demonstrated in Pd nanoparticles coated Cu_2_O, Pd‐doped Cu, and dispersive CuO_x_ and PdO dual nanoclusters catalysts.^[^ ^62^, ^409^, ^410^ ^]^ As illustrated by the reaction pathways of Pd‐doped Cu catalysts in the schematic (Figure 14d), Pd nanoparticles offer abundant ^*^H adsorption sites, promoting ^*^CO protonation and accelerating C─C coupling on Cu sites. Theoretically, given that metallic Pd is located in the strong adsorption region of ^*^H and exhibits a strong adsorption affinity for H or even the ability to form PdHx, intermediate protonation is more likely to occur at nearby Cu sites. However, for effective C_2+_ product formation, protonation must not impede C─C coupling to avoid the production of undesired byproducts such as CH_4_ or HCOOH.^[^ ^411^, ^412^, ^413^ ^]^

In summary, despite significant achievements, the debate over whether weak or strong ^*^H adsorption is more effective in promoting ^*^CO protonation on Cu sites remains unresolved. Current theories are primarily based on computational models, with a lack of direct experimental evidence, as the evolution of ^*^H is difficult to detect accurately by conventional techniques. Future research should prioritize experimental investigations into the role of heteroatoms in regulating ^*^H adsorption on Cu‐based catalysts, as achieving an optimal ^*^H coverage balance is crucial for guiding reaction pathways through improving protonation, stabilizing key intermediates, and promoting C─C coupling, ultimately leading to more efficient C_2+_ product formation. This in turn requires advanced experimental methodologies and equipment capable of precisely tracking ^*^H migration and protonation processes. Additionally, when introducing heteroatoms, it is essential to consider their effects on both nearby and distant Cu sites, as improvements should not come at the expense of the catalytic properties of other active sites. Comprehensive studies on various heteroatoms and their interactions with Cu surfaces will offer valuable insights and guide the development of next‐generation catalysts for specific product targets in eCO_2_RR.

#### 4.2.2 Oxide and Hydroxide Introduction

**Figure 15:**
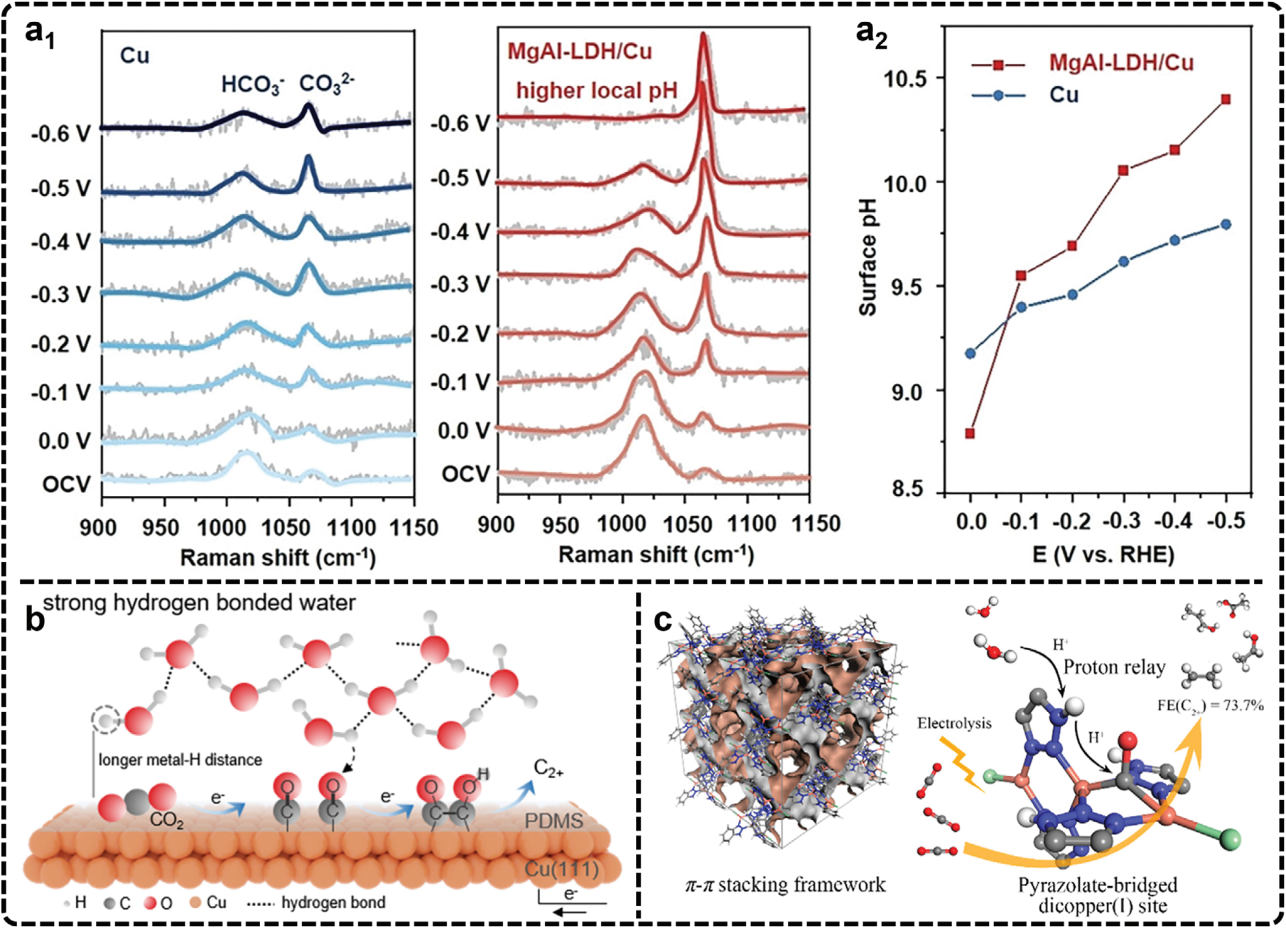
a_1_) In situ Raman spectra obtained from Cu and MgAl‐LDH/Cu electrodes biased from OCV to −0.6 V (vs RHE), respectively, and a_2_) the pH values are calculated from the in situ Raman spectra at various applied potentials ranging from 0 to −0.5 V (vs RHE). All the spectra are collected from an H‐cell with CO_2_‐saturated 1.0 m KHCO_3_ electrolyte flowing after electrolysis begins for 120 s. The relative ratio of [HCO_3_^−^] to [CO_3_^2−^] can be calculated from the integrated Raman peak area ratios of dissolved HCO_3_^−^ (1014 cm^−1^) and CO_3_^2−^ (1067 cm^−1^), which is correlated with the surface pH values in the vicinity of the electrode in accordance with the Henderson‐Hasselbach equation for a CO_3_^2−^/HCO_3_^−^ system. Adapted with permission.^[^ ^417^ ^]^ Copyright 2023, John Wiley and Sons. b) Schematics of the hydrogen bonding structure of interfacial water and proposed reaction pathway on Cu(111)@PDMS. Reproduced with permission.^[^ ^431^ ^]^ Copyright 2024, American Chemical Society. c) Pore structure stacked by CuBtz molecules via *π–π* interactions and the schematic showing the uncoordinated nitrogen atoms of benzotriazolates in the immediate vicinity can act as proton relays and cooperate with the dicopper(I) site to promote the hydrogenation process of the ^*^CO intermediate and the C─C coupling, resulting in the highly selective electroreduction of CO_2_ to C_2+_ products. Reproduced with permission.^[^ ^436^ ^]^ Copyright 2022, American Chemical Society.

Metal oxides and hydroxides possess the activity for H_2_O cleavage but weakly adsorb active ^*^H,^[^ ^209^, ^414^, ^415^ ^]^ making them ideal candidates for enhancing proton supply in Cu‐based catalysts. Incorporating these materials into Cu‐based systems introduces functional active sites that facilitate water dissociation and protonation. In a Ce(OH)_x_‐doped Cu catalyst, it is hypothesized that Ce(OH)_x_ sites accelerate water dissociation and influence ^*^H coverage on adjacent Cu sites, promoting protonation of ^*^CHCOH intermediates, which helps steer product selectivity toward C_2_H_5_OH formation rather than C_2_H_4_ in eCO_2_RR.^[^ ^416^ ^]^ Although direct experimental evidence for enhanced water dissociation is limited, indirect insights can be gained from local pH measurements, often obtained by in situ Raman or FTIR techniques. For instance, the higher local pH on a MgAl‐LDH‐modified Cu surface, compared to bare Cu, provides evidence for the role of hydroxides in proton management during eCO_2_RR.^[^ ^417^ ^]^ This pH shift, derived from the relative ratios of [HCO_3_^−^] to [CO_3_^2−^] from is situ Raman spectra (**Figure** **15**a_1_,a_2_), highlights the importance of hydroxide‐modified Cu catalysts in stabilizing ^*^H‐containing intermediates and promoting C_2+_ product formation.^[^ ^418^, ^419^, ^420^, ^421^, ^422^ ^]^ However, common metal hydroxides like Ni, Co, and Fe are less frequently studied in literature, possibly due to their tendency to promote the HER, despite their strong water dissociation capabilities.

Among metal oxides, CeO_2_ is widely studied for its intrinsic ability to modulate the electronic structure of Cu, thereby improving C_2+_ product selectivity. Recent research by Han et al., suggests that this improvement may result from the role of CeO_2_ in regulating ^*^H coverage.^[^ ^423^ ^]^ Their work shows that CeO_2_ accelerates water splitting, enhancing protonation of ^*^CO to ^*^CHO on Cu sites and optimizing coupling pathways that lead to ^*^CHO─^*^CHO, rather than ^*^COH─^*^COH, ^*^CO─^*^CO, or ^*^CHO─^*^CO.^[^ ^423^ ^]^ Similarly, CuAl_2_O_4_ in a CuAl_2_O_4_/CuO catalyst increases local ^*^H coverage, facilitating important ^*^HCCOH protonation and favoring C_2_H_5_OH formation.^[^ ^112^ ^]^ Other metal oxides incorporated by Cu‐based catalysts, such as SiO_2_ accelerate intermediate protonation,^[^ ^69^, ^424^, ^425^ ^]^ VO_2_ increases surface proton coverage for ^*^CHCOH formation,^[^ ^426^ ^]^ and BaO promotes the formation of ^*^CHCHOH intermediates through intermediates protonation,^[^ ^427^ ^]^ demonstrating the effectiveness of ^*^H optimization by these metal oxides.

In short, oxides and hydroxides exhibit excellent capabilities for cleaving water and providing ^*^H in neutral and alkaline electrolytes. However, the rapid provision of ^*^H may lead to undesirable HER, which needs careful consideration when using them. In addition, balancing the exposure and distribution of hydroxide/oxide active sites with Cu active sites remains crucial to constructing these composite catalysts. Future research should focus on optimizing these factors to enhance the selectivity and efficiency of Cu‐based catalysts for eCO_2_RR.

#### 4.2.3 Organic Complex Linking

The use of organic linkers to regulate active *H has been extensively investigated due to their effectiveness in enhancing product selectivity. This involves incorporating ligands with well‐defined head and tail functional groups into Cu‐based systems, which can act as active sites to fine‐tune intermediate adsorption configurations or modulate the electronic state of Cu sites through electrostatic interactions. These modifications allow for precise control over the reaction pathway, guiding it toward desired products. In the context of intermediate protonation, these functional groups are crucial in controlling the movement of interfacial ^*^H species, significantly influencing the overall reaction course. By strategically manipulating the interaction between organic linkers and catalytic sites, this method offers a viable approach for steering the eCO_2_RR process toward specific outcomes.

Hydrogen bonding is essential in the function of organic linkers, significantly affecting their behavior and interactions with catalytic sites.^[^ ^49^, ^114^, ^117^, ^428^ ^]^ For instance, hydrogen bonding between key intermediates and functional groups, such as ^*^CHO and the ─NH^3+^ ends of zwitterionic glycine,^[^ ^429^ ^] *^CHO and the N─H group of 3,5‐diamino‐1,2,4‐triazole,^[^ ^117^ ^]^ or ^*^COH and ─OH in tannic acid,^[^ ^430^ ^]^ has been shown to stabilize protonated intermediates, promoting subsequent C─C bond formation on Cu‐based catalysts. A recent study demonstrated that in a polydimethylsiloxane (PDMS)‐modified Cu catalyst, an increase in bias potential strengthens intermolecular hydrogen bonds at the interface between water and the catalyst, resulting in longer metal–H distances.^[^ ^431^ ^]^ This optimization of ^*^H coverage on Cu sites not only suppresses HER but also facilitates the protonation of ^*^CO intermediates, enhancing ^*^CO─^*^COH dimerization (Figure 15b). This underscores the importance of regulating hydrogen bonding interactions as a means of modulating intermediate protonation, which can alter C─C coupling modes and influence product selectivity.

Organic linkers can also modify the properties of neighboring Cu sites through interactions between the metal and the linker. Theoretical calculations suggest that amino‐modified Cu catalysts, with −NH_2_ groups, lower the free energy barrier for converting ^*^CO to ^*^CHO by inducing a positively charged state in Cu sites.^[^ ^432^ ^]^ This explains the presence of ^*^COOH and ^*^CHO intermediates during eCO_2_RR. Additionally, bridged Cu sites, such as those formed with pyrazolate and benzotriazole, promote ^*^CO protonation and the formation of ^*^COH or ^*^CHO intermediates, facilitating subsequent dimerization that leads to the production of C_2_H_4_.^[^ ^64^, ^77^ ^]^

Additionally, a recent study revealed that dual Cu sites with a longer interatomic distance (Cu‐I‐Cu) favor the production of C_2_H_4_, while those with a shorter distance (Cu‐Cl‐Cu) favor C_2_H_5_OH production.^[^ ^433^ ^]^ In situ ATR‐SEIRAS and Raman scattering analyses indicated that long‐distance Cu─Cu dual sites can lower the reaction energy barriers for ^*^CO protonation and C─C coupling, while accelerating the deoxygenation of *CH_2_CHO, emphasizing the importance of optimizing ^*^H adsorption through careful control of site distances.

Certain organic groups can also act as ^*^H generators or relays, influencing intermediates protonation and subsequent C─C coupling in eCO_2_RR. Che et al., have demonstrated that an amine group in an aminothiolate ligand‐coated Cu catalyst can produce protonated ^*^COH intermediates, which then couple with other ^*^C_1_ intermediates at Cu sites to stabilize key ^*^COCOH intermediates.^[^ ^72^ ^]^ This highlights the functional role of amine group modification in catalysis. Similar effects are also observed on alkanethiol‐modified Cu catalysts.^[^ ^434^ ^]^ Peters et al., have reported a Cu catalyst modified by an electrodeposited film derived from the N‐tolyl pyridinium additive, and proton transport is optimized to favor C_2+_ products in acidic media.^[^ ^435^ ^]^ In a molecular Cu‐based crystal formulated as [Cu_3_(HBtz)_3_(Btz)Cl_2_] (CuBtz), uncoordinated nitrogen atoms of benzotriazolates near Cu sites can serve as proton relays. These atoms, in cooperation with dicopper(I) sites, facility protonation of ^*^CO intermediates and subsequent ^*^CO–^*^CHO coupling, resulting in a high FE of 73.7% toward C_2+_ products, as illustrated in Figure 15c.^[^ ^436^ ^]^ In fact, literature also suggests that certain molecular linkers containing ions like S, F, I, and N‐doped graphene quantum dots can promote the dissociation of H_2_O to ^*^H species and deliver them during eCO_2_RR.^[^ ^84^, ^101^, ^231^, ^437^, ^438^, ^439^ ^]^ This process aids in the protonation of ^*^CO to ^*^CHO, thereby altering the C─C coupling modes to influence certain product selectivity. However, these hypotheses are based on DFT predictions, with direct experimental validation still lacking.

Overall, by incorporating well‐defined functional groups, organic linkers can fine‐tune the adsorption configurations of intermediates, either by supplying protons or modulating the electronic state of Cu sites, thereby steering reaction pathways toward desired products. Hydrogen bonding interactions are key in stabilizing intermediates and facilitating C─C bond formation alongside the protonation process, while bonded specific organic groups can act as ^*^H generators or relays, further influencing the protonation of intermediates and C─C coupling mode. These works highlight the diverse mechanisms through which organic groups and molecular linkers can modulate catalytic pathways, ultimately enhancing the C_2+_ product efficiency. Future research should focus on exploring a broader range of organic linkers with various functional groups. Appropriate construction of these functional groups and optimizing the interactions may significantly improve the selectivity of target products in eCO_2_RR. Additionally, advanced characterization techniques and computational modeling will offer deeper insights into the mechanistic roles of these linkers.

## 5 Maintaining Stability: Evolution Management

Although fruitful advances have been achieved in enhancing the C─C coupling and improving the selectivity for C_2+_ products, the practical application of Cu‐based catalysts for eCO_2_RR is often hindered by stability issues. Catalyst deactivation is typically categorized as the loss, blockage, or modification of catalyst components.^[^ ^440^, ^441^ ^]^ In the case of Cu‐based catalysts in eCO_2_RR, deactivation commonly occurs due to catalyst evolution processes such as leaching or changes in the active sites that affect valence states, morphology, and composition, leading to a gradual decline in catalytic efficiency.^[^ ^441^ ^]^ Prolonged electrochemical reactions further exacerbate these challenges, making catalyst stability a critical factor in maintaining performance. As a result, maintaining catalytic activity is essential for ensuring the durability and economic feasibility of Cu‐based catalysts in eCO_2_RR, particularly when the challenge of preserving C_2+_ product selectivity is considered. Recently, researchers have increasingly recognized the importance of maintaining catalyst stability and are actively addressing these challenges through innovative design strategies. This section outlines two key strategies for stabilizing catalysts: preserving charged Cu species and managing material evolution, aiming to summarize recent advances in Cu‐based catalysts design for long‐term activity maintenance toward C_2+_ product generation.

### 5.1 Preservation of Charged Cu Species

Despite the historical focus on Cu^0^ as the primary active species in eCO_2_RR, highlighted by the Pourbaix diagram of Cu, which indicates Cu^0^ species as the most stable under cathodic potential,^[^ ^442^, ^443^ ^]^ recent investigations have increasingly emphasized the significance of Cu^δ+^ species (δ >0) in promoting the selectivity toward C_2+_ products.^[^ ^6^, ^34^ ^]^ OD‐Cu catalysts, in particular, have consistently demonstrated distinct reactivity and product selectivity compared to metallic Cu, especially in terms of enhanced C─C coupling and higher selectivity for C_2+_ products. The presence of Cu^δ+^ species introduces a variety of active sites with different oxidation states, imparting diverse electronic and structural properties that influence interactions with reaction intermediates. By modulating the binding strength of key intermediates, Cu^δ+^ species contribute to their stabilization, fostering favorable conditions for C─C coupling and facilitating multi‐step PCET processes. Back in 2012, Kanan et al., discovered that pre‐oxidation of Cu substantially enhances its intrinsic catalytic properties for C_2+_ product formation.^[^ ^444^ ^]^ Later computational studies conducted by Goddard et al., have revealed that the residual Cu^+^ species generated by pre‐oxidation can synergistically interact with Cu^0^ atoms to enhance C_2+_ production.^[^ ^445^ ^]^ The synergy, achieved by facilitating the activation of CO_2_ and C─C coupling on Cu^+^‐Cu^0^ pairs, has been corroborated by subsequent studies.^[^ ^208^, ^251^, ^252^, ^297^, ^446^, ^447^ ^]^ Similarly, Francesca et al., have demonstrated that pure Cu without subsurface oxygen produces only H_2_,^[^ ^448^ ^]^ and Chen et al., have recently proposed a descriptor, (sub)surface oxygenated degree (κ), to quantify surface oxygen in Cu‐based catalysts, revealing a strong correlation between κ and ^*^CO coupling efficiency.^[^ ^449^ ^]^ Additionally, Qiao et al., through integrating computational, AI clustering, and experimental approaches, further confirmed that oxidized Cu surfaces significantly enhance C─C coupling.^[^ ^450^ ^]^ Therefore, the existence of Cu^δ+^ species in various catalysts has become a subject of intense discussion and focus within the field.^[^ ^17^, ^149^, ^180^, ^203^, ^208^, ^245^, ^251^, ^252^, ^297^, ^310^, ^446^, ^451^, ^452^, ^453^, ^454^, ^455^, ^456^, ^457^, ^458^, ^459^, ^460^, ^461^, ^462^, ^463^, ^464^ ^]^ However, despite the recognized importance of Cu^δ+^ species in C_2+_ production, these species are thermodynamically unstable and prone to reduction under eCO_2_RR conditions. Stabilizing Cu^δ+^ species, especially at industrial current densities with strong bias voltages, is a formidable challenge. This has become a major research focus in the quest to improve catalyst stability and efficiency in eCO_2_RR toward C_2+_ products.

Over the past decade, various approaches, including elemental incorporation,^[^ ^90^, ^330^, ^454^, ^465^, ^466^, ^467^ ^]^ microenvironmental regulation,^[^ ^452^, ^468^, ^469^ ^]^ and organic ligand stabilization^[^ ^316^, ^453^, ^470^, ^471^, ^472^ ^]^ have been explored in maintaining catalytic activity by stabilizing Cu^δ+^ species. Nevertheless, the short stability period of Cu^δ+^ species and the unpredictability of products during eCO_2_RR highlight the need for more robust stabilization methods and mechanisms. In response, recent emerging strategies such as defect engineering, Cu‐based compound utilization, molecular modification, inorganic elements and compounds incorporation, and spatial structure engineering have shown promise in advancing exploration and innovation within this critical area. This subsection reviews these advances, offering insights into the protection mechanisms of Cu^δ+^ species, with the goal of addressing the challenge of maintaining catalytic activity in Cu‐based catalysts for eCO_2_RR toward C_2+_ product.^[^ ^251^, ^252^ ^]^

#### 5.1.1 Defect Engineering

**Figure 16:**
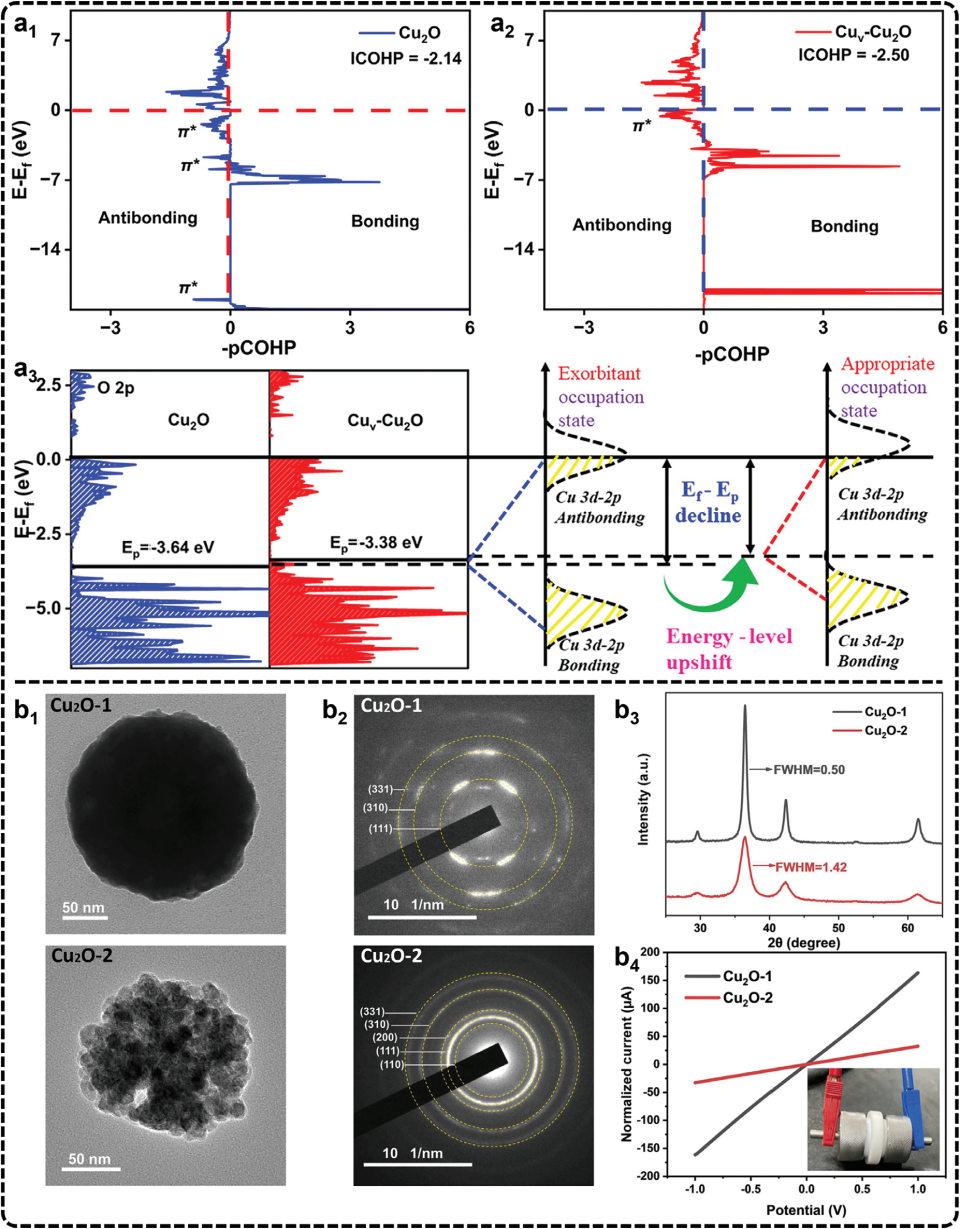
Crystal orbital Hamilton population (COHP) for Cu^+^ 3d–O 2*p* interaction in a_1_) Cu_2_O and a_2_) Cu_v_‐Cu_2_O, respectively, and a_3_) PDOS for the O 2*p* orbital in Cu_2_O and Cu_v_‐Cu_2_O and the schematic illustration of antibonding and bonding orbital energy level.^[^ ^473^ ^]^ The results suggest that the introduction of Cu defects into Cu_2_O markedly elevates the O 2*p* energy level, effectively depressing the antibonding orbital occupancy near E_f_ and thus enhancing the Cu─O bond strength. Augmenting this bond strength correlates with a truncated bond length, thus potentially bolstering the Cu^+^ stability. Adapted with permission.^[^ ^473^ ^]^ Copyright 2024, American Chemical Society. b_1_) TEM images, b_2_) SAED patterns, and b_3_) XRD patterns showing the grain sizes difference; b_4_) Electronic conductivity (insert showing the measuring setup) showing that Cu_2_O‐2 with abundant grain boundary defects exhibits lower electron conductivity. Adapted with permission.^[^ ^478^ ^]^ Copyright 2023, Elsevier.

Defect engineering has emerged as a powerful approach for tailoring the properties of Cu‐based catalysts to enhance their performance in eCO_2_RR. By intentionally introducing defects, such as vacancies and grain boundaries, it is possible to modify the catalytic behavior of Cu‐based materials. Vacancies and grain boundaries can serve as active sites, promoting CO_2_ adsorption, activation, and conversion. Additionally, these defects can also change the coordination environment and create localized electronic structures, which influence the binding energies of intermediates and steer the reaction toward specific pathways. However, the behavior of these defects under the strongly reducing conditions of eCO_2_RR remains and is not fully understood, as the high bias voltage may cause the mobilization of both atoms and defects within the catalysts. One intriguing aspect of defect engineering in Cu‐based catalysts is its potential in stabilizing Cu^δ+^ species, which have been increasingly recognized for their importance in promoting C_2+_ product formation during eCO_2_RR. In recent, cationic Cu vacancies and anionic O vacancies have been extensively studied for their role in this process. Yan et al., have employed DFT to elucidate the relationship between antibonding occupancy states and the stability of Cu^δ+^ active sites.^[^ ^473^ ^]^ They found that the introduction of cationic Cu vacancies weakened antibonding occupancy states at the Fermi level (**Figure** **16**a_1_,a_2_). This weakening, in turn, enhances the interaction between Cu and O (Cu─O) and thereby stabilizes Cu^δ+^ sites (Figure 16a_3_). Experimentally, they're a series of ex situ and in situ characterizations that have confirmed the stability of Cu^δ+^ species during eCO_2_RR and highlight the ability of Cu^δ+^ species to suppress the HER while simultaneously promoting sustained CO_2_ activation and subsequent C─C coupling. On the other hand, the role of O vacancies is more straightforward to understand, as they can modulate the electronic structure around Cu active sites and influence their valence state. In ultrathin 2D Cu_2_O nanosheets, for example, O vacancies are proven to prevent the full reduction of Cu to metallic form, enabling continuous C─C coupling and extending the operational lifespan of the catalyst.^[^ ^312^ ^]^

In addition, grain boundaries, with their distinct local environments, may also provide favorable conditions for the sustained presence of Cu^δ+^ species. Kanan's group pioneered research on the role of grain boundaries in CO_2_ and CO electroreduction, highlighting their impact on catalyst performance.^[^ ^6^, ^474^, ^475^ ^]^ Recently, several studies have provided fresh insights. For instance, Yang et al., introduced an oxide‐derived nanocubic Cu catalyst enriched with grain boundaries.^[^ ^476^ ^]^ These grain boundaries promote the adsorption of hydroxyl (^*^OH) species, generating a locally alkaline environment that stabilizes dual‐phase Cu(I) and Cu(0) sites throughout the operating potential range. As a result, the catalyst maintains high selectivity for C_2_H_5_OH over 15 h of continuous electrolysis. Moreover, Cu‐based catalysts with varying degrees of phase mixing (Cu + Cu_2_O + CuO) have been found to significantly improve both selectivity and stability in the formation of C_2+_ products, attributed to the higher surface Cu^+^ content facilitated by increased phase mixing or the presence of more abundant grain boundaries.^[^ ^281^, ^303^, ^477^ ^]^ However, there are still some uncertainties surrounding the mechanisms due to the limited evidence on how these mixed phases mitigate the reduction of positively charged Cu during eCO_2_RR. A potential explanation comes from the grain refinement strategy proposed by Wu et al.^[^ ^478^ ^]^ Their approach utilized Cu_2_O nanospheres with abundant grain boundary defects, which exhibit lower electron conductivity compared to nanospheres with fewer grain boundaries. Figure 16b shows the TEM, SAED, and XRD comparison of Cu_2_O nanospheres with varying grain boundaries, attributed to differences in grain sizes, along with results from simple electronic conductivity measurements. This reduced electron conductivity is believed to play a key role in mitigating the complete reduction of Cu_2_O during eCO_2_RR. Consequently, the lifespan of Cu^+^ species is extended under high current densities, contributing to enhanced stability alongside excellent selectivity for C_2+_ products. However, in situ Raman monitoring revealed that Cu^+^ species eventually disappeared after 50 min, indicating that their reduction might be influenced by reduction kinetics. Although grain boundaries slow the reduction of Cu^+^, they do not completely prevent it. Despite this limitation, this method offers a promising, straightforward strategy for suppressing Cu^+^ reduction and could be applied to other Cu‐based catalyst systems.

Overall, the construction of defects, such as the introduction of vacancies or grain boundaries, represents a promising strategy for stabilizing Cu^δ+^ species to maintain the catalytic performance of Cu‐based catalysts in eCO_2_RR. By tailoring the structural and electronic properties of catalysts through the deliberate introduction of defects, researchers can achieve both improved selectivity and long‐period operation stability, advancing the development of more efficient catalysts for sustainable CO_2_ conversion. However, the kinetic issue of Cu^+^ reduction remains a critical issue that requires attention in future studies. Many previous reports often lack detailed time‐resolved analyses in in situ detection or are limited by short experimental durations, potentially overlooking the temporal dynamics of Cu^+^ reduction. For instance, in polycrystalline CuO or Cu_2_O catalysts, Cu^+^ sites are typically reduced within 1 h of reaction, despite their improved C_2+_ product selectivity compared to single‐crystalline catalysts.^[^ ^207^, ^463^, ^478^ ^]^ Since the oxidation state of Cu changes with applied potentials and reaction durations, future research should prioritize time‐varying detection methods to better capture the transient behavior of Cu^+^ species during eCO_2_RR.^[^ ^453^, ^479^ ^]^ This is especially important as Cu+ signals may only be present for brief periods in some cases, while in others, they may disappear even sooner. considering that Cu^+^ signals may only persist for a short time in some cases, while others detect them for shorter durations.

#### 5.1.2 Cu‐Based Compounds Utilization

**Figure 17:**
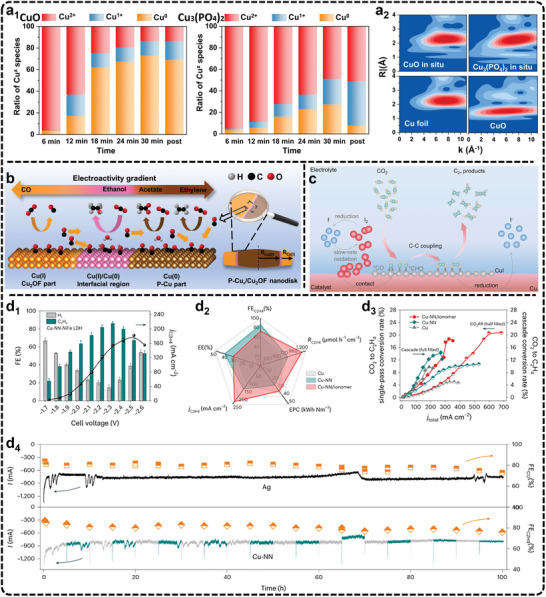
a_1_) Calculated ratios of Cu oxidation state in CuO and Cu_3_(PO_4_)_2_ catalysts from in situ spectra linear combination fitting with respect to time during 30 min of reaction at −1.45 V, reveals that the transition between Cu^2+^ and Cu° for CuO is more rapid, while the reduction for Cu_3_(PO_4_)_2_ is slower; a_2_) the Morlet WT of the *k* ^3^‐weighted operando EXAFS data for the Cu_3_(PO_4_)_2_ and CuO samples with standard Cu foil and CuO powder as controls suggest the stability of Cu^2+^ coexistence of Cu─O and Cu─Cu bond in Cu_3_(PO_4_)_2_ during eCO_2_RR. Adapted under terms of the CC‐BY license.^[^ ^481^ ^]^ Copyright 2023, Zhang et al. b) Schematic illustrations of the reaction mechanisms on the three different surficial sites on P‐Cu_x_/Cu_2_OF. The relative thicknesses of Cu(I) (R_Cu(I)_) and Cu(0) (R_Cu(0)_) layers vary with the Cu(I)/Cu(0) molar ratio, and only when both Cu(I) and Cu(0) have appropriate thicknesses can the C_2_H_5_OH‐activity of the interface region become optimized. Adapted with permission.^[^ ^484^ ^]^ Copyright 2024, Elsevier. c) Schematic mechanism of eCO_2_RR on Cu in an I_2_ additive electrolyte. Reproduced with permission.^[^ ^493^ ^]^ Copyright 2024, American Chemical Society. d) Performance in cascade MEA cells with NiFe LDH and Cu‐NN as anode and cathode catalysts, including the d_1_) FE for C_2_H_4_ obtained using CO_2_ as feed (The grey and deep green bar charts represent the FE of H_2_ and C_2_H_4_, whereas the black solid ball line curve represents the *j*_C2H4_ at the same cell voltages), d_2_) comparison of different performances of the Cu‐*X* electrodes: FE, energy efficiency (EE), specific current density (*j*_C2H4_), energy power consumption (EPC) and formation rate (*R*) of C_2_H_4_, d_3_) the comparison of the CO_2_‐to‐C_2_H_4_ single‐pass conversion measured for a single MEA cell (half‐filled circle, prismatic and triangle) and for the cascade flow process (full‐filled circle, prismatic and triangle), and the d_4_) stability of Ag and Cu‐NN catalysts in MEA cells (the half‐filled square and prismatic represent the FE of CO and C_2_H_4_). The error bars correspond to the standard deviation of three independent measurements; data are presented as mean ± s.d. Adapted with permission.^[^ ^94^ ^]^ Copyright 2024, Springer Nature.

Cu‐based compounds encompass a wide range of well‐defined materials with varying electronic properties and chemical compositions, including the commonly used hydroxide, oxide, and halide. These compounds often feature Cu ions in specific oxidation states, such as Cu^+^ and Cu^2+^. Harnessing the inherent electronic states of Cu in these compounds offers a promising avenue for stabilizing Cu^+^ species during the eCO_2_RR. By cautiously modifying Cu‐based compounds, researchers may tailor the local environment around Cu ions to alter the electronic structure of Cu sites and then simultaneously influence the adsorption properties of intermediates and inhibit the loss of active Cu^δ+^ sites, ultimately improving the catalytic performance toward the C_2+_ products generation. For example, Cu hydroxide catalysts have been reported to reduce to metallic Cu under eCO_2_RR within 1 h.^[^ ^207^, ^479^, ^480^ ^]^ However, when highly negative reduction potentials are applied in the initial period, certain residual Cu^δ+^ species can persist for over 40 h, facilitating continued C─C coupling and C_2+_ products generation.^[^ ^455^ ^]^ This demonstrates the potential benefit of electrochemical pretreatment, which should not be overlooked during catalyst assessments, as it can enhance corrosion resistance through the rapid migration of internal atoms. Ingenious utilization of such pretreat methods may yield unexpected harvests. A recent report noticed that Cu phosphate‐based compounds can inherently resist reduction during being eCO_2_RR.^[^ ^481^ ^]^ **Figure** **17**a shows the comparison of Cu oxidation states over time, along with in situ EXAFS data for CuO and Cu_3_(PO_4_)_2_ catalysts, revealing the formation of stable Cu^0^/Cu^2+^ interfaces in Cu_3_(PO_4_)_2_ during catalysis. Attributed to this feature, the catalyst can achieve a remarkable FE of 90.9% toward C_2+_ products with superior stability, emphasizing the importance of stabilizing Cu^0^/Cu^2+^ interfaces for sustained catalytic performance. However, the study only tested for 30 min, leaving open the question of whether this catalyst can fully prevent reduction or merely delay it over short durations.

Halide ions in cuprous halide compounds play a significant role in protecting charged Cu species. These ions have been observed to undergo partial leaching and reabsorption during eCO_2_RR, leading to dynamic reconstruction of Cu structures and oxidation states.^[^ ^470^, ^482^, ^483^ ^]^ The resulting residual Cu^δ+^ species formed and stabilized during this in situ reconstruction have been emphasized in determining reaction pathways and sustaining activity. In a study on halide‐modified Cu catalysts, the Cu oxidation state stabilizes between 0 and 1 during eCO_2_RR, with the valence state increasing proportionally to the electronegativity of the halogen.^[^ ^101^ ^]^ Among them, F‐modified Cu catalysts demonstrate enhanced stabilization of higher valence state of Cu species, which is vital for maintaining catalytic performance. Recently, these concepts have been extended further in the design of a disk‐like catalyst composed of an F‐stabilized Cu^+^ shell with a Cu° core (P‐Cu_x_/Cu_2_OF),^[^ ^484^ ^]^ as well as a plasma fluorinated Cu^+^ catalyst,^[^ ^485^ ^]^ both demonstrating continuous generation of C_2+_ products due to the stabilization of Cu^+^ sites. Figure 17b illustrates the reaction mechanism across three active regions of P‐Cu_x_/Cu_2_OF, visualizing the relationship between the Cu(I)/Cu(0) molar ratio and C_2_H_5_OH activity.

However, despite these achievements in stabilizing Cu^δ+^ species, conflicting findings on the impact of halide ions have emerged. For example, studies on electrolyte anion additives reveal a trend of C_2+_ product selectivity increasing from Cl^−^ to Br^−^ to I^−^.^[^ ^486^, ^487^ ^]^ This trend corresponds to the adsorption behavior of halide ions on Cu surfaces, with I^−^ exhibiting the most favorable adsorption, as determined by DFT studies.^[^ ^488^ ^]^ These findings introduce a more complex scenario in understanding how halide ions impact Cu‐based catalysts, highlighting the need for comprehensive experimental designs and advanced in situ observation techniques to elucidate their effects. Consequently, the stabilization of Cu^δ+^ species is not limited to F^−^ ions. Recently, some ingeniously designed Cu halides have been reported to successfully construct stable Cu^+^ sites to facilitate the sustained C_2+_ product generation. For instance, Bao et al., have designed a Cu‐CuI composite catalyst and achieved a high and stable C_2+_ partial current density.^[^ ^489^ ^]^ In this case, although the catalyst undergoes reconstruction under operational conditions in the initial period, a portion of Cu^+^ species is retained due to the adsorption of iodine species. This retention helps explain the later sustained selectivity, consistent with previous findings of Beatriz et al.^[^ ^290^, ^470^ ^]^ Similar observations have been reported with AgI‐incorporated CuO catalysts, where iodine leaching creates a localized iodine‐rich environment that restrains CuO self‐reduction during eCO_2_RR.^[^ ^490^ ^]^ This stabilization results in active Cu^0^/Cu^+^ species capable of sustained producing C_2+_ products with an FE of ≈68.9%. Additional examples include the partial leaching of Cl ions from Cu_4_(OH)_6_FCl nanosheets^[^ ^491^ ^]^ and Br ions from dodecanethiol‐modified CuBr catalysts^[^ ^492^ ^]^ during eCO_2_RR, both of which were proved to produce stabilized Cu^0^‐Cu^+^ sites, prolonging operational periods and highlighting the effectiveness of such protective mechanisms of halide elements. Moreover, direct halogen addition to the electrolyte may offer a similar stabilizing effect. Qiao et al., have introduced this innovative approach by adding excess I_2_ into the electrolyte during eCO_2_RR.^[^ ^493^ ^]^ This addition facilitated the in situ formation of CuI species through slow‐rate oxidation, resulting in the stabilization of a Cu^0^/Cu^+^ interface in an acidic environment, as depicted in Figure 17c. The stabilized interface significantly improved both FE and stability for C_2+_ products in low K^+^ concentration electrolytes.

Overall, Cu‐based compounds offer a versatile platform for stabilizing Cu^+^ species to improve the stability of eCO_2_RR toward C_2+_ products. By leveraging the intrinsic electronic properties of Cu and employing targeted treatment, it is possible to develop advanced catalysts capable of achieving sustained high selectivity for valuable C_2+_ products. In particular, Cu‐based halides have emerged as promising candidates for preserving the desirable electronic state of Cu, meriting further exploration. Nevertheless, maintaining the long‐term stability of Cu‐based compounds under strong reducing conditions remains a significant challenge, as most reported stability durations in the literature do not exceed 100 h. This presents a key area for future research.

#### 5.1.3 Molecular Modification

The adoption of molecular modification on Cu‐based electrodes holds significant implications for inhibiting the reduction of Cu^δ+^ sites during eCO_2_RR. Molecular modifications can either create a protective layer or alter the electronic structure of the Cu‐based electrode. For instance, the attachment of organic molecules, polymers, or ligands can form a physical barrier on the Cu surface, shielding it from direct exposure to the reducing environment. By limiting interactions between the surface and the electrolyte, this layer can slow down undesirable side reactions and material degradation. Furthermore, the molecular layer can be tailored to permit specific species, such as ^*^CO or CO_2_, to interact with Cu^δ+^ sites while restricting the access of protons or other aggressive species that may reduce Cu^δ+^ to Cu^0^, thereby preserving the crucial Cu^δ+^ oxidation state needed for C_2+_ product formation. Additionally, molecular modifiers can electronically couple with the Cu surface, altering the local electronic environment. Electron‐donating or electron‐withdrawing groups within the modifiers may stabilize Cu^δ+^ by making its reduction to Cu^0^ less energetically favorable under reaction condition, which reduces the risk of site degradation and maintains the catalytic activity.

One promising approach involves using thiol end groups, which are suggested to bind stably to Cu even under electrochemical reducing conditions.^[^ ^67^, ^494^, ^495^ ^]^ Additionally, sulfur, acting as a Lewis base, may have more opportunities to interact with neighboring intermediates, influencing catalytic pathways and material stability. In a study by Masuda et al., methanethiol monolayers‐modified Cu electrodes have been shown to undergo surface reconstruction while the Cu⁺ species is formed due to the electrochemical reduction of methanethiol monolayer at negative potentials.^[^ ^496^ ^]^ The stable Cu^+^ species, in conjunction with metallic Cu, effectively promote ^*^CO─^*^CO dimerization to enhance the sustained generation of C_2+_ products. Recently, Voiry et al., have proposed a precise molecular doping strategy using various substituted aryl diazonium salts as electrophilic reagents to modulate Cu catalysts for enhanced selectivity and stability.^[^ ^94^ ^]^ Supported by DFT calculations, their work shows that the surface oxidation state of Cu (δ+, 0 < δ < 1) can be modulated via functionalization by these different groups. In situ spectral data validates the low energy formation of ^*^CH_2_CH intermediates on specific Cu^0.26+^ sites in a designated Cu‐NN catalyst, while the electrochemical results in cascade membrane electrode assembly (MEA) cells illustrate the superior FE, partial current density, energy efficiency, and stable operational situations over 100 h (Figure 17d), underscoring the importance of Cu valance control in maintaining stable activity. Additionally, 2,3,7,8‐tetraaminophenazine‐1,4,6,9‐tetraone,^[^ ^497^ ^]^ phenyl‐1H‐1,2,3‐triazole derivatives,^[^ ^318^ ^]^ and 2‐mercapto‐1‐methylimidazole^[^ ^231^ ^]^ coordinated with Cu have recently been shown to maintain active Cu^δ+^ species during eCO_2_RR and support sustained C_2+_ product generation. Notably, the 2,3,7,8‐tetraaminophenazine‐1,4,6,9‐tetraone coordinated Cu catalyst has exceptionally stable Cu^+^‐Cu^2+^ sites during catalysis, a feature that is generally difficult to achieve. Similar strategies to stabilize Cu^δ+^ species have been achieved using tannic acid molecules‐modified Cu (TA‐Cu) catalysts,^[^ ^430^ ^]^ sodium citrate‐modified Cu_2_O catalysts,^[^ ^498^ ^]^ and SiO_2_‐assisted silane coupling agent‐incorporated Cu_2_O catalysts.^[^ ^499^ ^]^ The TA‐Cu catalysts featuring Cu particles and abundant hydroxyl species stabilize the Cu^δ+^/Cu^0^ interface during eCO_2_RR, as confirmed by in situ XAS and Raman spectroscopy. Similarly, sodium citrate‐modified Cu catalysts leverage ─COOH coordination to induce electron transfer from Cu_2_O to citrate anions, stabilizing Cu^+^ species under reduction conditions. Both reports emphasize the significance of functional groups in stabilizing the Cu^δ+^ species to maintain catalytic selectivity.

However, behind the experimental observations, the underlying mechanisms by which these functional groups partially control the valence state of Cu are not thoroughly discussed. One potential mechanism is the strong coordination of Cu by functional groups. In a benzimidazole‐modified Cu electrode, strong Cu‐imidazole coordination bonds have been shown to provide remarkable stability under reduction conditions, enabling the sustained presence of Cu^+^ species for continuous acetate electrosynthesis over 190 h.^[^ ^500^ ^]^ Another example is a cyanamide‐coordinated isolated Cu framework catalyst.^[^ ^323^ ^]^ The cyanamide anions ([NCN]^2−^) prevent the self‐reduction of Cu^+^ by delocalizing d‐electrons and enhancing electron conductivity through π‐electron flow, maintaining a stable Cu^0^/Cu^+^ balance during 15 h of stable operation. These works suggest that specific ligands can coordinate with Cu atoms, modifying the d‐orbital electron configuration, which in turn alters the electronic structure of Cu to a stable state. This may help facilitate desired reaction pathways while contributing to the long‐term operation of the catalyst.

In summary, the application of molecular modification represents a promising avenue for tailoring Cu‐based electrodes to maintain the active sites. Most molecular modifiers can lead to localized charge redistribution on the Cu surface, creating regions of higher or lower electron density. This redistribution can affect how Cu^δ+^ interacts with reactants and intermediates, stabilizing the partially oxidized state and preserving its reactivity, which is crucial to promoting C_2+_ products in eCO_2_RR. However, further exploration is needed to understand the intricate mechanisms underlying molecular modifications and their impact on both Cu sites and reaction intermediates. Additionally, efforts to develop novel molecular doping strategies and explore alternative molecular modifiers have the potential to advance the field and address stability challenges associated with eCO_2_RR.

#### 5.1.4 Inorganic Elements and Compounds Incorporation

**Figure 18:**
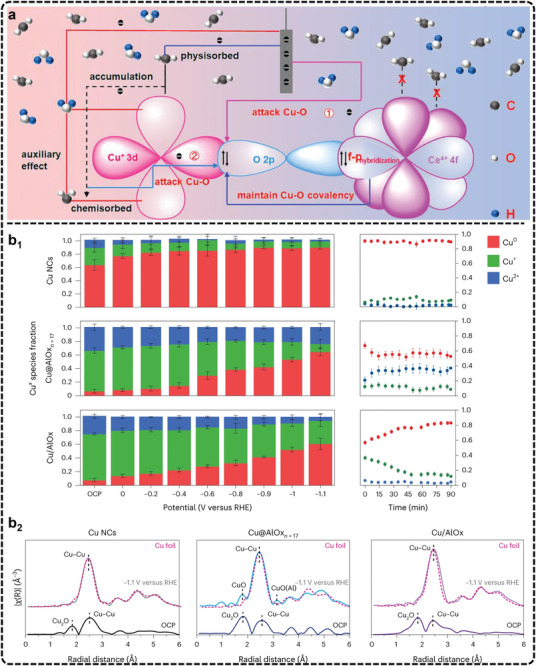
a) Origin of Cu^+^ deactivation and a schematic diagram illustrating the coupling of the high‐order orbitals 4f and 2p in Ce‐Cu_2_O that maintains Cu─O covalency and protects the Cu^+^ site from electron attack during the eCO_2_RR. Reproduced with permission.^[^ ^277^ ^]^ Copyright 2023, American Chemical Society. b_1_) Evolution of Cu*^x^* species fraction as a function of the applied potential and time at −1.1 V versus RHE, extracted from linear combination analysis of the XANES spectra for Cu NCs; b_2_) EXAFS data at OCP (dark color) and at −1.1 V versus RHE (bright color) for Cu NCs (black), Cu@AlO_x_ *_n_*_= 17_ (blue) and Cu/AlO_x_ (purple). The data are the average of Cu fractions extracted from six consecutive XANES spectra, and the error bars are the calculated standard deviation: Cu NCs, Cu@AlO_x_ *_n_*_= 17,_ and Cu/AlO_x_. The fraction of oxidized Cu at OCP is similar in Cu@AlO_x_ *_n_*_= 17_ and Cu/AlO_x_ cases, yet this fraction is much higher compared with that measured for the as‐synthesized Cu NCs. As the cathodic potential is applied, the level of oxidized Cu is reduced for all samples; however, Cu@AlOx*_n_*_= 17_ and Cu/AlO_x_ maintain a substantial fraction of Cu^2+^ and Cu^+^, respectively. Cu@AlO_x_ *_n_*_= 17_ preserves most of the Cu^2+^ during operation over time; on the contrary, Cu/AlO_x_ eventually transforms into metallic Cu with a statistically insignificant fraction of oxidized Cu remaining. EXAFS data confirmed the trends observed in the XANES. Adapted under terms of the CC‐BY license.^[^ ^521^ ^]^ Copyright 2024, Albertini et al.

The introduction of inorganic hetero‐elements or compounds has also demonstrated a compelling impact on stabilizing Cu^δ+^ species during eCO_2_RR. The presence of hetero‐metals can lead to the formation of mixed oxides or complex compounds that enhance the thermal and chemical stability of Cu^δ+^. These heterometals generally engage in metal‐oxygen interactions, adjusting the local electronic structure around Cu^δ+^ species to maintain their partially oxidized state. The introduction of strong electronegative anions serves to directly regulate the Cu oxidation state by creating strong bonds with Cu atoms and pulling electrons away. Heterometals such as Pd, Ce, Ti, Sr, Al, Gd, Sn, and Mg have shown significant effects on maintaining Cu^δ+^ species, potentially acting as electron acceptors due to the strong metal‐oxide interactions during eCO_2_RR conditions.^[^ ^138^, ^277^, ^358^, ^361^, ^464^, ^501^, ^502^, ^503^, ^504^, ^505^, ^506^, ^507^, ^508^ ^]^ These metals can create new metal‐oxygen bonds that alter the local structure and electronic environment around Cu, preventing the collapse of Cu^δ+^ into a neutral Cu^0^ state and preserving the partially oxidized Cu^δ+^ form essential for C_2+_ product generation. For instance, Yan et al., recently revealed the mechanism by which Ce─O interactions protect the Cu oxidation state in eCO_2_RR.^[^ ^277^ ^]^ As shown in **Figure** **18**a, Ce^4+^─O interaction involving an unconventional orbital hybridization near E_f_ based on the high‐order Ce^4+^ 4*f* and O 2*p* orbitals can inhibit lattice oxygen leaching during the CO_2_ activation under eCO_2_RR conditions, thereby stabilizing Cu^+^ species. Likewise, Mg^2+^ in Mg‐modified Cu_x_O nanoparticles stabilizes Cu^+^ sites through strong Mg─O─Cu interactions, suggesting an electron donation effect under reaction conditions, which facilitates the stability of C_2+_ product generation.^[^ ^358^ ^]^ A similar effect is also observed with Sn anchored on Cu_2_O catalysts, which exhibit excellent resistance to Cu^+^ species reduction during the reaction, further highlighting the role of metal‐oxide interactions in preserving Cu^δ+^ species.^[^ ^506^ ^]^

In addition to incorporating highly electronegative anions that bond directly with Cu, certain nonmetals with lower electronegativity have also been effective in stabilizing Cu^δ+^ species. For instance, Hong et al., reported that interstitial carbon‐doped Cu_2_O nanoparticles (C‐Cu_2_O NPs) promote the formation of abundant unsaturated Cu─O bonds.^[^ ^509^ ^]^ This stabilization of Cu^δ+^ species during eCO_2_RR ensures sustained C_2+_ product generation for over 20 h. Additionally, B incorporation has been utilized to tune the ratio of Cu^δ+^ to Cu^0^ active sites by creating strong B─Cu bonds, which help balance selectivity for C_2+_ products and maintain catalytic stability.^[^ ^465^ ^]^ The effectiveness of stabilizing oxidized Cu species through B incorporation has been further supported through electronic structure analysis and theoretical calculations in other reports, underscoring its significance in catalyst design.^[^ ^375^, ^510^, ^511^ ^]^ Similarly, BN‐decorated Cu_2_O nanoparticles have demonstrated the reinforcement of Cu─O bonds, which helps regulate bond energy and improve both selectivity and stability for C_2+_ products.^[^ ^512^ ^]^ Additionally, Hong et al., reported that Cu_2_O nanoparticles doped with interstitial carbon atoms (C‐Cu_2_O NPs) effectively convert CO_2_ into C_2+_ products. The interstitial carbon promotes the C‐Cu_2_O NPs to possess abundant unsaturated Cu─O bonds, leading to a high‐density Cu^δ+^ (0< δ<1) species that can stably exist in eCO_2_RR conditions, leading to a sustained C_2+_ production generation over 20 h.

Oxide compounds possess inherent oxide phases, which can serve as sacrificial layers or passivating layers to stabilize Cu^δ+^ species. These phases may have the capability to accept electrons prior to the Cu^δ+^ sites, effectively shielding them from further reduction or degradation during the reaction, consequently allowing them to remain active for longer periods and thereby enhancing the overall catalytic performance. For example, Sun et al., have demonstrated that incorporating CeO_2_ into CuO stabilizes Cu^+^ species during eCO_2_RR, extending their lifetime over 1 h while improving C_2+_ product generation.^[^ ^513^ ^]^ This study illustrates the broader potential of using oxide materials to form protective environments around Cu^δ+^ sites, particularly in harsh electrochemical conditions. Expanding on this work, other studies have explored the multifaceted roles of CeO_2_ in stabilizing Cu^δ+^ species. CeO_2_‐modified Cu electrodes were found to undergo oxidative corrosion to result in the formation of an active CuO_x_ layer, which contributed to the prolonged stability of the catalytic process.^[^ ^514^ ^]^ Similarly, Cu‐CeO_2_ nanorods were observed to reconstruct into stable nanoclusters containing both Cu^0^ and Cu^+^ species, highlighting the dynamic reorganization capabilities of the material under reaction conditions.^[^ ^515^ ^]^ In a separate study, the facile Ce^3+^/Ce^4+^ redox reactions in CeO_2_‐modified CuS nanoplates were proposed as another mechanism for maintaining Cu^+^ during eCO_2_RR, thereby promoting more stable C_2+_ product formation.^[^ ^516^ ^]^ The dual role of CeO_2_, functioning as both a protective layer and a redox mediator, underscores its effectiveness in stabilizing charged Cu sites, which is critical for enhanced durability and catalytic efficiency. Beyond CeO_2_, other metal oxide incorporations such as BaO_2_, ZrO_2_, Sb_2_O_4_, and Al_2_O_3_, have also been investigated for their potential in stabilizing Cu‐based catalysts during eCO_2_RR toward C_2+_ product generation.^[^ ^427^, ^517^, ^518^, ^519^ ^]^ These oxides are believed to operate in a similar manner, either by forming protective layers or by participating in redox reactions that prevent the reduction of Cu^+^ species. Notably, BaO_2_ and ZrO_2_ have been found to support the retention of Cu^δ+^ species by acting as electron sinks, reducing the likelihood of Cu° formation and thereby sustaining the catalyst activity over prolonged operation. Additionally, through phase and interphase engineering, Cu oxide species in CuO nanoparticles covered Al_2_CuO_4_ nanosheet catalyst have been well retained after eCO_2_RR, highlighting the meaning of phase engineering and Al oxide.^[^ ^364^ ^]^ These studies collectively emphasize the importance and broader potential of metal oxides in engineering stable, high‐performance Cu‐based catalysts for prolonged operation in eCO_2_RR.

Overall, the incorporation of inorganic elements and compounds offers a versatile and effective strategy to stabilize Cu^δ+^ species during eCO_2_RR. One key mechanism is the creation of a stable electronic environment around oxidized Cu sites, which is achieved by adding electron acceptors, enhancing Cu─O bonds, or decorating a protective layer. However, the stability durations reported in the literature remain relatively short. For instance, certain electrocatalysts, such as dodecanethiol‐modified CuBr and BN nanosheets‐decorated Cu_2_O, exhibit prolonged catalytic activity compared to unmodified CuBr and Cu_2_O, lasting over only 15 and 14 h, respectively.^[^ ^492^, ^512^ ^]^ Despite this improvement, it remains uncertain whether these activities can be maintained for longer durations, as the current times reported fall short of industrial requirements. Future research should consider the temporal dynamics of Cu⁺ reduction, as these hetero components may only temporarily delay the reduction of Cu, necessitating further investigation into long‐term stability solutions.

#### 5.1.5 Spatial Structure Engineering

Recent advancements in catalytic stability have emerged from the exploration of core–shell structures and other spatially engineered catalysts, as reviewed by Kawi et al.^[^ ^520^ ^]^ These spatially structured catalysts offer distinct advantages in mitigating activity loss over prolonged reaction durations. For example, with core–shell structures, one of the most reported aspects, encapsulating a catalytically active core within a protective shell provides effective shielding against detrimental environmental conditions, such as corrosive electrolytes or harsh reaction environments brought from bubbles during eCO_2_RR, thereby preserving its catalytic activity and structural integrity. Furthermore, the spatial structure shell may act as a barrier, limiting the diffusion of reactants and intermediates to the internal material to reduce the likelihood of catalyst degradation or deactivation. This confinement of reactants within the shell also promotes efficient catalytic turnover while minimizing side reactions or unwanted byproducts.

The design flexibility offered by spatially structured catalysts allows for precise control over the composition, structure, and even the thickness of the protective shell, thus enabling tailored catalytic performance and enhanced stability. By optimizing these parameters, researchers can fine‐tune the catalytic activity, selectivity, and durability of the catalyst, ensuring optimal performance under specific reaction conditions. Recently, Buonsanti et al., have proposed a novel approach to protect the active Cu sites by designing a Cu catalyst encapsulated with alumina (Cu@AlO_x_).^[^ ^521^ ^]^ This design aims to create electrocatalysts resistant to structural reconstruction during eCO_2_RR. The Cu^x^ species fraction obtained from in situ XAS spectra revealed that the alumina shell effectively locks some Cu surfaces in a reduction‐resistant Cu^2+^ state (Figure 18b), preventing Cu structural reconstruction while preserving the catalyst reactivity over varying voltages and time scales. They have also investigated the stabilization mechanism by varying the shell thickness and coating morphology, highlighting the importance of the Lewis acidity of the shell in preserving the catalyst structure.

The cavity structure of Cu may benefit from limiting the mass transfer while aggregating the intermediates, which plays a critical role in stabilizing active sites through a reverse interaction of intermediates. This phenomenon, where in situ generated carbon intermediates accumulate within Cu nanosized cavities during eCO_2_RR, effectively “masking” the local catalyst surface, has been emphasized in various studies.^[^ ^466^, ^522^, ^523^ ^]^ This localized high concentration of intermediates can help stabilize surface positively charged Cu species by reducing their exposure to harsh reaction conditions. As a result, the continuous generation of C_2+_ products is facilitated, highlighting the importance of both the intermediates and the unique structural features of the catalyst during operation. A recent advancement entails the design of a series of network‐structured catalysts composed of ultrafine Cu_2_O/CuO, Cu_2_O/CuO, Cu/Cu_2_O, Cu_2_O, or CuO nanoparticles, fabricated using a flame spray pyrolysis method.^[^ ^155^ ^]^ These catalysts are designed to confine carbon intermediates during eCO_2_RR, which, in turn, covers the local catalyst surface and stabilizes Cu^+^ species. The Cu^+^ species surviving during the reactions is evidenced by in situ XRD and XPS spectra, highlighting the effectiveness of this strategy in stabilizing Cu^+^ species for generating C_2+_ products.

Additionally, employing MOFs as a protective coating is a viable option due to their porous nature. MOFs are composed of metal ions or clusters coordinated with organic ligands, resulting in a crystalline structure with well‐defined pores and channels. These inherent characteristics render MOFs ideal for encapsulating Cu‐based catalysts and protecting Cu^δ+^ species during eCO_2_RR to facilitate the generation of C_2+_ products.^[^ ^524^, ^525^, ^526^ ^]^ Recent work tends to find new MOF materials to optimize catalytic performance and focus on providing more in situ evidence of the protection mechanism. For instance, Peng et al., have encapsulated Cu_2_O nanocubes in metalloporphyrin frameworks for eCO_2_RR catalysis aimed at C_2+_ products.^[^ ^71^ ^]^ In situ XAS analysis revealed that, under eCO_2_RR conditions, Cu^+^ cations in pristine Cu_2_O nanocubes fully convert to Cu^0^, while the metalloporphyrin framework‐encapsulated Cu_2_O showed minimal changes in Cu oxidation and coordination states. This finding connects the superior selectivity for C_2+_ products and enhanced longevity of the catalyst to the protection of charged Cu sites.

Despite the achievements in maintaining activity through spatial structure engineering, the incorporation of protective layers to stabilize Cu⁺ sites during eCO_2_RR presents a double‐edged sword. While these layers can indeed enhance stability by shielding Cu⁺ sites from reduction, they may also limit the participation of these active sites in the catalytic reaction, leading to a trade‐off between stability and activity. That said, improving stability might come at the cost of reduced catalytic efficiency. Therefore, striking a balance between the exposure and preservation of active sites remains a key challenge. Further exploration of the catalytic mechanisms occurring beneath the protective layers is essential to optimize this balance. One potential solution is the development of protective layers with selective penetration, allowing specific reactants or intermediates to reach the active sites while maintaining a shield against degradation. Additionally, traditional strategies such as constructing 3D structures to increase the number of accessible catalytic active sites remain promising. These designs can compensate for the potential loss of activity caused by protective layers by providing more surface area and active site availability. In short, to optimize both the selectivity and stability of Cu‐based catalysts, a deeper understanding of the underlying reaction mechanisms is crucial. By combining spatial structure engineering with tailored protective layers and advanced catalytic designs, it may be possible to achieve an improved balance.

### 5.2 Materials Reconstruction Control

In the context of eCO_2_RR, the concept of catalyst reconstruction has emerged as a non‐negligible factor for activity maintenance. Reconstruction of Cu‐based catalysts during the eCO_2_RR is a dynamic process influenced by various factors, including applied bias voltage,^[^ ^151^, ^163^, ^180^, ^527^, ^528^, ^529^ ^]^ interaction with intermediates,^[^ ^530^, ^531^, ^532^, ^533^ ^]^ specifically anion adsorption,^[^ ^482^, ^483^, ^534^, ^535^ ^]^ as well as cell configurations.^[^ ^199^ ^]^ While reconstruction can sometimes lead to performance degradation in some cases, it also presents opportunities to improve catalytic activity and stability by creating specific active sites. Such advantageous reconstruction often involves changes, such as the formation of new crystal faces, grain boundaries, rough surfaces, or alterations in surface morphology and electronic states. Hence, capturing and investigating these phenomena to comprehensively understand how they influence catalysis is conducive to reasonable utilization of surface reconstruction, involving the optimization of catalyst design and improvement of the catalytic efficiency for sustainable energy applications. Conversely, some researchers focus on reconstruction mitigation strategies, aiming to prevent or minimize the undesired reconstruction of the initial high‐active Cu‐based catalysts. This involves various approaches to protect the initially designed materials from undergoing structural changes. Protective coating layers or engineered coordination environments are examples of strategies used to shield catalyst materials from reconstruction. By maintaining the integrity of the catalyst structure, these strategies can ensure the preservation of active sites and facilitate sustained catalytic activity in C_2+_ product generation during eCO_2_RR. In this subsection, we explore both the advantageous reconstruction and reconstruction mitigation strategies for Cu‐based catalysts in the context of eCO_2_RR toward C_2+_ products, offering insights into their impact on activity maintenance. Simulations and in situ investigations of various designed catalysts will be discussed to illuminate the mechanisms underlying the intricate relationship between reconstruction phenomena, selectivity, and stability.

#### 5.2.1 Reconstruction Utilization

The dynamic reconstruction of Cu‐based catalysts during eCO_2_RR has gained significant attention as an essential mechanism for generating new and more stable active sites. This process often involves the transformation of precatalysts into their active forms, reshaping the surface and altering the electronic properties of the catalyst. Notably, the formation of Cu^δ+^ species with a specific valance state, a critical site for promoting C_2+_ product selectivity, is frequently observed as a result of this in situ transformation. Unlike the initial structure, the reconstructed catalyst may possess enhanced catalytic performance due to its adaptive nature under reaction conditions. That said, by continuously evolving during operation, reconstructed catalysts may provide sustained activity, particularly as they often demonstrate improved resistance to deactivation or surface passivation. This adaptability is especially advantageous for eCO_2_RR, where maintaining the delicate balance of surface species is crucial for promoting the formation of C_2+_ products. The dynamic nature of reconstructed catalysts allows them to respond to the changing environment, enabling more efficient and stable catalysis over time.

**Figure 19:**
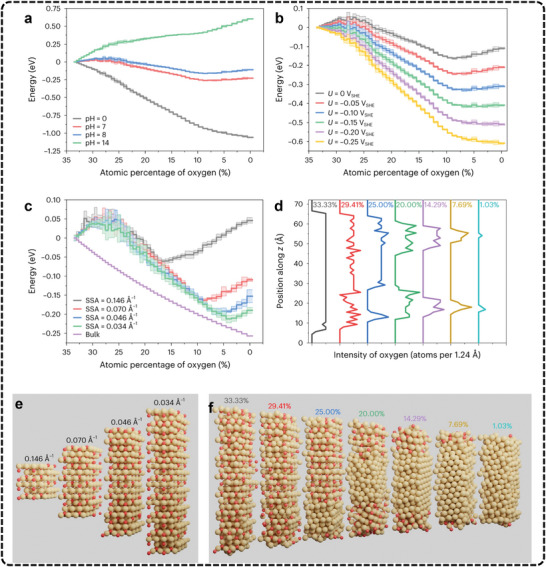
The reduction of Cu_2_O to Cu under different conditions. Adapted under terms of the CC‐BY license.^[^ ^538^ ^]^ Copyright 2024, Lian et al. a–c) System under different pH values, at *U*  =  0 V_SHE_ and SSA  =  0.070 Å^−1^ (a); under different electric potentials versus SHE, at pH  =  8 and SSA  =  0.070 Å^−1^ (b); and with different SSAs, at pH  =  8 and *U*  =  0 V_SHE_ (c). The reaction energies were calculated from the average energy of ten randomly sampled OD‐Cu slabs, using the mean energy during the last 800 ps of equilibration in the annealing simulations (6 ns), and the overall energy was normalized to the reaction energy per unit of Cu_2_O. Dotted lines and transparent regions show the sample standard deviation from these ten samples. The energy diagram of the bulk is derived from the reduction energy from changing bulk Cu_2_O to Cu by using stoichiometry and the DFT static energy instead of MD simulations; d) The distribution of oxygen along the *z‐axis* for the last frame of OD‐Cu (SSA  =  0.034 Å^−1^, sample 1) with different oxygen concentrations. The intensity corresponds to the number of oxygen atoms per unit distance along the z‐axis, and a minor tick interval on the horizontal axis is four atoms per 1.24 Å, a value derived from a perfect Cu_2_O slab model; e) Model of the Cu_2_O slab with different SSAs; oxygen is red, and copper is tan; f) Configurations of OD‐Cu structures corresponding to (d).

Molecular dynamics (MD) simulation is a powerful tool for investigating the structural evolution and dynamics of interfacial species during complex chemical processes such as the eCO_2_RR.^[^ ^102^, ^227^, ^345^, ^536^, ^537^ ^]^ By simulating the atomic‐scale behavior of Cu‐based materials under reaction conditions, MD offers valuable insights into the mechanisms underlying materials evolution and the influence of stability. In recent studies, MD simulations have been employed to elucidate the reconstruction process of Cu‐based catalysts during eCO_2_RR. For example, simulations of the transformation from CuO and Cu_2_O to OD‐Cu have provided key insights into the structural changes occurring at the atomic level.^[^ ^134^ ^]^ Notably, CuO‐derived Cu has been shown to possess a higher density of undercoordinated sites and surface Cu atoms compared to Cu_2_O‐derived Cu, a property that correlates with sustained selectivity toward C_2+_ products during eCO_2_RR. This underscores how the initial structure and evolution of the catalyst directly impact its performance. In another recent study, MD simulations have been utilized to investigate the reduction process of OD‐Cu catalysts and the evolution of active sites during eCO_2_RR.^[^ ^538^ ^]^ These simulations have revealed that the oxygen concentration in OD‐Cu varies with factors such as pH, potential, and specific surface area (SSA). The energy profiles demonstrate that high pH, low applied potential, and larger SSA help mitigate oxygen loss, as depicted in **Figure** **19**a–c. This suggests that optimizing these parameters can help maintain the catalyst activity by preserving crucial oxygen species on the surface. Additionally, while the highly reconstructed Cu surface provides abundant oxygen adsorption sites, enhancing C_2+_ product selectivity (Figure 19d–f), the surface oxygen atoms remain unstable under typical reaction conditions. Despite this instability, removing all trapped oxygen requires significant time, emphasizing the complex nature of the active site environment and the difficulty of precisely identifying active sites in OD‐Cu catalysts, particularly from a temporal perspective. Briefly, MD simulations, when combined with experimental findings, offer valuable references for defining the optimal operating conditions for OD‐Cu catalysts during eCO_2_RR, aiding in the design of more efficient and durable catalysts for eCO_2_RR.

The presence of certain anions in the electrolyte^[^ ^290^, ^470^, ^486^ ^]^ or adsorbed on the electrode surface^[^ ^488^, ^489^ ^]^ has emerged as a critical determinant in the dynamic reconstruction of Cu‐based catalysts during eCO_2_RR. These anions, particularly halide ions, play a significant role in influencing the catalyst's structural evolution, which in turn impacts both the selectivity and stability of the system. Halide ions, such as those in cuprous halide compounds, participate in dynamic leaching and reabsorption processes during eCO_2_RR, leading to the formation of reconstructed Cu‐based catalyst structures with diverse morphologies and oxidation states.^[^ ^470^, ^482^, ^483^ ^]^ For instance, Cu‐CuI composites and AgI‐incorporated CuO catalysts exhibit significant structural changes during eCO_2_RR, enhancing their activity for generating C_2+_ products.^[^ ^489^, ^490^ ^]^ Importantly, these reconstructed catalysts are reported to possess stable in situ‐formed structures, contributing to sustained activity. One key feature of these reconstructions is the presence of residual Cu^δ+^ species, which co‐exist with Cu^+^ and Cu^0^ states. These species are pivotal in facilitating C─C coupling reactions. This enhanced understanding of halide‐induced reconstruction offers valuable insights into optimizing the design and operation of Cu‐based catalysts to achieve higher efficiency and selectivity in energy conversion applications.

Besides halide‐modified catalysts, the reconstruction phenomena have been recently observed and utilized in other Cu‐based catalyst systems as well. For instance, CeO_2_‐modified Cu electrodes undergo oxidative corrosion during eCO_2_RR conditions, leading to the formation of an active and stable CuO_x_ layer with distinct Cu sites that promote sustained C─C coupling and the formation of C_2+_ products.^[^ ^514^ ^]^ Similarly, Cu‐CeO_2_ nanorods have been investigated as eCO_2_RR catalysts, where they undergo reconstruction to form stable nanoclusters.^[^ ^515^ ^]^ These nanoclusters exhibit a coexistence of Cu^0^ and Cu^+^ species, which can sustainably catalyze the production of C_2+_ products. In addition, molecularly modified Cu electrodes also display sensitivity to dynamic electrochemical environments, such as fluctuating potentials and in situ generated intermediates. These factors may disrupt the modifications, triggering surface rearrangement and restructuring of the Cu catalyst. For example, methanethiol‐modified Cu electrodes have demonstrated surface reconstruction, leading to the formation of a rough Cu surface as a result of the reduction of the molecular modifications.^[^ ^496^ ^]^ This reconstructed surface has been shown to remain stable and active, significantly enhancing the generation of C_2+_ products during eCO_2_RR. Wu et al., recently introduced a strategy to improve the stability of Cu nanoparticles in MEA by incorporating activated carbon black with different functional groups.^[^ ^539^ ^]^ They observed that the in situ generation of OH radicals from water creates a locally oxidative microenvironment on the Cu surface during eCO_2_RR, leading to the formation of stable high‐curvature nanowhiskers. These nanowhiskers, along with stabilized Cu^δ+^‐OH species, ensured stable operation for over 35 h in a 5 cm^2^ MEA reactor at 300 mA cm^−2^ for C_2_H_4_ production.

**Figure 20:**
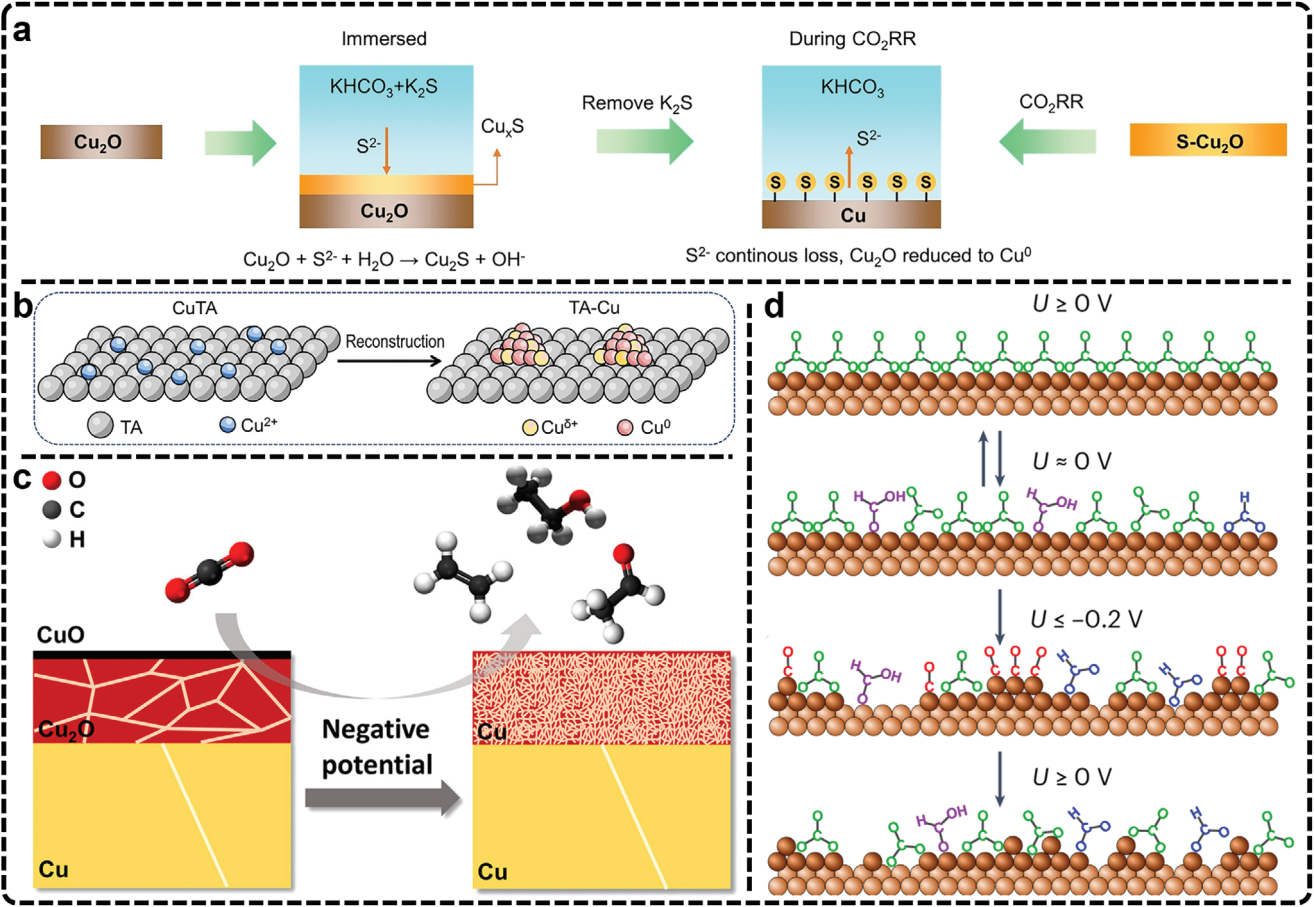
a) Schematic illustration showing dynamic equilibrium of S at the catalyst‐electrolyte interface. Reproduced with permission.^[^ ^405^ ^]^ Copyright 2023, John Wiley and Sons. b) Preparation of TA‐Cu via electrochemical reconstruction of CuTA. Reproduced with permission.^[^ ^430^ ^]^ Copyright 2023, John Wiley and Sons. c) Schematic illustration showing the fragmentation of oxides in Cu‐based electrodes (containing Cu, Cu₂O, and CuO) into nanosized irregular Cu grains under applied negative potentials. Reproduced with permission.^[^ ^207^ ^]^ Copyright 2020, American Chemical Society. d) Schematic of Cu reconstruction. Starting from a planar Cu surface with an ordered carbonate adlayer, decreasing the potential (*U*) leads to a reversible transition to a disordered carbonate/carboxylate adlayer, followed by CO‐induced formation of Cu nanoclusters and vacancy islands. Increasing the potential back to the double‐layer regime, the Cu clusters disperse into isolated Cu adatoms, resulting in a disordered adlayer of mutually stabilizing carbonate/carboxylate adsorbates and Cu surface defects. Adapted under terms of the CC‐BY license.^[^ ^529^ ^]^ Copyright 2023, Amirbeigiarab et al.

Moreover, reconstruction plays a vital role in materials preparation, as seen in S‐doped Cu_2_O, which undergoes in situ surface reconstruction during eCO_2_RR to generate active S‐adsorbed metallic Cu sites (**Figure** **20**a).^[^ ^405^ ^]^ Similarly, TA‐Cu are prepared through the in situ electroreduction of CuTA (Figure 20b).^[^ ^430^ ^]^ The reconstructed TA‐Cu catalysts feature Cu particles embedded with numerous hydroxyl species, where the hydroxyl‐modified Cu particles exhibit Cu─O species that keep stable during eCO_2_RR. In a recent report, Zhang et al., introduced an in situ electrochemical reconstruction strategy to generate low‐coordinated Cu sites at the Cu/Cu_2_O interface from a Cl‐doped Cu_2_O catalyst.^[^ ^141^ ^]^ This stable structure achieved a consistent C_2_H_5_OH FE of over 50% during continuous operation for 50 h. In short, these in situ engineered Cu‐based catalysts exhibit adaptability under operational conditions, preventing the blockage of active sites and guiding relevant reaction intermediates to ensure continuous high selectivity. This adaptability underscores the significance of surface reconstruction in tailoring catalyst properties, fostering sustained C_2+_ product generation.

Cu‐based catalysts with specific crystal facets or grain boundaries have been extensively investigated for their potential to promote C_2+_ production. Particularly, stepped Cu facets have been predicted and validated to possess moderate ^*^CO binding energies, endowing them with ultra‐high reactivity in enhancing C_2_H_4_ selectivity.^[^ ^98^, ^294^, ^540^, ^541^, ^542^ ^]^ Unfortunately, one notable challenge arises from the stability of these stepped Cu facets in aqueous solutions, as they are susceptible to reconstruction into less active Cu (111) facets after a few hours of eCO_2_RR, leading to performance deterioration.^[^ ^68^, ^252^ ^]^ Interestingly, some recent studies present contrasting results. For instance, investigations into oxide‐/hydroxide‐derived Cu surfaces have shown that they undergo fragmentation into nanosized irregular Cu grains under applied negative potentials during eCO_2_RR.^[^ ^207^ ^]^ This fragmentation process, driven by an oxidation‐reduction cycle, results in the formation of an intricate network of grain boundaries and exposure of various high‐index facets (Figure 20c). Despite the transformation of oxidized Cu species into metallic Cu, the catalyst retains activity. These unique structural features significantly promote C─C coupling, thereby producing C_2+_ products over extended operation periods. In another work, Cuenya et al., explored the morphological reconstruction of Cu catalysts under pulsed electrolysis conditions, where the reconstruction led to highly defective interfaces and grain boundaries, significantly enhancing C_2+_ product selectivity.^[^ ^406^ ^]^ This dynamic potential‐driven method is promising since controlling the oxidation‐reduction cycle can ingeniously create active sites, preventing activity loss. In related work, Magnussen et al., have used in situ techniques such as scanning tunneling microscopy, surface X‐ray diffraction, and Raman spectroscopy to reveal the formation of low‐coordinated Cu atoms during pulsed electrolysis of eCO_2_RR.^[^ ^529^ ^]^ As illustrated in Figure 20d, the dynamic evolution of Cu sites begins with the formation of CO‐induced Cu nanoclusters in the early stages of the reaction, leading to irreversible surface restructuring that remains stable across a wide potential range. Upon further potential increases, these nanoclusters disperse into Cu adatoms, which subsequently stabilize reaction intermediates on the surface. The self‐driven creation of these low‐coordinated sites on the Cu surface contributes to its catalytic activity and could potentially be harnessed to reactivate CO_2_ reduction sites through potentiodynamic techniques. Consequently, the activity can be maintained over extended operational periods. These recent studies highlight the crucial role of in situ reconstruction in creating defective morphology and exposing various facets to promote stable C_2+_ product formation, showing the potential of strategically leveraging in situ reconstruction to optimize catalytic performance.

Overall, these findings show the complex nature of catalyst reconstruction during eCO_2_RR and its profound impact on catalytic performance. Understanding and manipulating these reconstruction processes can lead to the design of more efficient catalysts with tailored selectivity and stability for eCO_2_RR applications. Integration of computational predictions, experimental observations, and surface modification strategies offers avenues for advancing our understanding of catalyst reconstruction mechanisms and developing novel catalyst designs for sustainable CO_2_ conversion technologies. Further investigations are needed to fully elucidate the mechanisms underlying reconstruction and its impact on product selectivity, as reconstruction not only leads to changes in one property, such as the electronic state of Cu and morphology may also simultaneously be altered under in situ reconstruction. Moreover, the incorporation of surface functionalization strategies such as organic ligand modification further complicates the evolution of materials under operational conditions. Potentiodynamic techniques are valuable in order to maintain long‐term operational stability by either utilizing favorable active species or mitigating the formation of unfavorable sites. However, precisely controlling the potential intervals and ranges to align with the dynamics of surface evolution remains an area that requires further in‐depth exploration. This necessitates a comprehensive investigation into the reconstruction dynamics across various catalysts to optimize their performance and stability.

#### 5.2.2 Reconstruction Mitigation

On the other hand, there is a growing interest in developing strategies to prevent or mitigate the reconstruction of Cu‐based catalysts during eCO_2_RR. These approaches aim to maintain the intrinsic activity of the catalysts by preserving their initial surface structure and components. Surface coating and coordination engineering are two prominent strategies employed to achieve this goal in recent works. Surface coatings, such as metal oxides or organic layers, act as protective barriers, shielding the catalyst surface from structural changes induced by reaction conditions. However, these strategies may alter the distribution and accessibility of active sites, making it crucial to balance catalytic activity with long‐term stability. Coordination engineering focuses on controlling the coordination environment of surface Cu atoms to stabilize their electronic states and prevent surface restructuring. This can be achieved through heteroatom incorporation or designing Cu‐based complexes, which help maintain the desired coordination environment, ultimately preserving both the selectivity and stability of the catalyst.

**Figure 21:**
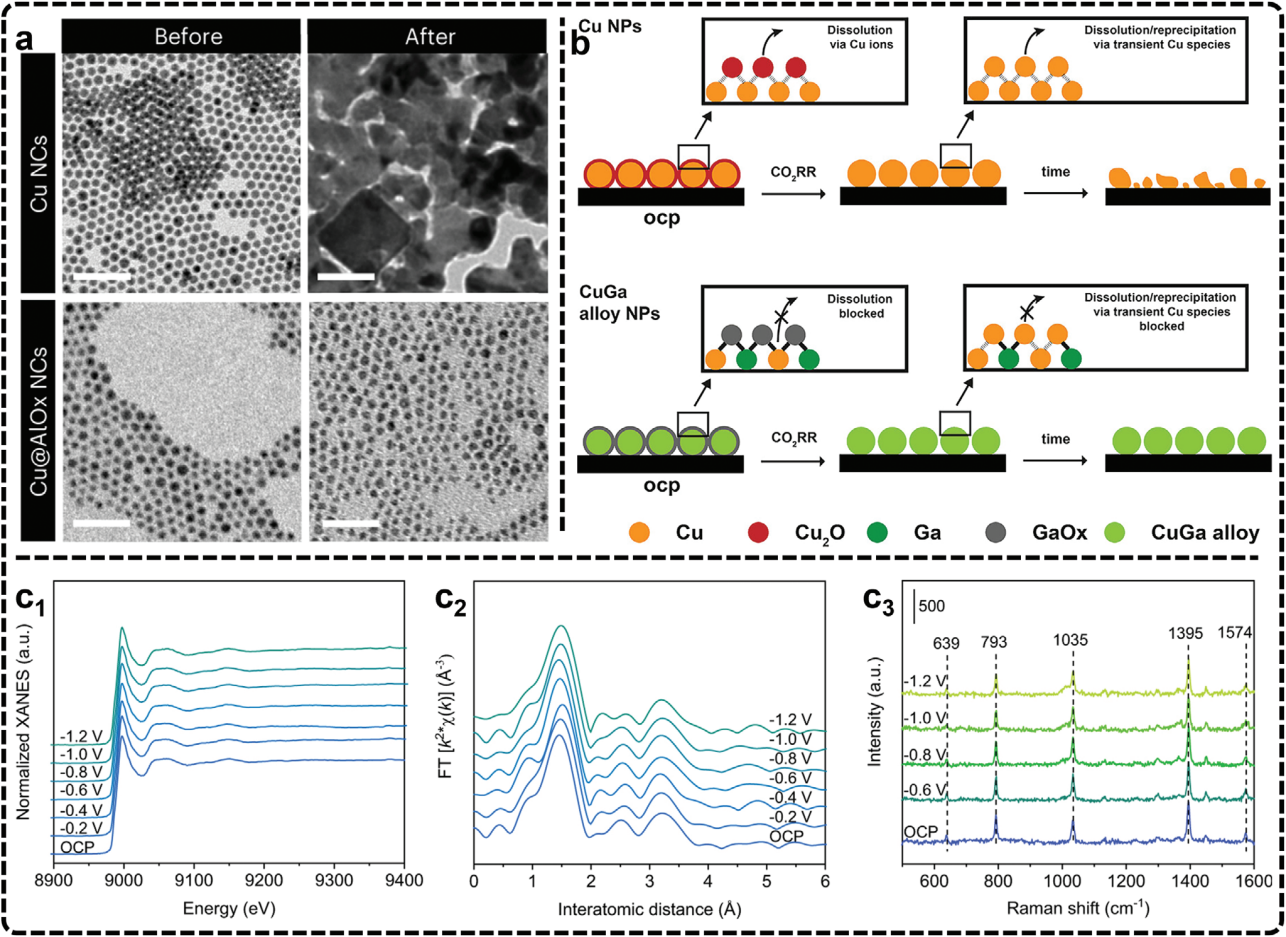
a) Representative BF‐TEM image of Cu nanocrystals and Cu@AlO_x_ nanocrystals before and after 4 h eCO_2_RR at −1.1 V versus RHE, respectively. Scale bars, 50 nm. The as‐synthesized Cu nanocrystals transform into aggregated structures, with CuO cubes forming upon air exposure. Adapted under terms of the CC‐BY license.^[^ ^521^ ^]^ Copyright 2024, Albertini et al. b) Illustration of the eCO_2_RR stabilization mechanism in CuGa alloy nanoparticles compared to Cu nanoparticles. Reproduced with permission.^[^ ^545^ ^]^ Copyright 2023, American Chemical Society. Operando Cu K‐edge c_1_) XANES and c_2_) EXAFS of Cu(OH)BTA at the applied potentials of −0.2 to −1.2 V under CO_2_ atmosphere, as well as c_3_) in situ Raman spectra of Cu(OH)BTA at the applied potential of −0.6 to −1.2 V under CO_2_ atmosphere.^[^ ^64^ ^]^ The peaks at 639, 793, 1035, 1395, and 1574 cm^−1^ correspond to the vibrational modes of molecular structure in Cu(OH)BTA. The slight changes in these spectra are indicative of the high stability of the Cu oxidation state and coordination structure. Adapted under terms of the CC‐BY license.^[^ ^64^ ^]^ Copyright 2023, Liang et al.

In addition to the protection of charged Cu active sites discussed earlier, applying a shell or coating is also presented significantly to safeguard Cu‐based catalysts against reconstruction in both morphology and composition, contributing to the continuous generation of C_2+_ products in the eCO_2_RR. Research in this area is dedicated to addressing the challenge of maintaining initial selectivity that suffers from limited stability over time. For instance, Ma et al., have investigated the stability of Cu catalysts in an operational eCO_2_RR environment and identified hydroxides in electrolytes as key factors influencing the morphology evolution of Cu catalysts, consequently leading to a gradually decreased FE of C_2+_ products over time.^[^ ^543^ ^]^ To mitigate this issue, they have designed a porous carbon overlay deposited on Cu catalysts, demonstrating that the morphology of Cu catalysts and the FE of products are effectively preserved during the reaction. Further investigations explain that the added carbon overlay can resist the influence of hydroxide corrosion on Cu catalysts. Another study investigates the morphological evolution of polycrystalline Cu and graphene‐covered polycrystalline Cu surfaces during potentiostatic polarization at eCO_2_RR potentials for several hours.^[^ ^528^ ^]^ The results reveal that graphene serves as a protective barrier against poisoning and deactivation of Cu catalysts, while Cu catalysts are highly prone to poisoning and deactivation during the first 30 min in eCO_2_RR. Beyond carbon‐based coatings, a unique AlO_x_ shell has also been shown to resist reconstruction.^[^ ^521^ ^]^ As observed by TEM (**Figure** **21**a), the AlO_x_‐covered Cu catalyst mitigates the aggregation of initially dispersive Cu nanocrystals, while uncoated Cu nanocrystals transform into aggregated structures with Cu oxide cubes formation upon air exposure. Likewise, Zhu et al., proposed an “armor protection” strategy by coating Cu surfaces with an ultrathin, hydrophobic SiO_2_ layer.^[^ ^544^ ^]^ The SiO_2_ layer prevented structural reconstruction and preserved Cu^δ+^ active sites by hindering K^+^ ion accumulation and restricted OH^−^ diffusion, both of which enhanced sustained C_2+_ product formation for over 12 h in acidic environments. These examples provide novel approaches to preventing undesired structural changes and enhancing the long‐term stability of Cu‐based catalysts in eCO_2_RR.

In the above subsection, several successful cases demonstrate that incorporating hetero elements is highly effective in stabilizing the oxidation states of Cu. This is due to certain hetero elements exhibiting a high affinity for oxygen or other ligands, which is crucial for maintaining the active sites and facilitating the sustained formation of C_2+_ products. In fact, introducing hetero elements, particularly from the main group, into Cu‐based catalysts may also significantly enhance the coordination bonds between Cu atoms and these heteroatoms. Such reinforcement may strengthen the chemical stability of Cu, reducing the mobility of Cu atoms and thereby limiting the diffusion and dissolution of Cu under bias voltage during eCO_2_RR. Consequently, this incorporation helps prevent undesired surface reconstruction or aggregation of Cu atoms, maintaining the catalyst's structural integrity over extended periods. Chen et al., have reported a reconstruction‐resistant CuSiO_x_ amorphous nanotube catalyst with abundant atomic Cu─O─Si interfacial sites in catalyzing CO_2_ reduction.^[^ ^469^ ^]^ This catalyst exhibits strong interfacial interactions between Cu and Si, rendering the Cu─O─Si interfacial sites ultra‐stable during eCO_2_RR without undergoing apparent reconstruction during the test for 12 h. Similarly, alloying Cu with Ga enhances the stability of CuGa alloy nanoparticles compared to pure Cu nanoparticles.^[^ ^545^, ^546^ ^]^ Buonsanti et al., have demonstrated that CuGa alloy nanoparticles containing 17 at% Ga retain most of their eCO_2_RR activity for at least 20 h, whereas Cu nanoparticles of similar size undergo reconstruction and lose their activity within 2 h.^[^ ^545^ ^]^ The stabilization of Cu by Ga is attributed to the electronic interactions between the two elements, primarily driven by the higher oxophilicity and lower electronegativity of Ga. These properties help inhibit the dissolution and reprecipitation of Cu atoms during eCO_2_RR (Figure 21b), thereby maintaining the structural integrity and catalytic performance of the catalyst surface over extended reaction times. It can be seen that introducing elements with high chemical affinity for oxygen, such as Si and Ga, offers a promising strategy to stabilize Cu‐based catalysts. The formation of strong Cu–heteroatom bonds can suppress in situ reconstruction, thus maintaining the selectivity during eCO_2_RR.

Cu‐based complex catalysts offer exceptional tunability for converting CO_2_ into C_2+_ products due to their flexible molecular design. However, their structural stability poses a significant challenge as well. The rational selection of coordination groups is crucial, as the strength of the metal‐ligand bond directly influences the structural stability under eCO_2_RR conditions. Strong bonds can mitigate the effects of potential‐driven structural changes and help maintain catalytic performance.^[^ ^547^, ^548^ ^]^ One typical strategy involves the incorporation of thiol end groups, known for their stable binding to Cu even under electrochemical reducing conditions.^[^ ^494^, ^495^ ^]^ Recent research has demonstrated the efficacy of dodecanethiol modification in enhancing C_2_H_4_ activity on CuO nanoelectrodes and stabilizing favorable Cu facets from in situ reconstruction for 40 h, highlighting its potential to improve catalyst longevity.^[^ ^67^ ^]^ Sakamoto et al., have presented a Br‐bridged dinuclear Cu(I) complex for eCO_2_RR, showcasing high robustness during the reaction.^[^ ^82^ ^]^ This study reveals that the metal complex structure remains intact without reconstruction throughout the eCO_2_RR, thereby resisting the formation of Cu clusters and maintaining the Cu(I) oxidation state. This corroborates the ability of the complex to withstand reconstruction and ensure sustained catalytic activity. A similar protective layer has recently been reported, for instance, 4‐dimethylaminopyridine (DMAP).^[^ ^549^ ^]^ Another approach involves the utilization of Cu dendrites with carboxylate‐bridged Cu(II) coordination, which interact strongly with Cu (110) facets to stabilize the microstructure of the electrode.^[^ ^468^ ^]^ This interaction helps to maintain the valence of subsurface Cu atoms in a moderate oxidation state, preventing self‐reduction and performance decay during prolonged eCO_2_RR operation. Notably, stable electrolysis for 400 h at 800 mA cm^−2^ with continuous production of C_2_H_5_OH is achieved to show enhanced stability. Cu‐based single‐site catalysts are also prone to in situ reconstruction into metallic agglomerations under working conditions.^[^ ^550^, ^551^, ^552^ ^]^ To address this challenge, Zeng et al., have reported a stable Cu single‐site Cu coordination polymer (Cu(OH)BTA) with periodic neighboring Cu to enhance the C_2_H_4_ selectivity and production stability.^[^ ^64^ ^]^ Operando spectroscopic analyses, including XAS and Raman scattering, reveal that the catalyst remains structurally stable under different bias voltages during the reaction (Figure 21c), ensuring sustained CO_2_ electrolysis for an extended period. In another recent work, Dai et al., introduced a “charge release” method to tackle the deactivation issues of Cu catalysts, such as unstable alkalinity and carbonate buildup due to unwanted ion movement.^[^ ^553^ ^]^ They incorporated a small amount of the anionic ionomer perfluorinated sulfonic acid (PFSA) into a cationic covalent organic framework (cCOF) on the Cu surface. This addition stabilizes positively charged ions, preventing the escape of OH^−^ and minimizing carbonate formation, thus creating a stable alkaline environment. The hydrophobic nature of the ionomer also enhances gas transport. This design achieves a FE of up to 70% for C_2_H_4_ and maintains exceptional stability for over 760 h.

However, despite the progress made in stabilizing specific coordinated Cu sites, only a limited number of complex groups or linkers have been recognized in the literature. A systematic summary of how these groups or linkers, when bonded to Cu sites in Cu‐based catalysts, influence both stability and product selectivity is currently lacking. Most of the current research in this area follows a trial‐and‐error approach, which hinders the advancement of this field. A more structured understanding of molecular design strategies and their effects on catalyst performance is needed to drive innovation and optimization in eCO_2_RR. On the other hand, reconstruction processes also pose kinetic challenges. Initial reconstruction may occur slowly, leading to negligible impact on activity initially, but substantial effects may emerge after prolonged use, such as 10 h or more. For example, seemingly stable Cu single‐atom catalysts have been reported to reconstruct in situ into clusters or particles after prolonged use.^[^ ^550^, ^551^ ^]^ Occasionally, Cu single atoms can aggregate into clusters under in situ conditions and disperse back into single atoms once the power is cut off, indicating the complication of accurate identification of active sites.^[^ ^552^ ^]^ Therefore, combining in situ monitoring with post‐reaction characterization is crucial for accurately identifying true active sites. Furthermore, the mechanisms of reconstruction remain debatable, for example, the dissolution‐redeposition and intermediate‐driven transfer mechanisms.^[^ ^529^, ^532^ ^]^ Understanding these processes and developing strategies to either rationally utilize or mitigate reconstruction are essential for creating more effective and stable catalysts for eCO_2_RR.

## 6 Conclusion and Perspectives

In recent years, substantial progress has been made in the design of Cu‐based catalysts for the electrochemical conversion of CO_2_ into C_2+_ products and offers valuable insights that bring the field closer to industrial fruition. This review highlights recent key advances in optimizing Cu‐based catalysts to enhance C_2+_ selectivity and operational stability. It begins by discussing both conventional and newly proposed reaction pathways for C_2+_ product formation, which, while highly dependent on specific catalytic sites, provide essential guidance for future mechanism exploration. As many of these pathways are derived from theoretical predictions, in situ, techniques are crucial for experimental validation, particularly for detecting intermediates and observing material evolution in real time. By discussing these commonly employed in situ techniques, the review emphasizes strategies to improve C_2+_ selectivity, with a focus on materials design and methods aimed at manipulating key intermediates to promote C─C coupling. Two key strategies, enhancing surface ^*^CO coverage and optimizing intermediate protonation, are highlighted for their importance in improving C─C coupling efficiency. In addition, a major challenge is the instability of Cu‐based catalysts under the reducing conditions of eCO_2_RR, which often leads to the loss of positively charged Cu species and materials reconstruction. Maintaining active sites is crucial to sustaining C_2+_ selectivity. Therefore, this review also addresses strategies for preserving catalytic activity, focusing on approaches that manage material evolution to retain charged Cu species and control reconstruction, both critical for sustained performance. Finally, Table 1 provides a detailed summary of recently representative Cu‐based electrocatalysts for eCO_2_RR to C_2+_ products, cataloging key intermediates, main products, FE, current densities (*j*), operational durations, and types of electrolytic cells and electrolytes. This comprehensive overview provides valuable guidance for future catalyst design by combining mechanistic understanding with electrochemical performance assessment. Nevertheless, despite recent advancements, significant challenges remain before industrial scalability can be achieved.

### 6.1 Reaction Pathways and In Situ Techniques

A significant challenge in advancing the eCO_2_RR to C_2+_ products is understanding the complex reaction pathways involved. The process requires precise control over multiple steps, including CO_2_ adsorption, electron and proton transfers, and C─C coupling, each governed by thermodynamic and kinetic factors. However, current experimental techniques have limited ability to probe interactions between catalysts and intermediates at the atomic or electronic level. Traditional characterization techniques struggle to capture detailed information about transient intermediate states and the evolving catalyst surface during reactions. This limitation hampers the identification of key intermediates and active sites that are critical for understanding reaction pathways. Theoretical computations, while useful in compensating for experimental gaps, face their own limitations. They can explore the transformation of intermediates, the evolution of reaction centers, and the effects of ions and electrolytes, but accurately modeling the electrolyte–electrode interface remains a challenge. Explicit solvent models are time‐consuming and complex, while implicit models risk missing important interactions like hydrogen bonding. Additionally, refining charge transfer models, particularly for constant‐charge systems, is necessary to improve simulation accuracy.

To overcome these challenges, integrating advanced in situ characterization techniques is crucial. These methods provide real‐time insights into catalyst behavior and intermediate dynamics, helping to establish clearer structure‐performance relationships. However, in situ techniques currently suffer from weak signal intensities due to the low coverage of key intermediates and difficulty capturing the full structural evolution of catalysts during reactions. To improve the accuracy of observations, multiple in situ techniques should be applied simultaneously within the same reactor. Combining microscopy with spectroscopic methods like XAS, FTIR, or Raman spectroscopy in real time can provide more convincing data on both structural changes and intermediate dynamics. In addition, a further challenge in in situ experiments lies in the design of electrolytic cells. These cells often require structural modifications to withstand the harsh physical and chemical conditions of in situ testing, such as spatial constraints and potential laser‐induced damage to materials, leading to discrepancies between the testing environment and actual reaction conditions. Developing optimized electrolytic cells that more closely replicate real working conditions is essential for obtaining results that accurately reflect the true behavior of eCO_2_RR systems.

Looking forward, combining in situ experiments with theoretical calculations holds great promise for overcoming these obstacles. This integrated approach will accelerate the prediction and screening of new catalysts while providing deeper insights into reaction thermodynamics, kinetics, and pathways. These advances will lead to more effective and rationally designed Cu‐based catalysts for eCO_2_RR to C_2+_ products.

### 6.2 Product Selectivity

Achieving high selectivity for C_2+_ products in eCO_2_RR is a significant challenge due to the complex, multi‐step nature of the reaction, particularly the C─C coupling step. Competing side reactions, such as HER, often reduce the selectivity and yield of desired C_2+_ products. Enhancing selectivity requires precise regulation of the catalyst surface to promote C─C bond formation while suppressing unwanted side reactions like HER or CH_4_ production. C_2+_ product formation in eCO_2_RR is closely linked to the concentration and surface coverage of ^*^CO intermediates. Higher ^*^CO coverage promotes the proximity of carbon species, which is essential for efficient C─C coupling, a thermochemical step driven by mass transfer conditions rather than electron or proton transfer. To increase ^*^CO coverage, catalyst design must focus on tuning the electronic structure and surface properties. Adjusting crystal facets, introducing surface defects, or modifying the coordination environment can alter the adsorption energy of key intermediates like ^*^CO, helping to break the scaling relationships that limit catalytic performance and promote C_2+_ product formation. Additionally, incorporating metals with high CO selectivity, such as Au, Ag, or Zn, in a tandem catalysis system can enhance C_2+_ production and should be vigorously pursued as a promising strategy. In this approach, CO_2_ is reduced to CO on a CO‐selective metal surface, and then CO diffuses to an adjacent metal site for C─C coupling and further reduction. The multi‐metal cooperation maintains a high CO concentration on the catalyst surface and steers the reaction toward C_2+_ products while inhibiting side reactions.

Another key strategy to improve C_2+_ product selectivity involves optimizing the protonation of intermediates. In eCO_2_RR, the type of protonated intermediates, such as ^*^CHO, ^*^COH, or ^*^CH_x_, determines the pathway and efficiency of C─C bond formation. By optimizing the ^*^H coverage to protonate key ^*^C_1_ intermediate, it is possible to influence the coupling mode, leading to a lower energy C─C coupling process and altering the product distribution. This protonation optimization can be achieved by heteroatom functionalization, oxide and hydroxide introduction, or organic complex linking. Heteroatom doping or surface modification with oxides and hydroxides fine‐tunes the local catalyst environment and fosters favorable interactions between intermediates and active sites. These modifications enhance the stability of key intermediates and adjust the protonation process to favor the production of C_2+_ products. Additionally, organic linkers with well‐defined functional groups provide new opportunities to improve intermediate protonation. This approach can produce novel hybrid catalysts that combine the benefits of molecular and heterogeneous systems, further boosting C─C coupling efficiency. Future research should explore a wider range of organic linkers with diverse functional groups to unlock their full potential in improving protonation and C_2+_ product formation.

The success of these strategies, whether by increasing surface ^*^CO coverage or optimizing intermediate protonation, depends on the ability to finely tune catalyst surfaces and reaction environments on the atomic level. As research advances, integrating machine learning and in situ characterization techniques will be critical to designing next‐generation Cu‐based catalysts with improved selectivity for C_2+_ products. These tools will enable rapid screening of catalyst structures and conditions and provide deeper insights into C─C coupling mechanisms and more effective catalyst design strategies. Finally, while current research primarily focuses on C_2_ products, exploring the formation of higher‐order C_3+_ products presents a promising frontier. Achieving this requires catalysts capable of supporting multi‐step C─C coupling reactions and maintaining high ^*^C_2_ intermediate coverage, which poses significant challenges for both catalyst and reactor design. Cascade catalytic systems and integrated approaches, combining light, heat, and electrochemical methods, could expand the range of products derived from CO_2_ conversion.

### 6.3 Catalyst Stability

Achieving long‐term stability for Cu‐based catalysts in eCO_2_RR is crucial, particularly for the production of C_2+_ products. Although charged Cu species are highly effective in promoting C─C coupling, they are prone to reduction to metallic Cu under the reducing conditions of eCO_2_RR, resulting in diminished catalytic activity. Protecting these active species is a key challenge. While recent approaches show promise in preserving Cu^δ+^ and maintaining their stability over longer periods, particularly under operational conditions, remains difficult. This is because current materials design and methods may slow Cu^δ+^ reduction but do not completely prevent it. Another major factor influencing the catalyst stability is materials reconstruction, in which the catalyst undergoes structural and compositional changes during the reactions. This process can either enhance or degrade performance. Traditionally viewed as detrimental, recent studies have shown that some in situ reconstructed catalysts can adapt to the reaction environment, leading to improved stability and performance. This presents a novel opportunity to enhance the stability by embracing in situ reconstructed Cu‐based catalysts, which can better withstand the demanding conditions of eCO_2_RR. In cases where reconstruction compromises stability, common mitigation strategies include designing catalysts with stable crystalline structures or protective layers to resist structural changes. Encapsulating active Cu sites in robust matrices or using heterostructures can also reduce reconstruction. However, like the efforts to protect Cu^δ+^ species, these methods may only mitigate, rather than fully eliminate reconstruction, especially over extended periods. Most studies focus on short‐term performance evaluations, typically under 100 h, which are insufficient to fully assess long‐term stability.

A recurring issue in stabilizing Cu‐based catalysts is the trade‐off between stability and activity. Protective layers can shield Cu^δ+^ from reduction or mitigate reconstruction, but they may also block active sites from participating in the catalytic reaction. Balancing the need for exposure of active sites with their preservation is a key challenge. One potential solution is to develop protective layers that selectively allow reactants to penetrate while preventing degradation. Furthermore, designing 3D structures that increase the number of accessible active sites could help offset any loss of activity caused by protective measures. In materials evolution kinetic aspect, many studies evaluate catalysts over short periods, missing critical insights into long‐term deactivation. In industrial applications, catalysts must remain reliable over extended periods. In situ techniques are vital for real‐time monitoring of catalyst behavior under working conditions and impart valuable information about the structural and activity changes with time. Continuous monitoring can reveal early signs of deactivation, helping to develop more effective stability strategies and refine catalyst designs for sustained use. Briefly, while ensuring the long‐term stability of Cu‐based catalysts for eCO2RR remains a significant challenge, achieving industrial viability will require further refinement of protective strategies, development of in situ reconstructed catalysts, and advanced monitoring techniques to better understand and mitigate long‐term deactivation.

## Conflict of Interest

The authors declare no conflict of interest.
